# Uncovering RNA binding proteins associated with age and gender during liver maturation

**DOI:** 10.1038/srep09512

**Published:** 2015-03-31

**Authors:** Praneet Chaturvedi, Yaseswini Neelamraju, Waqar Arif, Auinash Kalsotra, Sarath Chandra Janga

**Affiliations:** 1 Department of BioHealth Informatics, School of Informatics and Computing, Indiana University Purdue University, 719 Indiana Ave Ste 319, Walker Plaza Building, Indianapolis, Indiana 46202; 2 Departments of Biochemistry and Medical Biochemistry, University of Illinois, Urbana-Champaign, Illinois 61801, USA; 3 Center for Computational Biology and Bioinformatics, Indiana University School of Medicine, 5021 Health Information and Translational Sciences (HITS), 410 West 10th Street, Indianapolis, Indiana, 46202; 4 Department of Medical and Molecular Genetics, Indiana University School of Medicine, Medical Research and Library Building, 975 West Walnut Street, Indianapolis, Indiana, 46202

## Abstract

In the present study, we perform an association analysis focusing on the expression changes of 1344 RNA Binding proteins (RBPs) as a function of age and gender in human liver. We identify 88 and 45 RBPs to be significantly associated with age and gender respectively. Experimental verification of several of the predicted associations in mice confirmed our findings. Our results suggest that a small fraction of the gender-associated RBPs (~40%) are expressed higher in males than females. Altogether, these observations show that several of these RBPs are important and conserved regulators in maintaining liver function. Further analysis of the protein interaction network of RBPs associated with age and gender based on the centrality measures like degree, betweenness and closeness revealed that several of these RBPs might be prominent players in aging liver and impart gender specific alterations in gene expression via the formation of protein complexes. Indeed, both age and gender-associated RBPs in liver were found to show significantly higher clustering coefficients and network centrality measures compared to non-associated RBPs. The compendium of RBPs and this study will help us gain insight into the role of post-transcriptional regulatory molecules in aging and gender specific expression of genes.

Gene expression changes dynamically throughout the lifetime of an organism and the sub set of proteins expressed at each point in time allows cells to carry out important functions such as response to external stimuli, cell differentiation and development. These age related expression changes would influence the functioning of an organism. A study of post-mortem human brain tissue from 30 individuals aged 26 to 106 years showed that approximately 4% of the 11,000 genes analysed show a significant age related expression change^1^. Another independent study examined healthy renal tissue removed at nephrectomy from 74 patients ranging in age from 27 to 92 years to identify ~1000 genes to be differentially expressed with age^2^. In addition, a significant difference in the expression of several genes encoding for antioxidant and detoxifying enzymes was seen in aged livers of both rats and human^3^. More recently, a study on age-dependent gene expression changes in 5 different tissues showed skin to have the most age related gene expression changes^4^. Similar to the age-related expression changes, it is also seen that genes express differently in the same organs of male and female. Recently, it was reported that though male and females share high similarity at genome level, most of the dimorphic traits are constrained to happen based on sex-biased gene regulation^5^. Another study that studied the sex based differences in the transcriptome of the human blood identified a gender specific expression in 582 autosomal genes of which 57.2% were up regulated in females^6^. It is also suggested that the gender based differences in epigenetic mechanisms may have profound consequences on brain development^7^.

Although, the expression of genes varies based on age and gender, molecular mechanisms causing these differences still remain unclear. Transcriptome changes could be largely attributed to difference in the levels of regulators participating at various stages of gene expression. One such class of regulatory molecules are the RNA Binding Proteins (RBPs)-that bind RNA molecules to control different post transcriptional processes such as pre-mRNA splicing, mRNA cytoplasmic export, turnover, storage, and translation^8^,^9^. Thus, the capacity of these proteins to influence gene expression at post-transcriptional level is extremely important especially during the developmental process to give rise to complex organs and tissues^10^,^11^. For instance, PTBP1 (polypyrimidine tract binding protein), a ubiquitous protein known to be important in mammalian development at early stages of gastrulation^12^,^13^ and ELAVL1 (HuR) - a protein that acts as an mRNA stability factor, is also known for its role in placental branching, embryonic and neuronal development^14^,^15^. Likewise, CRD-BP (IGF2BP1), a member of the insulin-like growth factor 2 mRNA-binding protein family, is the first example of a putative mammalian mRNA-binding proteins that is abundant in fetal tissue but absent in the adult tissue^16^.

As is evident from the above examples, RBPs play a substantial role in mediating developmental changes of a mammalian cell. Furthermore, a study on understanding the regulation of HNF4alpha in liver development revealed that the expression of HNF4aplha is widely regulated by the sequential promoter usage and alternative splicing in the 3′ end to produce different isoforms important for the liver development^17^. Yet another study identified UPF2, one of the key players of the nonsense-mediated mRNA decay (NMD) machinery, as a critical regulator of the liver development^18^. Thus, although specific RBPs have been studied for their role in mediating developmental processes of liver, no global association analysis has been performed in humans to uncover the repertoire of RBPs contributing to changes in liver functions with age and gender. Hence, to complement this gap in our global understanding about the functions of RBPs as critical regulators in liver, in this study a genome-wide association analysis of their expression patterns with age and gender was conducted. To achieve this, we performed an association analysis of RBPs expression levels in human liver tissues with respect to age and gender, by integrating a dataset of 1344 genes experimentally known to encode for RBPs. This allowed us to catalogue for the first time age and gender related associations for RBPs as well as to study the wiring patterns of RBPs and their protein complexes (protein-protein interaction networks) with liver maturation in the human genome.

## Results

### RBPs exhibit significant alterations in their expression with aging human liver

**Figure 1:**
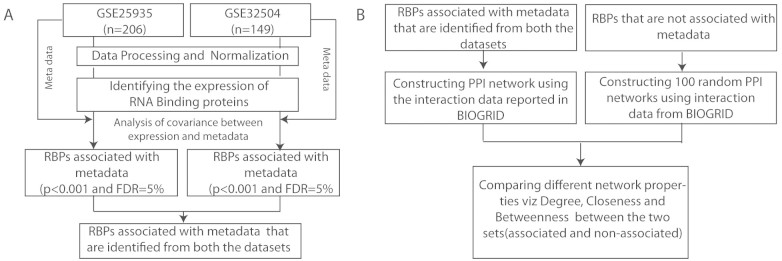
Flowcharts summarizing the major steps involved in association and network analysis. (A) Flowchart showing the various steps adopted for pre-processing and normalization of raw microarray data from both the liver cohorts followed by analysis of covariance between expression and metadata (age and gender) using functions in R statistical framework to identify the most significant associations. (B) Flowchart showing the steps for construction of protein-protein interaction (PPI) networks between RBPs associated with metadata (either age or gender) and a control set of 100 randomized PPI networks from RBPs not associated with metadata followed by the comparison of network properties between them.

**Figure 2:**
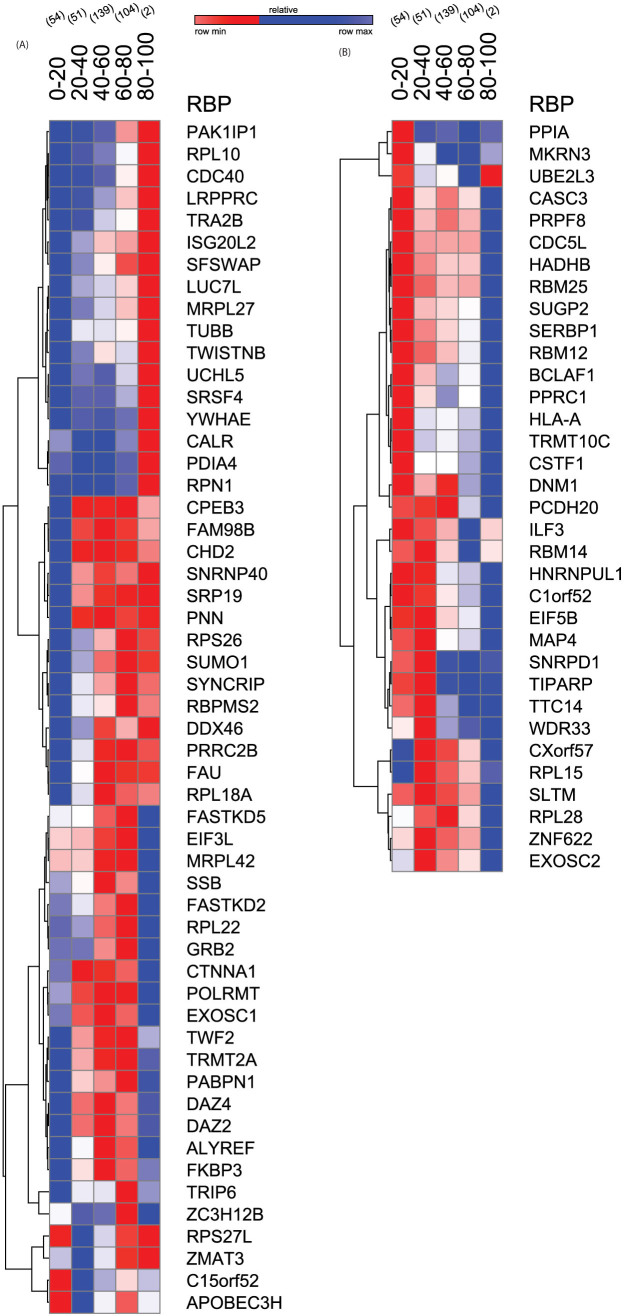
Expression patterns of RBPs associated with age. (A) Heat map showing RBPs that are positively correlated with age (p < 0.001, FDR < = 5%) from both the cohorts in the study (B) Heat map showing the list of RBPs that are negatively correlated with age (p < 0.001, FDR < = 5%). Age ranges are shown from 0 to 100 years and expression levels are binned into 20 year intervals with the number of samples comprising of 54, 51, 139, 104 and 2 in each of these respective bins.

Association and correlation analysis revealed the expression of 88 RBPs to be significantly varying with age in human liver tissues (p < 0.001, FDR = 5%) (Figure 1, Materials and Methods, Supplementary Tables 1 and 2). These included 54 RBPs whose expression was observed to be increasing with aging liver (Figure 2A). Among these, LRPPRC – a leucine rich RNA Binding protein associated with post splicing nuclear RNP complexes and predominantly located within mitochondria^19^ was found to have the highest correlation. Following this is SUMO1- a qualified RBP identified from the interactome capture of the human HeLa cell lines^20^. In addition to these, we also capture one of the genes in the p53 developmental pathway – ZMAT3, a zinc finger protein involved in the mRNA stability whose overexpression suppresses tumour growth^21^,^22^. Clustering the expression levels of these significantly increasing age-associated RBPs resulted in the formation of several subgroups with three prominent patterns emerging from these clusters. These include a) RBPs which are less expressed in young and middle age but higher in old (e.g. PAK1IP1, SRSF4), b) RBPs which are less expressed in young and higher in middle and old (e.g. CPEB3, SUMO1), c) RBPs with higher expression in middle but less in young and old (e.g. MRPL42, PABPN1). It is possible to speculate that the members of these clusters/subgroups of RBPs might have similar or co-operative functions in gene regulation at specified time points. On the other hand, we also found 34 genes to be significantly associated but negatively correlated with age (Figure 2B). For instance, we find PCDH20 – a novel member of the protocadherin gene family to be significantly negatively correlated with age. Moreover, an independent study has recently identified that the mRNA levels of this proteins to be down-regulated in the hepatocellular carcinomas when compared to the disease free livers and thus functioning as a tumour suppressor^23^. Another notable example is PPIA – member of the peptidyl-prolyl cis-trans isomerase family known to accelerate the folding of proteins which in our analysis, was seen to negatively correlate with age. Additionally, a recent study proposed that stable expression of PPIA (or CypA) in liver cells confers resistance to anticancer drugs like doxorubicin and vincistrine^24^. A hierarchical clustering of these 34 RBPs which decreased in expression with age resulted in the formation of three major subgroups - a) RBPs with higher expression in young but low in middle and old (e.g. RBM25, CSTF1), b) RBPs which are higher expressed in young and middle but less in old (e.g. ILF3, EIF5B), c) RBPs with low expression in young and old but high in middle (e.g. RPL28, EXOSC2). In summary, we found 54 RBPs (61.4%) to be positively correlated with age suggesting that the majority of the age-associated post-transcriptional control in liver is due to increased expression of RBPs indicating a potential for increased number of targets with age^9^.

To know if the repertoire of proteins important in aging liver is enriched for RBPs, we performed a similar association analysis for TFs and Non-RBPs (See Materials and Methods). This indicated that a higher proportion of RBPs are seen to be significantly associated (~6.5%) when compared to the TFs (~5.6%) and Non-RBPs (~5.4%) (p = 0.09, Fisher's exact test). This strengthens the notion that RBPs are at least as important as other regulatory molecules implicated in aging livers (See Supplementary Table 3 for the list of TFs and Non-RBPs associated with age).

### Several RBPs showed similar changes in expression within aging mouse and rat liver tissue

**Figure 3:**
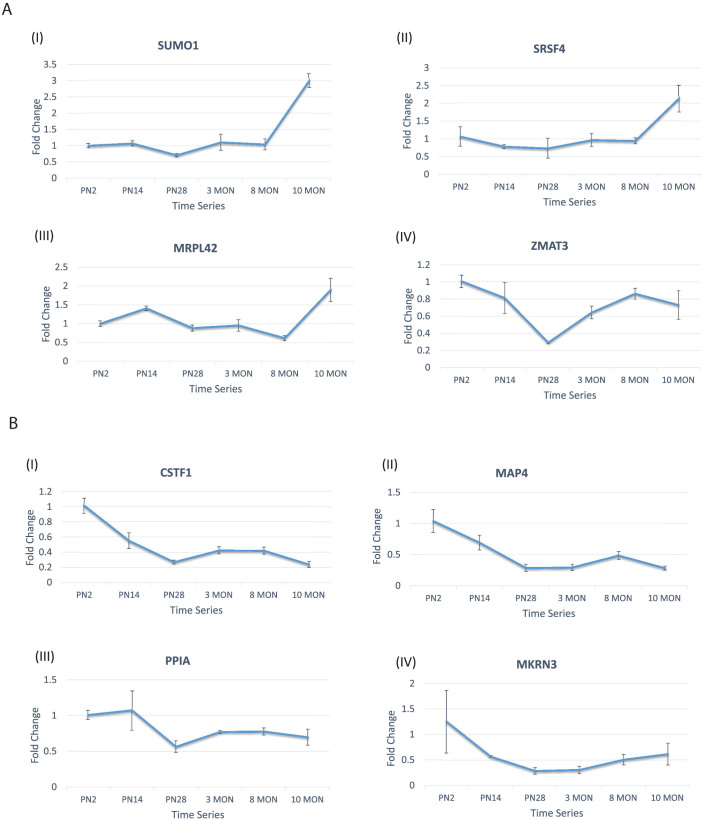
Expression levels measured using RT-PCR for eight RBPs in mouse for the time series ranging from postnatal 3 days to 10 months. X-axis shows the age of the mouse and y-axis represents the average fold change in expression with standard error of mean shown for each time point. (A) shows four RBPs tested in mouse model to see if the expression is positively correlated with age (B) shows four RBPs tested in the mouse model which were found to be negatively correlated with age in the human samples. All the tested RBPs show strong correlation with age in the respective directions in human through our computational pipeline.

To validate whether our observations in the human liver samples are reproducible, we measured the relative fold change of the RBPs which showed strong positive correlation with age, using real-time PCR in mouse liver for four different RBPs (see Materials and Methods) (Figure 3A). In general, RBPs which showed increasing expression with age in human liver samples also showed a very similar trend in mouse liver. As shown in Figure 3A, this trend is observed for Sumo1, Srsf4 and Mrpl42 which show a late and immediate increase in expression (> two-fold) at 10 months (Supplementary Table 4).

Similar analysis was performed on four RBPs, which exhibited a strong negative correlation between expression and age, in the mouse liver (Figure 3B). Of the four RBPs validated, Cstf1 and Map4 exhibited strong decrease in expression levels with aging mice. Ppia and Mkrn3 followed decreasing trends in expression levels albeit weakly (Supplementary Table 4).

To further validate our findings, we extracted the expression profiles of these RBPs in rat livers (See Materials and Methods). We observed a strong reproducibility in the expression patterns of 33 human RBPs in the aging rat tissues (See Supplementary Figure 2). For example, we observe SUMO1, SYNCRIP and PDIA4 to be positively correlated while HADHB and PPRC were found to be negatively correlated with age in the rat livers. More generally, to study the enrichment of RBPs changing with age in human liver to be reproducible in the rat liver, we compared the proportions of the respective trends of the RBPs. RBPs increasing with age in human were found to be significantly enriched to be also detected as positively correlated with age in rat (p = 0, Hypergeometric probability). We also found the RBPs decreasing with age in humans to be markedly over-represented to be negatively correlated with age in rat samples (p < 3.3e-13, Hypergeometric probability) suggesting that the observations are reproducible in mouse and rat genomes despite the variations in the samples and the absence of orthologs for several RBPs due to evolutionary divergence between the species.

### A small fraction of the RBPs are sexually dimorphic in humans

**Figure 4:**
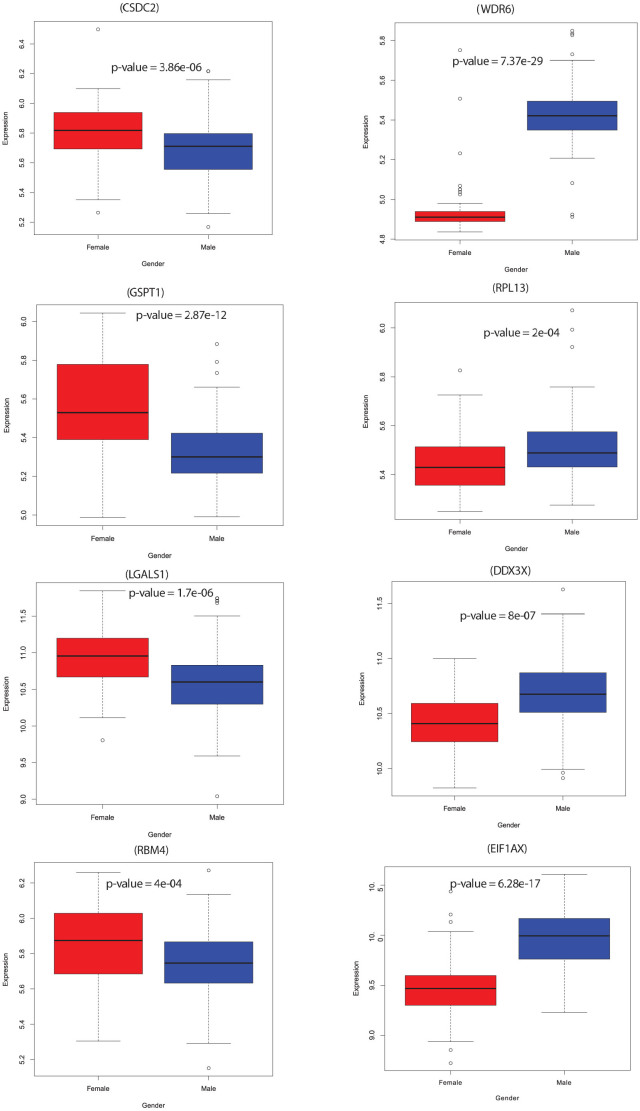
Boxplots showing expression levels of selected set of RBPs which were found to be gender specific and thus are differentially expressed in male vs female samples in humans. All the comparisons are significant at p-value < = 0.05 (Wilcox test).

Association analysis also enabled us to identify sex-specific RBP expression and to uncover variations between male and female samples (see Materials and Methods). Briefly, this analysis revealed 45 RBPs whose expression was found to have a significant difference between genders (p < 0.001, FDR = 5%) (Supplementary Tables 1 and 2). We also found that a small set of these gender-associated RBPs (~40%) are higher expressed in males when compared to females. Several RBPs including WDR6, RBM4, GSPT1 and EIF1AX were found to be significantly differentially expressed between male and female samples (Figure 4). Of these, WDR6, member of the WD repeat protein family, implicated in the cell growth arrest was observed to be expressed at relatively higher levels in males. WD repeats are minimally conserved regions of around 40 amino acids generally bracketed by gly-his and trp-asp, which may further facilitate multi-protein complex formation. It is known to enhance STK11/LKB1- induced cell growth suppression activity, a negative regulator of amino acid starvation–induced autophagy^25^,^26^. Similarly, EIF1AX-eukaryotic translation initiation factor was observed to be expressed in higher levels in males than in females. In contrast, RBPs like GSTP1, LGAS1 and RBM4 were found to exhibit higher expression in females compared to males (Figure 4). Overall, our results suggest that a relatively smaller fraction of the RBPs are associated with gender compared to age in human liver and majority of these are up-regulated in females compared to males.

### Age-associated RBPs form a dense modular network of protein interactions

**Figure 5:**
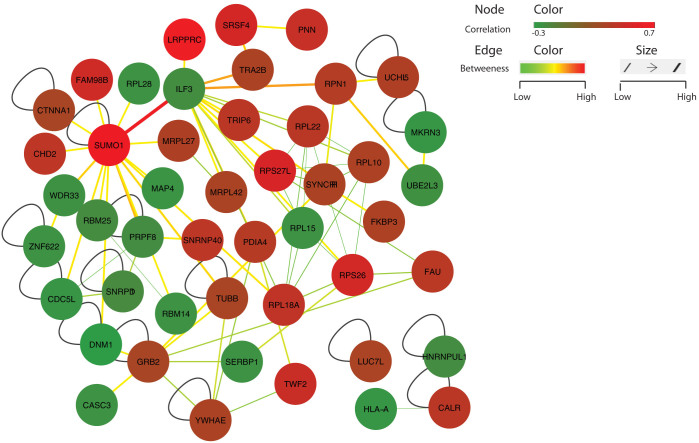
Protein interaction network of RBPs associated with age. Network showing the experimentally verified protein-protein interactions from the BioGRID database^28^ between age-associated RBPs. Node colours vary between green and red signifying the extent of correlation of expression with age, with positively correlated RBPs coloured in red. Similarly, edge thickness and colour are highlighted with edge betweenness scores. Edges with higher edge betweenness centrality scores are shown in a thick red colour. Network also highlights how RBPs positively and negatively correlated with age, interact among themselves to form dense modular RNP complexes.

RBPs attain their precise spatio-temporal control of gene expression most typically by forming protein complexes in the cell^27^. Therefore, to understand how the 88 RBPs, whose expression was found to be strongly associated with age in human samples, are interacting, we constructed a network of experimentally known protein-protein interactions (PPIs) between them. This was achieved using documented interactions between RBPs from the BioGRID database^28^ (Materials and Methods). This resulted in a network of 86 protein-protein interactions between the age-associated RBPs (Figure 5). A closer inspection of the distribution of clustering coefficients of the nodes, which is a proxy for the modularity of the network, indicated that the network is significantly modular compared to random networks of the same size (Materials and Methods) (Supplementary Figure 1). Indeed, we found that the age-associated RBP network exhibited twice the clustering coefficient than random networks (0.27 vs 0.14 median clustering coefficient, p = 2.2e-146, Wilcox test). Additional analysis to cluster the network into likely protein complexes indicated the presence of two high confidence protein complexes using ClusterONE^29^ (Supplementary Table 6). One of these complexes comprised of ILF3, RPS26, FAU, SYNCRIP- a member of the cellular heterogeneous nuclear ribonucleoprotein (hnRNP) family and several members of the 60S ribosomal subunit. This dense network also comprised of SUMO1 and ILF3, forming hubs with highest number of interactions (Figure 5). Figure 5 also shows the edge betweenness (normalized) defined as the number of shortest paths going through an edge of interest and is analogous to the node betweenness, a centrality measure for nodes in a network (see Materials and Methods). We found a high edge betweenness score for the edge connecting SUMO1-protein whose expression increases with age and ILF3-protein whose expression decreases with age suggesting that there might be an inverse relationship in their stoichiometry with age to separate the age-related RNP complexes/network into different partitions.

### Age and gender-specific associations in liver show significantly higher network centrality measures

**Figure 6:**
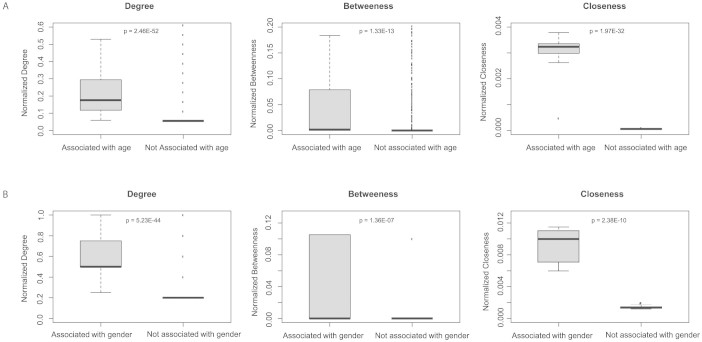
Boxplots for comparison of various network centrality measures (A) between the networks of RBPs associated with age and not associated with age. The network of RBPs not associated with age comprises of an ensemble of 100 random networks constructed based on the randomization approach discussed in Materials and Methods section. Boxplots correspond to the comparison of normalized degree, betweenness and closeness values in the respective networks. All normalizations are based on the maximum value observed for the particular centrality measure in the corresponding network/s. (B) between the networks of RBPs associated with gender and not associated with gender. The network of RBPs not associated with gender comprises of an ensemble of 100 random networks constructed based on the randomization approach discussed in Materials and Methods section. Boxplots correspond to the comparison of normalized degree, betweenness and closeness values in the respective networks. All normalizations are based on the maximum value observed for the particular centrality measure in the corresponding network/s.

The importance of a protein can be assessed by measuring the centrality of a node in the PPI network. Centrality of a node can be measured by a number of different metrics including the most commonly used measures - degree, closeness and betweenness^30^. Therefore, to assess the significance of the centrality of the nodes in the age-associated RBP network, we compared the various centrality measures with that observed in a control set of random networks that contains a network of interactions among proteins that are not associated with age (see Materials and Methods). Normalized degree of the nodes in the network of proteins associated with age was found to be significantly higher than that seen in the control set (p = 2.46e-52, Wilcox test) suggesting a higher connectivity among the age-associated RBPs (Figure 6A). Similarly, the distribution of closeness values was observed to be significantly different (p = 1.97e-31, Wilcox test) and higher than the control set suggesting the existence of a denser network of interactions among proteins associated with age. The distribution of betweenness centrality scores were also found to be significantly different (p = 1.33e-13, Wilcox test) and higher than the control set suggesting that these proteins are likely to play essential roles. Our results also suggest that these genes encoding for RBPs and strongly associated with age might form a dense and intertwined network of protein complexes contributing to the regulation of several age-related post-transcriptional processes in the liver tissue such as development, growth and regeneration. Similar results were observed when we compared network of RBPs which expressed differentially in male vs female samples to the set of random networks of RBPs which are not gender specific (Figure 6B). In particular, we found that each of the centrality measures- degree, betweenness and closeness were significantly higher for gender-associated RBPs compared to that seen in the random networks.

## Discussion

Many studies to date have focused on genes that change with age and gender but the regulatory molecules mediating such mechanisms remain unclear. Previous studies have shown that eQTLs (expression Quantitative Trait Loci) interact with age and gender and play a major role in disease susceptibility^4^,^31^. However, the contributing regulatory factors remain unclear. For instance, Masuda et. al, show that mRNA turnover and translation regulatory (TTR) RBPs show similar pattern in many tissues like gastrointestinal, urinary and immune systems in an age dependent manner^32^. This article emphasises that change in expression of a specific group of RBPs - important players in mRNA turnover and/or translation, as a function of age in human liver samples is an important but poorly studied level of association. However, post-transcriptional control is mediated by several hundreds of RBPs in the human genome and our knowledge about their role in controlling age-associated processes is rather limited. Therefore, in this study, to address this problem, we have compiled two different liver expression cohorts and performed a global association analysis between hundreds of experimentally characterized RBPs in the human genome for the first time to uncover the compendium of RBPs varying with age and gender. We observe several RBPs to be changing in expression with age and gender suggesting an important role for post transcriptional regulation during liver development, maturation and regeneration. Majority (~60%) of the age-associated RBPs were found to be increasing in their expression levels with age while a small set of gender-associated RBPs (~40%) were found to be up-regulated in males. Several of these predicted associations were confirmed in the rat tissues and experimentally validated in a mouse model. This resulted in a significant number of human RBPs showing similar expression patterns in aging liver of rat and mouse, thus corroborating our findings. Further analysis of the protein interaction network of RBPs associated with age and gender based on the centrality measures like degree, betweenness and closeness revealed that several of these RBPs might be prominent players in aging livers and impart gender-specific alterations in gene expression via the formation of protein complexes. Furthermore, SUMO1 – gene which was positively correlated with age was observed to be sharing a highest edge betweenness with ILF3 that was negatively correlated with age signifying a possible existence of a co-regulatory mechanism linking sumoylation and translational regulation – processes controlled by these respective proteins. This also suggests that the interaction between SUMO1 and ILF3 might facilitate a link between post transcriptional control, protein stability, nuclear cytosolic transport of proteins and translational control. It is worth mentioning that in this study we are only looking at a static network of physical interactions between RBPs associated with age (Figure 5). However, it is easy to note that this dynamic network might evolve/vary with age suggesting that varying sub-networks might be active depending on the developmental time frame and interplay with other cellular processes. So it is possible to speculate that there may be a time point in dynamics when the stoichiometry of the physical interactions between RBPs increasing and decreasing with age may disrupt leading to causative effects or disease phenotypes. With increasing high resolution data from next generation sequencing and proteomics pipelines from hundreds of individuals it should be possible in the near future to understand such complex and rich set of associations between genes and proteins across cell types. Hence, this study will not only help us gain insight into the role of post-transcriptional regulatory molecules in aging and gender specific expression of genes but also provide a foundation for identifying the causative players contributing to the splicing eQTLs.

## Methods

### Data set of RNA-binding proteins and expression profiles for human liver tissues

In the present study, we catalogued a set of 1344 genes encoding for RBPs in the human genome. This compendium comprised of proteins identified as RBPs in several recent experimental screens, including Castello et. al^33^, Baltz et. al.^34^, Ray et. al^35^, human orthologs of RBPs identified in mouse embryonic stem cells by Kwon et. al^36^, and those reported in RBPDB^37^.

In this study, we employed two different microarray-based liver expression cohorts for performing the association analysis. The first, a study by Innocenti et. al, which profiled 206 normal human livers (183 European Americans and 23 African Americans) of which 74 were females and 132 males with age ranging from 1 to 81 years to map expression quantitative trait loci through genome wide association mapping (GSE25935)^38^. The second study by Schroder et. al, profiled 149 liver samples of Caucasian origin to identify expression quantitative loci (eQTL) in human liver of 71 females and 78 males ranging from 7 to 85 years of age (GSE32504)^39^. Data processing and normalization were carried out using the packages available in the R statistical framework. Raw expression data from GSE25935, generated using Agilent array was available in single colour format. This raw data was processed using limma package. For normalization we used a three step approach 1) for background correction (method = “normexp” and offset = 50) 2) for normalization between arrays (method = “quantile”) and finally 3) log transformation of the normalized data was performed. Secondly, in case of GSE32504, raw data was generated using Illumina Bead Studio version 3 and thus beadarray^40^ and lumi^41^ packages from Bioconductor biocLite were used for processing of the raw data. Normalization of the raw data was carried out in a single step using quantile normalization approach. Figure 1A shows a generic flowchart summarizing the various steps involved in the data processing and analysis of the two microarray datasets.

### Associating the expression of RBPs with age and gender

To test the association of expression with age and gender, we modelled the expression of each RBP as a dependent variable with age and gender as independent variables. Using the analysis of covariance -a statistical test used to explain the variance between independent and dependent variables, available from the R statistical framework we computed the significance of association. For each of the two microarray datasets, normalized expression of RBPs was simultaneously associated with the metadata - age and gender (Figure 1). Associations predicted at p < 0.001 (FDR = 5%, Benjamini-Hochberg^42^ procedure) were considered significant. Thus, RBPs that were identified to significantly associate with age from either of the datasets were merged to form a non-redundant set. Similarly, a non-redundant set of RBPs associated with gender was obtained from the two datasets.

Further, correlation analysis was performed between RBPs expression and age for the two datasets separately. Spearman correlation was calculated and p-value for each correlation association (expression vs age) was calculated. Associations identified at p < 0.001 (FDR = 5%, Benjamini-Hochberg procedure) were considered significant. A similar approach was adopted to identify the associations of 1348 transcription factors extracted from DBD: Transcription factor prediction database^43^ and ~18,000 Non-RBPs in the human genome.

### Network dynamics of RBPs associated with age and gender

We categorized the 1344 RBPs into two groups – those that are significantly associated with age (p < 0.001 & FDR = 5%) in either of the two liver cohorts and the remaining genes that are not associated with age. A protein-protein interaction (PPI) network was constructed for each of the two groups of RBPs, using the publicly available experimentally verified interaction data from BioGRID database^28^. Age-associated RBP interaction network comprised of 47 nodes and 86 edges while the non-age associated RBP interaction network comprised of 1112 nodes and 9325 edges. Since the non-age associated network was significantly larger in terms of the number of nodes and edges, we employed a custom randomization model to generate random networks of the same size as the observed age-associated RBP network. In other words, the interaction network of proteins that are not associated with age was used to create 100 random networks each comprising of the same number of edges to serve as a control set. Briefly, let there be x number of edges in the network where RBPs are associated with age and y edges in the network not associated with age (assuming y > x). We obtained 100 random networks to represent control networks not associated with age and with each iteration we randomly obtained x edges and thus constructed 100 random networks with RBPs not associated with age interacting among themselves. The same protocol was applied to generate interaction networks for genes/proteins associated and non-associated with gender. Non-gender RBP interactions were then used to construct 100 random networks of RBPs not associated with gender.

To study the properties of the PPI networks associated with age and gender, we used igraph, a publicly available R package for analysing graphs [see http://cneurocvs.rmki.kfki.hu/igraph/and http://www.r-project.org]. In particular, since the network of PPIs analysed in this study is undirected, we used the corresponding versions of the functions: degree, clustering coefficient (transitivity), betweenness and closeness for calculating the degree, clustering coefficient, betweenness and closeness centralities of a node. Betweenness centrality, which is the number of shortest paths going through a node was calculated using the brandes algorithm^44^ implemented in R. Similarly, closeness, measured as the inverse of the average length of the shortest paths to all the other nodes in the graph, was obtained using the implementation in R. Since the centrality measures, betweenness and closeness use the shortest path lengths between all pairs of nodes in a graph, for cases where no path exists between a particular pair of nodes, shortest path length was taken as one less than the maximum number of nodes in the graph. Note that this is also the default assumption for calculating centrality measures in igraph. All the network centrality measures were compared between age-associated and non-age-associated random RBP networks to study for differences in the distributions by performing non-parametric tests. Similarly, gender-associated RBPs were compared for their corresponding network properties in non-associated random networks.

### Validation of the RBP associations in liver tissues of mouse and rat

#### Mouse

Liver tissues were collected from mice at the indicated time points for mRNA expression analysis. For further details regarding mice strain and age refer to Supplementary Table 5. Total RNA was isolated from mouse liver tissue using TRIzol. Total RNA (~5 μg) was reverse transcribed using Random Hexamer Primer (Thermo) and the Thermo Scientific Maxima Reverse Transcriptase kit. Quantitative real-time PCR were performed in duplicates using 130 ng of cDNA per reaction on an Eco Real-Time PCR system using Quanta PerfeCTa SYBR Green FastMix. An initial activation step for 10 min at 95°C was followed by 40 cycles of 95°C for 10 s and 60°C for 30 s. Primer sequences used to measure relative steady-state expression of mRNA are provided in Supplementary Table 5. Fold change of the mRNA was calculated as previously described^45^. Data is presented as mean ± SEM.

We followed the NIH Guide for the Use and Care of Laboratory Animals. The Institutional Animal Care and Use Committees of Universities of Illinois approved all experiments. All the methods were carried out in accordance with the approved guidelines.

#### Rat

The orthologs of 88 human RBPs associated with age in rat were extracted from the ENSEMBL compara, resulting in 65 corresponding genes. Expression profiles of these orthologs in 8 rat liver tissues RNA-sequenced at four stages – 2 weeks, 6 weeks, 21 weeks and 104 weeks were extracted from the Rat Body Map Database (http://pgx.fudan.edu.cn/ratbodymap/). This data was then used to the compare the expression patterns of RBPs between human and rat livers to identify the genes which followed the same trend as in humans. Heat maps were generated for RBPs which increased or decreased in both humans and rats (Supplementary Figure 2).

## Author Contributions

P.C. and Y.N. carried out the computational analysis of the study and wrote parts of the manuscript. W.A. collected the mouse liver tissues from different ages, performed qRT-PCR against different genes of interest, analysed data, and wrote parts of the manuscript. A.K. and S.C.J. supervised and designed research, analysed data, and wrote parts of the manuscript.

## Supplementary Material

Supplementary Information Supplementary information
