# Supplementary material for: Uncovering RNA binding proteins associated with age and gender during liver maturation
Source: Sci Rep. 2015 Mar 31;5:9512. doi: 10.1038/srep09512 (PMC4379467; doi:10.1038/srep09512)
Supplement: Supplementary Information [file srep09512-s1.pdf]

Supplementary Material for

Uncovering RNA binding proteins associated with age and gender during liver maturation

Praneet Chaturvedi[1], Yaseswini Neelamraju[1], Waqar Arif[2], Auinash Kalsotra[2], Sarath Chandra Janga[1, 3, 4,*]

[1]Department of BioHealth Informatics, School of Informatics and Computing, Indiana University Purdue University, 719 Indiana Ave Ste 319, Walker Plaza Building, Indianapolis, Indiana 46202

[2]Departments of Biochemistry and Medical Biochemistry, University of Illinois, Urbana-Champaign, Illinois 61801, USA

[3]Centre for Computational Biology and Bioinformatics, Indiana University School of Medicine, 5021 Health Information and Translational Sciences (HITS), 410 West 10th Street, Indianapolis, Indiana, 46202

[4]Department of Medical and Molecular Genetics, Indiana University School of Medicine, Medical Research and Library Building, 975 West Walnut Street, Indianapolis, Indiana, 46202

[*]*Correspondence should be addressed to :*

Sarath Chandra Janga (scjanga@iupui.edu)

719 Indiana Avenue Ste 319 , Walker Plaza Building
Indianapolis , Indiana - 46202
Tel: +1-317-278-4147, Fax: +1-317-278-9201

Legends

Supplementary Figure 1: Boxplot showing the comparison of clustering coefficients. Comparison of Clustering coefficient of RBPs associated with age and RBPs not associated with age.

Supplementary Figure 2: Expression patterns of rat orthologs of age-associated human RBPs which increased or decreased with age in both humans and rats. A) Heat map showing the expression patterns of rat orthologs of RBPs which were found to be positively correlated with aging human liver B) Heat map showing the expression patterns of rat orthologs of RBPs which were found to be negatively correlated with aging human liver.

Supplementary Table 1: Table showing the age and gender associations of RBPs in Innocenti et. al. All the associations are derived using ANOVA test from Innocenti et. al (GSE25935). Correlation of expression of RBPs with age is calculated using spearman correlation.

Supplementary Table 2: Table showing the age and gender associations of RBPs in Schroder et. al. All the associations are derived using ANOVA test from Schroder et. al (GSE32504). Correlation of expression of RBPs with age is calculated using spearman correlation.

Supplementary Table 3: Table showing the list of TFs and NonRBPs associated with age.

Supplementary Table 4: Tables showing the raw data from real time PCR experiments of selected RBPs.

Supplementary Table 5: Provides information for mouse used in this study and the information on the primers for the genes whose expression is measured with age in mouse.

Supplementary Table 6: ClusterONE results on two significant sub-clusters from age associated network of RBPs.

Supplementary Figure 1

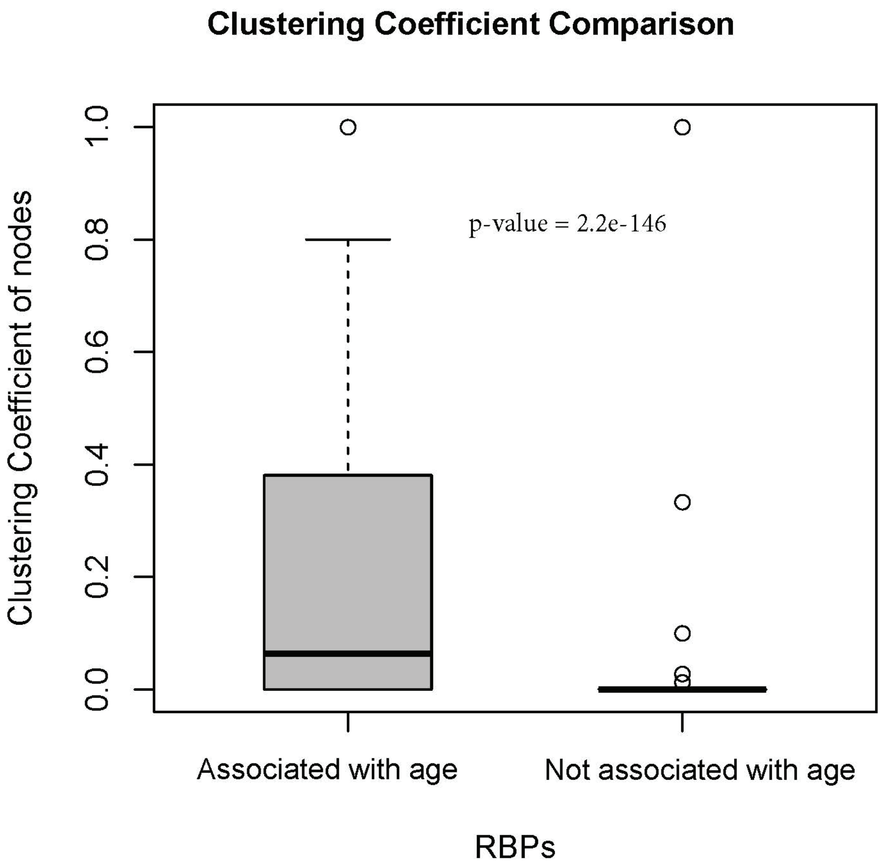

Supplementary Figure 2

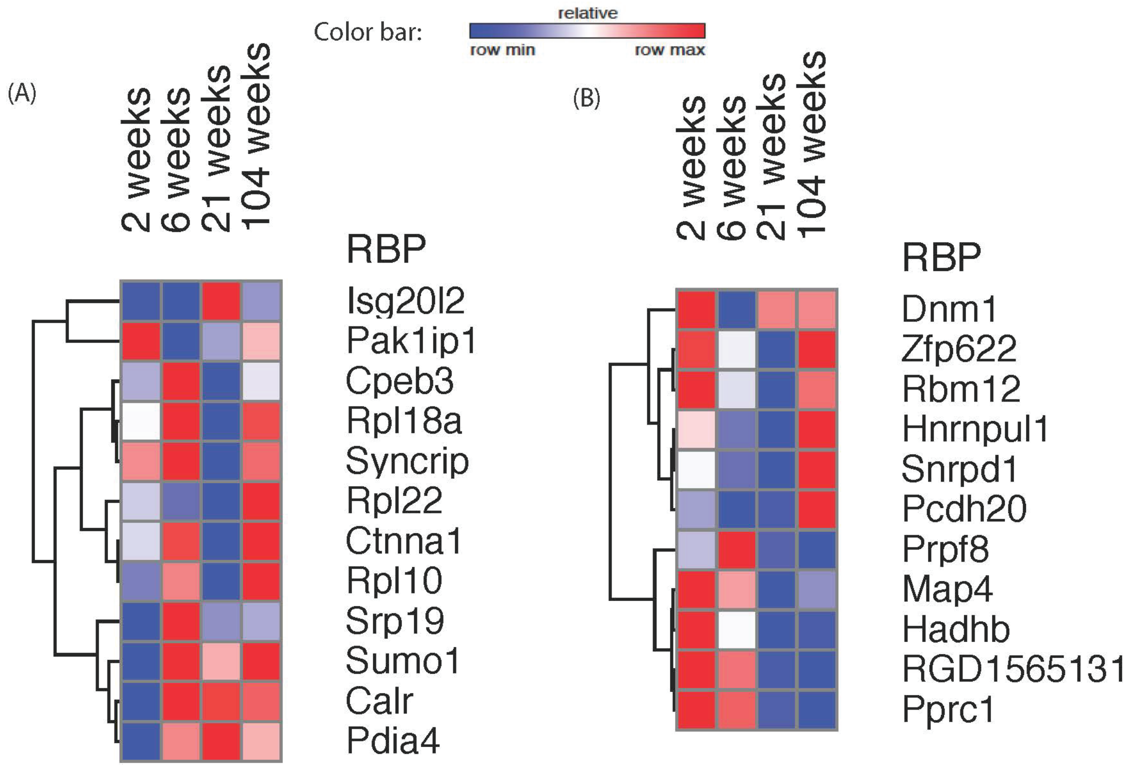

Supplementary Table 1

RBP<Probe_ID>	ENSEMBL_GENE_ID	GENE_DESCRIPTION	ENSEMBL_MOUSE_GEN	AGE(ANOVA_P-VALUE)	GENDER(ANOVA_P-VALUE)	CORRELATION_WITH_AGE	P-VALUE_OF_CORRELATIO	Benjamini & Hochberg_Corrected_correlation_p-valu
LRPPRC<23425>	ENSG00000138095	leucine-rich pentatricopeptide repeat containing	ENSMUSG00000024120	3.22E-16	NS	0.660551554	3.38E-27	2.09E-24
SUMO1<5315>	ENSG00000116030	small ubiquitin-like modifier 1	ENSMUSG00000026021	2.18E-17	NS	0.623312977	1.44E-23	4.44E-21
SRP19<15524>	ENSG00000153037	signal recognition particle 19kDa	ENSMUSG00000014504	8.06E-19	NS	0.541602159	4.25E-17	8.74E-15
SRSF4<9245>	ENSG00000116350	serine/arginine-rich splicing factor 4	ENSMUSG00000028911	2.71E-11	NS	0.442706155	2.69E-11	4.08E-09
RPS26<37250>	ENSG00000197728	40S ribosomal protein S26-like	ENSMUSG00000059775	8.54E-10	NS	0.440881434	3.31E-11	4.08E-09
RPS26<37250>	ENSG00000197728	ribosomal protein S26	ENSMUSG00000059775	8.54E-10	NS	0.440881434	3.31E-11	4.08E-09
PAK1IP1<2397>	ENSG00000111845	PAK1 interacting protein 1	ENSMUSG00000038683	1.08E-10	NS	0.436640916	5.34E-11	5.49E-09
FAM98B<25947>	ENSG00000171262	family with sequence similarity 98, member B	ENSMUSG00000027349	8.10E-08	NS	0.420913171	2.98E-10	2.63E-08
TWF2<36669>	ENSG00000247596	twinfilin actin-binding protein 2	ENSMUSG00000023277	7.43E-06	NS	0.393514904	4.87E-09	3.76E-07
SFSWAP<13213>	ENSG00000061936	splicing factor, suppressor of white-apricot homolog (Drosophila)	ENSMUSG00000029439	1.54E-06	2.87E-05	0.382959946	1.34E-08	8.39E-07
PNN<10461>	ENSG00000100941	pinin, desmosome associated protein	ENSMUSG00000020994	4.18E-08	NS	0.382789007	1.36E-08	8.39E-07
CDC40<35459>	ENSG00000168438	cell division cycle 40	ENSMUSG00000038446	0.000895095	NS	0.376223309	2.50E-08	1.40E-06
TRMT2A<16907>	ENSG00000099899	tRNA methyltransferase 2 homolog A (S. cerevisiae)	ENSMUSG00000022721	1.19E-07	0.000444792	0.352192331	2.09E-07	1.02E-05
PABPN1<11696>	ENSG00000100836	poly(A) binding protein, nuclear 1	ENSMUSG00000092232	0.003953619	NS	0.351898508	2.14E-07	1.02E-05
FASTKD5<34644>	ENSG00000215251	FAST kinase domains 5	ENSMUSG00000079043	2.33E-05	NS	0.34872344	2.79E-07	1.16E-05
PPIA<12107>	ENSG00000196262	peptidylprolyl isomerase A (cyclophilin A)	ENSMUSG00000055795	7.72E-11	NS	-0.348632135	2.82E-07	1.16E-05
PPIA<12107>	ENSG00000196262	peptidyl-prolyl cis-trans isomerase A-like	ENSMUSG00000055795	7.72E-11	NS	-0.348632135	2.82E-07	1.16E-05
RPL18A<10915>	ENSG00000105640	small nucleolar RNA, H/ACA box 68	ENSMUSG00000045128	0.000110171	0.013932744	0.347344945	3.13E-07	1.21E-05
RPL18A<10915>	ENSG00000105640	ribosomal protein L18a	ENSMUSG00000045128	0.000110171	0.013932744	0.347344945	3.13E-07	1.21E-05
CHD2<41716>	ENSG00000173575	chromodomain helicase DNA binding protein 2	ENSMUSG00000078671	2.07E-05	NS	0.343606946	4.27E-07	1.54E-05
CPEB3<17417>	ENSG00000107864	cytoplasmic polyadenylation element binding protein 3	ENSMUSG00000039652	1.34E-05	NS	0.342994586	4.49E-07	1.54E-05
CSTF1<18870>	ENSG00000101138	cleavage stimulation factor, 3' pre-RNA, subunit 1, 50kDa	ENSMUSG00000027498	0.000247602	NS	-0.338071686	6.70E-07	2.18E-05
SNRNP40<27970>	ENSG00000060688	small nuclear ribonucleoprotein 40kDa (U5)	ENSMUSG00000074088	0.000540674	NS	0.336034836	7.89E-07	2.43E-05
ISG20L2<41756>	ENSG00000143319	interferon stimulated exonuclease gene 20kDa-like 2	ENSMUSG00000048039	2.40E-06	NS	0.328111238	1.48E-06	4.35E-05
TIPARP<29972>	ENSG00000163659	TCDD-inducible poly(ADP-ribose) polymerase	ENSMUSG00000034640	1.23E-06	NS	-0.319910294	2.77E-06	7.77E-05
RPL22<39782>	ENSG00000116251	ribosomal protein L22	ENSMUSG00000028936	0.000167255	NS	0.319241641	2.91E-06	7.81E-05
FAU<20630>	ENSG00000149806	Finkel-Biskis-Reilly murine sarcoma virus (FBR-MuSV) ubiquitously expressed	ENSMUSG00000050299	2.09E-06	NS	0.310514837	5.56E-06	0.000138455
TRMT10C<35291>	ENSG00000174173	tRNA methyltransferase 10 homolog C (S. cerevisiae)	ENSMUSG00000044763	7.98E-05	NS	-0.310391267	5.61E-06	0.000138455
MKRN3<21516>	ENSG00000179455	makorin ring finger protein 3	ENSMUSG00000070527	8.78E-07	NS	-0.308885083	6.26E-06	0.000143281
TWISTNB<34734>	ENSG00000105849	TWIST neighbor	ENSMUSG00000020561	4.28E-06	NS	0.308860369	6.27E-06	0.000143281
HLA-A<4147>	ENSG00000223980	major histocompatibility complex, class I, A	NA	0.000100789	NS	-0.307987139	6.68E-06	0.000147199
HLA-A<4147>	ENSG00000235657	major histocompatibility complex, class I, A	NA	0.000100789	NS	-0.307987139	6.68E-06	0.000147199
HLA-A<4147>	ENSG00000229215	major histocompatibility complex, class I, A	NA	0.000100789	NS	-0.307987139	6.68E-06	0.000147199
HLA-A<4147>	ENSG00000227715	major histocompatibility complex, class I, A	NA	0.000100789	NS	-0.307987139	6.68E-06	0.000147199
HLA-A<4147>	ENSG00000231834	major histocompatibility complex, class I, A	NA	0.000100789	NS	-0.307987139	6.68E-06	0.000147199
HLA-A<4147>	ENSG00000224320	major histocompatibility complex, class I, A	NA	0.000100789	NS	-0.307987139	6.68E-06	0.000147199
HLA-A<4147>	ENSG00000206505	major histocompatibility complex, class I, A	NA	0.000100789	NS	-0.307987139	6.68E-06	0.000147199
HLA-A<4147>	ENSG00000206503	major histocompatibility complex, class I, A	NA	0.000100789	NS	-0.307987139	6.68E-06	0.000147199
CDC5L<39963>	ENSG00000096401	cell division cycle 5-like	ENSMUSG00000023932	7.07E-06	NS	-0.297885959	1.37E-05	0.000291479
DDX46<29863>	ENSG00000145833	DEAD (Asp-Glu-Ala-Asp) box polypeptide 46	ENSMUSG00000021500	6.54E-05	NS	0.293806768	1.82E-05	0.000374313
FASTKD2<28799>	ENSG00000118246	FAST kinase domains 2	ENSMUSG00000025962	1.05E-05	NS	0.28974268	2.40E-05	0.000477677
ALYREF<6863>	ENSG00000183684	Aly/REF export factor	ENSMUSG00000025134	1.24E-06	NS	0.286733059	2.94E-05	0.000566869
CHD2<12090>	ENSG00000173575	chromodomain helicase DNA binding protein 2	ENSMUSG00000078671	0.000171363	NS	0.282449977	3.90E-05	0.000729182
PRRC2B<34830>	ENSG00000130723	proline-rich coiled-coil 2B	ENSMUSG00000039262	5.24E-05	NS	0.281292536	4.21E-05	0.000763991
SERBP1<35212>	ENSG00000142864	SERPINE1 mRNA binding protein 1	ENSMUSG00000036371	0.000286998	NS	-0.275797779	6.01E-05	0.001022921
CASC3<41379>	ENSG00000108349	cancer susceptibility candidate 3	ENSMUSG00000078676	0.034179583	NS	-0.275794347	6.01E-05	0.001022921
DAZ4<21851>	ENSG00000205916	deleted in azoospermia 1	ENSMUSG00000010592	0.000307106	NS	0.27504812	6.30E-05	0.001022921
DAZ4<21851>	ENSG00000205916	deleted in azoospermia 4	ENSMUSG00000010592	0.000307106	NS	0.27504812	6.30E-05	0.001022921
DAZ4<21851>	ENSG00000205916	deleted in azoospermia 2	ENSMUSG00000010592	0.000307106	NS	0.27504812	6.30E-05	0.001022921
DAZ2<21851>	ENSG00000205944	deleted in azoospermia 2	ENSMUSG00000010592	0.000307106	NS	0.27504812	6.30E-05	0.001022921
DAZ2<21851>	ENSG00000205944	deleted in azoospermia 1	ENSMUSG00000010592	0.000307106	NS	0.27504812	6.30E-05	0.001022921
DAZ2<21851>	ENSG00000205944	deleted in azoospermia 4	ENSMUSG00000010592	0.000307106	NS	0.27504812	6.30E-05	0.001022921
POLRMT<5271>	ENSG00000099821	polymerase (RNA) mitochondrial (DNA directed)	ENSMUSG00000020329	0.003238017	NS	0.272284266	7.52E-05	0.001189703
UCHL5<29564>	ENSG00000116750	ubiquitin carboxyl-terminal hydrolase L5	ENSMUSG00000018189	8.90E-05	NS	0.268187912	9.72E-05	0.00149931
HADHB<30574>	ENSG00000138029	hydroxyacyl-CoA dehydrogenase/3-ketoacyl-CoA thiolase/enoyl-CoA hydratase (	ENSMUSG00000059447	0.001537647	NS	-0.264622225	0.000121204	0.001823972
UBE2L3<10406>	ENSG00000185651	ubiquitin-conjugating enzyme E2L 3	ENSMUSG00000038965	0.000157863	NS	-0.262531142	0.000137742	0.00198287
SSB<19252>	ENSG00000138385	Sjogren syndrome antigen B (autoantigen La)	ENSMUSG00000068882	0.003866375	NS	0.262205054	0.000140504	0.00198287
SUGP2<41257>	ENSG00000064607	SURP and G patch domain containing 2	ENSMUSG00000036054	0.010966411	NS	-0.262100019	0.000141404	0.00198287
EXOSC1<14547>	ENSG00000171311	exosome component 1	ENSMUSG00000034321	0.012093167	NS	0.261079191	0.000150442	0.002062727
MRPL27<9541>	ENSG00000108826	mitochondrial ribosomal protein L27	ENSMUSG00000024414	0.00012339	0.019573637	0.260442118	0.000156352	0.002097156
BCLAF1<22951>	ENSG00000029363	BCL2-associated transcription factor 1	ENSMUSG00000037608	4.52E-05	NS	-0.255382604	0.000211601	0.002760021
RPL10<34843>	ENSG00000268963	small nucleolar RNA, H/ACA box 70	NA	0.00025738	NS	0.255135463	0.000214718	0.002760021
RPL10<34843>	ENSG00000147403	ribosomal protein L10	ENSMUSG00000058443	0.00025738	NS	0.255135463	0.000214718	0.002760021
RPL10<34843>	ENSG00000147403	small nucleolar RNA, H/ACA box 70	ENSMUSG00000058443	0.00025738	NS	0.255135463	0.000214718	0.002760021
RPL10<34843>	ENSG00000268963	ribosomal protein L10	NA	0.00025738	NS	0.255135463	0.000214718	0.002760021
FKBP3<4491>	ENSG00000100442	FK506 binding protein 3, 25kDa	ENSMUSG00000020949	0.003299934	NS	0.252895409	0.00024499	0.003084874
TRA2B<27060>	ENSG00000136527	transformer 2 beta homolog (Drosophila)	ENSMUSG00000022858	0.000178742	NS	0.247861295	0.000328091	0.004038664

ILF3<16277>	ENSG00000129351	interleukin enhancer binding factor 3, 90kDa	ENSMUSG00000032178	0.00093189	NS		-0.247559235	0.000333828	0.004038664
SYNCRIP<12302>	ENSG00000135316	synaptotagmin binding, cytoplasmic RNA interacting protein	ENSMUSG00000032423	3.33E-05		3.33E-06	0.246212319	0.000360564	0.004243435
YWHAE<22850>	ENSG00000108953	tyrosine 3-monooxygenase/tryptophan 5-monooxygenase activation protein, ep	ENSMUSG00000020849	0.000675704	NS		0.246021472	0.000364509	0.004243435
PPRC1<28108>	ENSG00000148840	peroxisome proliferator-activated receptor gamma, coactivator-related 1	ENSMUSG00000055491	0.000241276	NS		-0.241152118	0.00047978	0.005421105
MRPL42<1683>	ENSG00000198015	mitochondrial ribosomal protein L42	ENSMUSG00000062981	0.002326517	NS		0.240737471	0.000491013	0.005421105
PRPF8<30120>	ENSG00000174231	pre-mRNA processing factor 8	ENSMUSG00000020850	0.003342552	NS		-0.2407004	0.000492029	0.005421105
HNRNPUL1<13569>	ENSG00000105323	heterogeneous nuclear ribonucleoprotein U-like 1	ENSMUSG00000040725	0.000190882		0.032201695	-0.239380945	0.000529488	0.005731475
LUC7L<6801>	ENSG00000007392	LUC7-like (S. cerevisiae)	ENSMUSG00000024188	0.000410288	NS		0.236982996	0.000604394	0.006429502
TUBB<21499>	ENSG00000227739	tubulin, beta class I	NA	0.000241896	NS		0.2365759	0.000618043	0.006463263
TUBB<21499>	ENSG00000232421	tubulin, beta class I	NA	0.000241896	NS		0.2365759	0.000618043	0.006463263
TUBB<21499>	ENSG00000183311	tubulin, beta class I	NA	0.000241896	NS		0.2365759	0.000618043	0.006463263
TUBB<21499>	ENSG00000196230	tubulin, beta class I	ENSMUSG00000001525	0.000241896	NS		0.2365759	0.000618043	0.006463263
TUBB<21499>	ENSG00000232575	tubulin, beta class I	NA	0.000241896	NS		0.2365759	0.000618043	0.006463263
TUBB<21499>	ENSG00000224156	tubulin, beta class I	NA	0.000241896	NS		0.2365759	0.000618043	0.006463263
TUBB<21499>	ENSG00000229684	tubulin, beta class I	NA	0.000241896	NS		0.2365759	0.000618043	0.006463263
TUBB<21499>	ENSG00000235067	tubulin, beta class I	NA	0.000241896	NS		0.2365759	0.000618043	0.006463263
RBM25<14816>	ENSG00000119707	RNA binding motif protein 25	ENSMUSG00000010608	0.001717967	NS		-0.234427838	0.000694896	0.007145847
EIF5B<11352>	ENSG00000158417	eukaryotic translation initiation factor 5B	ENSMUSG00000026083	0.025107979	NS		-0.231557576	0.000811373	0.008206838
EIF3L<42339>	ENSG00000100129	eukaryotic translation initiation factor 3, subunit L	ENSMUSG00000033047	0.000793361	NS		0.229409513	0.000910028	0.009049451
CTNNA1<35899>	ENSG00000044115	catenin (cadherin-associated protein), alpha 1, 102kDa	ENSMUSG00000037815	0.004770825		0.038939273	0.229122556	0.000924012	0.009049451
SNRPD1<37618>	ENSG00000167088	small nuclear ribonucleoprotein D1 polypeptide 16kDa	ENSMUSG00000002477	0.001728181	NS		-0.228401043	0.00096005	0.009255482
GRB2<3367>	ENSG00000177885	growth factor receptor-bound protein 2	ENSMUSG00000059923	0.001230884	NS		0.228016602	0.000979776	0.009300335
MBNL3<14134>	ENSG00000076770	muscleblind-like splicing regulator 3	ENSMUSG00000036109	0.001051119	NS		0.226916827	0.001038281	0.009706354
USP10<38433>	ENSG00000103194	ubiquitin specific peptidase 10	ENSMUSG00000031826	0.026888273	NS		-0.225280895	0.001131262	0.01017919
SNRPD3<41742>	ENSG00000100028	small nuclear ribonucleoprotein D3 polypeptide 18kDa	ENSMUSG00000020180	0.002434029	NS		-0.224474942	0.001179826	0.01017919
HMGN2<17184>	ENSG00000198830	high mobility group nucleosomal binding domain 2	ENSMUSG00000091775	0.048192799	NS		-0.224436498	0.00118219	0.01017919
EIF5B<14216>	ENSG00000158417	eukaryotic translation initiation factor 5B	ENSMUSG00000026083	0.045468243		0.027253817	-0.224356177	0.001187142	0.01017919
RPS13<27535>	ENSG00000110700	small nucleolar RNA, C/D box 14B	ENSMUSG00000090862	0.004054462	NS		0.224239472	0.001194371	0.01017919
RPS13<27535>	ENSG00000110700	uncharacterized LOC100508408	ENSMUSG00000090862	0.004054462	NS		0.224239472	0.001194371	0.01017919
RPS13<27535>	ENSG00000110700	ribosomal protein S13	ENSMUSG00000090862	0.004054462	NS		0.224239472	0.001194371	0.01017919
XRN2<28616>	ENSG00000088930	5'-3' exoribonuclease 2	ENSMUSG00000027433	0.001885931		0.034267154	0.224106977	0.001202628	0.01017919
TNRC6B<7602>	ENSG00000100354	trinucleotide repeat containing 6B	ENSMUSG00000047888	0.001439	NS		0.224079517	0.001204345	0.01017919
TBRG4<40946>	ENSG00000136270	transforming growth factor beta regulator 4	ENSMUSG00000000384	0.000750031		0.038790857	0.223590042	0.001235344	0.010300098
TBRG4<40946>	ENSG00000136270	small nucleolar RNA, H/ACA box 5B	ENSMUSG00000000384	0.000750031		0.038790857	0.223590042	0.001235344	0.010300098
CCT3<1953>	ENSG00000163468	uncharacterized LOC101927137	ENSMUSG00000001416	0.001362393	NS		-0.222906973	0.001279825	0.010401232
CCT3<1953>	ENSG00000163468	chaperonin containing TCP1, subunit 3 (gamma)	ENSMUSG00000001416	0.001362393	NS		-0.222906973	0.001279825	0.010401232
ZC3H10<15786>	ENSG00000135482	zinc finger CCCH-type containing 10	ENSMUSG00000039810	0.004242583	NS		0.222886378	0.001281189	0.010401232
RC3H2<665>	ENSG00000056586	ring finger and CCCH-type domains 2	ENSMUSG00000075376	0.002090694	NS		0.221855253	0.001351189	0.01082706
CPEB3<33837>	ENSG00000107864	cytoplasmic polyadenylation element binding protein 3	ENSMUSG00000039652	0.004671093	NS		-0.220564631	0.001443737	0.01142033
FARSA<26519>	ENSG00000179115	phenylalanyl-tRNA synthetase, alpha subunit	ENSMUSG00000003808	0.001584671	NS		0.220239229	0.001467969	0.011465024
PTPN1<5915>	ENSG00000196396	protein tyrosine phosphatase, non-receptor type 1	ENSMUSG00000027540	0.000524732	NS		-0.219635794	0.001513892	0.011675892
ANKHD1<4250>	ENSG00000131503	ankyrin repeat and KH domain containing 1	ENSMUSG00000024483	0.004341299	NS		-0.218163936	0.001631466	0.01242734
ANKHD1<4250>	ENSG00000131503	ANKHD1-EIF4EBP3 readthrough	ENSMUSG00000024483	0.004341299	NS		-0.218163936	0.001631466	0.01242734
TRMT6<23961>	ENSG00000089195	tRNA methyltransferase 6 homolog (S. cerevisiae)	ENSMUSG00000037376	0.003270482		0.010713003	0.217360729	0.00169909	0.012602283
TRA2A<27938>	ENSG00000164548	transformer 2 alpha homolog (Drosophila)	ENSMUSG00000029817	0.000593928	NS		0.217211072	0.001711969	0.012602283
EIF3H<26684>	ENSG00000147677	eukaryotic translation initiation factor 3, subunit H	ENSMUSG00000022312	0.002410961	NS		-0.217115648	0.001720228	0.012602283
ZC3H12D<18687>	ENSG00000178199	zinc finger CCCH-type containing 12D	ENSMUSG00000039981	0.00213573	NS		-0.216933039	0.001736133	0.012602283
U2SURP<35540>	ENSG00000163714	U2 snRNP-associated SURP domain containing	ENSMUSG00000032407	0.001363491	NS		0.216479261	0.001776241	0.012743496
RPL13<37792>	ENSG00000167526	ribosomal protein L13	ENSMUSG00000059835	0.00366267	NS		0.215888184	0.001829751	0.012841866
RPL13<37792>	ENSG00000167526	small nucleolar RNA, C/D box 68	ENSMUSG00000059835	0.00366267	NS		0.215888184	0.001829751	0.012841866
ILF3<29229>	ENSG00000129351	interleukin enhancer binding factor 3, 90kDa	ENSMUSG00000032178	0.0006366	NS		-0.215868275	0.001831579	0.012841866
DHX9<41515>	ENSG00000135829	DEAH (Asp-Glu-Ala-His) box helicase 9	ENSMUSG00000042699	0.002946585	NS		0.214134173	0.001997314	0.013846548
FSCN1<40466>	ENSG00000075618	fascin homolog 1, actin-bundling protein (Strongylocentrotus purpuratus)	ENSMUSG00000029581	0.003798358	NS		-0.213788863	0.002031904	0.013929831
MECP2<19399>	ENSG00000169057	methyl CpG binding protein 2 (Rett syndrome)	ENSMUSG00000031393	0.000294421	NS		-0.211991602	0.00222091	0.014351346
MECP2<19399>	ENSG00000268563	methyl CpG binding protein 2 (Rett syndrome)	NA	0.000294421	NS		-0.211991602	0.00222091	0.014351346
RBM5<32995>	ENSG00000003756	RNA binding motif protein 5	ENSMUSG00000032580	0.029834302	NS		-0.211924325	0.002228286	0.014351346
MAZ<33996>	ENSG00000103495	MYC-associated zinc finger protein (purine-binding transcription factor)	ENSMUSG00000030678	0.012219743	NS		0.211902357	0.0022307	0.014351346
EIF2S2<33717>	ENSG00000125977	eukaryotic translation initiation factor 2, subunit 2 beta, 38kDa	ENSMUSG00000074656	0.000446262	NS		-0.211661395	0.002257327	0.014351346
UBC<24310>	ENSG00000150991	ubiquitin C	ENSMUSG00000008348	0.003271088	NS		0.211589999	0.002265272	0.014351346
TROVE2<31115>	ENSG00000116747	TROVE domain family, member 2	ENSMUSG00000018199	2.11E-05	NS		-0.2112543	0.002302971	0.014351346
POU5F1<32319>	ENSG00000237582	POU class 5 homeobox 1	NA	0.016684607	NS		0.211108762	0.002319491	0.014351346
POU5F1<32319>	ENSG00000230336	POU class 5 homeobox 1	NA	0.016684607	NS		0.211108762	0.002319491	0.014351346
POU5F1<32319>	ENSG00000233911	POU class 5 homeobox 1	NA	0.016684607	NS		0.211108762	0.002319491	0.014351346
POU5F1<32319>	ENSG00000204531	POU class 5 homeobox 1	ENSMUSG00000024406	0.016684607	NS		0.211108762	0.002319491	0.014351346
POU5F1<32319>	ENSG00000206454	POU class 5 homeobox 1	NA	0.016684607	NS		0.211108762	0.002319491	0.014351346
POU5F1<32319>	ENSG00000235068	POU class 5 homeobox 1	NA	0.016684607	NS		0.211108762	0.002319491	0.014351346
POU5F1<32319>	ENSG00000229094	POU class 5 homeobox 1	NA	0.016684607	NS		0.211108762	0.002319491	0.014351346
CCT4<10483>	ENSG00000115484	chaperonin containing TCP1, subunit 4 (delta)	ENSMUSG00000007739	0.004913205	NS		0.211097091	0.00232082	0.014351346
NUPL2<4389>	ENSG00000136243	nucleoporin like 2	ENSMUSG00000048439	0.021662522		0.017255139	0.211079929	0.002322777	0.014351346
SRPR<754>	ENSG00000182934	signal recognition particle receptor (docking protein)	ENSMUSG00000032042	0.009332714		0.023383257	0.211051782	0.002325988	0.014351346

HIST1H1C<10725>	ENSG00000187837	histone cluster 1, H1c	ENSMUSG00000036181	0.006289837	NS	-0.210685877	0.002368107	0.014466555
RPN1<33630>	ENSG00000163902	ribophorin I	ENSMUSG00000030062	0.001640976	0.024185751	0.209756354	0.002478236	0.014990898
MTHFSD<9250>	ENSG00000103248	methenyltetrahydrofolate synthetase domain containing	ENSMUSG00000031816	0.00289025	NS	0.208294793	0.002660835	0.015939177
EZR<31292>	ENSG00000092820	ezrin	ENSMUSG00000052397	0.003670041	0.020786435	0.207567788	0.002756134	0.016351295
HDLBP<14078>	ENSG00000115677	high density lipoprotein binding protein	ENSMUSG00000034088	0.004706013	NS	0.206736435	0.002868898	0.016858191
HIST1H1D<13208>	ENSG00000124575	histone cluster 1, H1d	ENSMUSG00000052565	0.006942946	NS	-0.206174877	0.002947415	0.01715618
SPEN<23872>	ENSG00000065526	spen homolog, transcriptional regulator (Drosophila)	ENSMUSG00000040761	0.015866241	NS	0.205668925	0.003019819	0.01718062
ZGPAT<40429>	ENSG00000197114	zinc finger, CCCH-type with G patch domain	ENSMUSG00000027582	0.003035213	NS	0.205592724	0.003030862	0.01718062
MRPL9<37880>	ENSG00000143436	mitochondrial ribosomal protein L9	ENSMUSG00000028140	0.004378119	NS	-0.205563204	0.00303515	0.01718062
YWHAZ<43361>	ENSG00000164924	tyrosine 3-monooxygenase/tryptophan 5-monooxygenase activation protein, ze	ENSMUSG00000022285	0.002767809	NS	0.20509707	0.00310359	0.017286606
SLC25A5<9937>	ENSG00000005022	solute carrier family 25 (mitochondrial carrier; adenine nucleotide translocator),	ENSMUSG00000016319	0.004942933	NS	-0.205054507	0.003109908	0.017286606
TRMT44<6757>	ENSG00000155275	tRNA methyltransferase 44 homolog (S. cerevisiae)	ENSMUSG00000029097	0.003440553	NS	0.204214229	0.003237052	0.017832688
SMG1<38649>	ENSG00000157106	SMG1 phosphatidylinositol 3-kinase-related kinase	ENSMUSG00000030655	0.011336989	NS	-0.203378757	0.003368112	0.01821884
SMG1<38649>	ENSG00000157106	bolA family member 2	ENSMUSG00000030655	0.011336989	NS	-0.203378757	0.003368112	0.01821884
SMG1<38649>	ENSG00000157106	serine/threonine-protein kinase SMG1-like	ENSMUSG00000030655	0.011336989	NS	-0.203378757	0.003368112	0.01821884
HSPB1<40040>	ENSG00000106211	heat shock 27kDa protein 1	ENSMUSG00000004951	0.00101423	NS	0.203257933	0.003387457	0.01821884
HSPB1<40040>	LRG_248	heat shock 27kDa protein 1	NA	0.00101423	NS	0.203257933	0.003387457	0.01821884
RBMY1B<26021>	ENSG00000242875	RNA binding motif protein, Y-linked, family 1, member D	ENSMUSG00000096520	0.006599216	0.020886776	0.203206445	0.003395732	0.01821884
RBMY1B<26021>	ENSG00000242875	RNA binding motif protein, Y-linked, family 1, member B	ENSMUSG00000096520	0.006599216	0.020886776	0.203206445	0.003395732	0.01821884
ALKBH5<1822>	ENSG00000091542	alkB, alkylation repair homolog 5 (E. coli)	ENSMUSG00000042650	0.005242551	NS	0.202931845	0.00344017	0.018298146
YWHAE<38625>	ENSG00000108953	tyrosine 3-monooxygenase/tryptophan 5-monooxygenase activation protein, ep	ENSMUSG00000020849	0.014113434	NS	0.202677153	0.003481856	0.018361582
RBM14<36437>	ENSG00000239306	RNA binding motif protein 14	ENSMUSG00000006456	0.006275262	NS	0.20183001	0.003623812	0.018813893
RPLP0<9694>	ENSG00000089157	ribosomal protein, large, P0	ENSMUSG00000067274	0.002208648	0.03257704	-0.201694769	0.003646952	0.018813893
HMGN5<43119>	ENSG00000198157	high mobility group nucleosome binding domain 5	ENSMUSG00000031245 NS		0.034138461	0.20162406	0.003659104	0.018813893
ZCCHC6<34717>	ENSG00000083223	zinc finger, CCHC domain containing 6	ENSMUSG00000035248	0.004105393	NS	0.200838702	0.003796547	0.019359252
HLA-A<31692>	ENSG00000223980	major histocompatibility complex, class I, A	NA	0.02334258	NS	0.199706662	0.00400286	0.020243972
HLA-A<31692>	ENSG00000235657	major histocompatibility complex, class I, A	NA	0.02334258	NS	0.199706662	0.00400286	0.020243972
HLA-A<31692>	ENSG00000229215	major histocompatibility complex, class I, A	NA	0.02334258	NS	0.199706662	0.00400286	0.020243972
HLA-A<31692>	ENSG00000227715	major histocompatibility complex, class I, A	NA	0.02334258	NS	0.199706662	0.00400286	0.020243972
HLA-A<31692>	ENSG00000231834	major histocompatibility complex, class I, A	NA	0.02334258	NS	0.199706662	0.00400286	0.020243972
HLA-A<31692>	ENSG00000224320	major histocompatibility complex, class I, A	NA	0.02334258	NS	0.199706662	0.00400286	0.020243972
HLA-A<31692>	ENSG00000206505	major histocompatibility complex, class I, A	NA	0.02334258	NS	0.199706662	0.00400286	0.020243972
HLA-A<31692>	ENSG00000206503	major histocompatibility complex, class I, A	NA	0.02334258	NS	0.199706662	0.00400286	0.020243972
GAPDH<38660>	ENSG00000111640	glyceraldehyde-3-phosphate dehydrogenase	ENSMUSG00000081607	0.014967751	NS	-0.199503457	0.004040946	0.020270436
NSUN2<35672>	ENSG00000037474	NOP2/Sun RNA methyltransferase family, member 2	ENSMUSG00000021595	0.013544691	NS	0.199050366	0.004127045	0.020397112
C1orf131<6226>	ENSG00000143633	chromosome 1 open reading frame 131	ENSMUSG00000031984	0.020687809	NS	-0.199022906	0.004132316	0.020397112
HMGB1<38758>	ENSG00000189403	high mobility group box 1	ENSMUSG00000066551	0.038166724	NS	-0.198450364	0.004243603	0.020780183
LRP1<10195>	ENSG00000123384	low density lipoprotein receptor-related protein 1	ENSMUSG00000040249	0.002661483	NS	-0.197711002	0.004391305	0.021334135
YBX1<42630>	ENSG00000065978	Y box binding protein 1	ENSMUSG00000028639	0.004760296	0.034479557	-0.197216721	0.00449261	0.021655784
ALDOA<37981>	ENSG00000149925	aldolase A, fructose-bisphosphate	ENSMUSG00000059343	0.00189745	0.022800018	0.196979878	0.004541893	0.021723628
SYNCRIP<36496>	ENSG00000135316	synaptotagmin binding, cytoplasmic RNA interacting protein	ENSMUSG00000032423	0.023346388	NS	0.19615745	0.004716823	0.022336479
S100A16<28521>	ENSG00000188643	S100 calcium binding protein A16	ENSMUSG00000074457	0.006801871	NS	0.196039372	0.004742429	0.022336479
DDX18<15183>	ENSG00000088205	DEAD (Asp-Glu-Ala-Asp) box polypeptide 18	ENSMUSG00000001674	0.000771937	NS	-0.195501841	0.004860582	0.022719539
LRRC47<1792>	ENSG00000130764	leucine rich repeat containing 47	ENSMUSG00000029028	0.011742161	NS	0.19476042	0.005027888	0.023324864
GTPBP1<3658>	ENSG00000100226	GTP binding protein 1	ENSMUSG00000042535	0.009503952	NS	-0.1932357	0.005388287	0.024810247
YTHDF2<39289>	ENSG00000198492	YTH domain family, member 2	ENSMUSG00000040025	0.001601808	NS	-0.19304142	0.005435834	0.024843775
CELF5<32087>	ENSG00000161082	CUGBP, Elav-like family member 5	ENSMUSG00000034818	0.001651337	NS	-0.191730203	0.005766688	0.026162107
URB1<12391>	ENSG00000142207	URB1 ribosome biogenesis 1 homolog (S. cerevisiae)	ENSMUSG00000039929	0.019594019	NS	0.191128828	0.005924372	0.026681296
EIF5<13444>	ENSG00000100664	eukaryotic translation initiation factor 5	ENSMUSG00000021282	0.003282848	NS	-0.190919445	0.005980172	0.026737436
EEF1G<32502>	ENSG00000254772	eukaryotic translation elongation factor 1 gamma	ENSMUSG00000071644	0.001974881	NS	-0.190653769	0.00605165	0.02686236
EEF1G<32502>	ENSG00000254772	microRNA 3654	ENSMUSG00000071644	0.001974881	NS	-0.190653769	0.00605165	0.02686236
HNRNPR<44782>	ENSG00000125944	heterogeneous nuclear ribonucleoprotein R	ENSMUSG00000066037	0.006755414	NS	-0.190016009	0.006226363	0.027440471
MATR3<34064>	ENSG00000015479	small nucleolar RNA host gene 4 (non-protein coding)	ENSMUSG00000037236	0.006224132	NS	0.189737976	0.00630393	0.027585282
MATR3<34064>	ENSG00000015479	matrin 3	ENSMUSG00000037236	0.006224132	NS	0.189737976	0.00630393	0.027585282
CNBP<42912>	ENSG00000169714	CCHC-type zinc finger, nucleic acid binding protein	ENSMUSG00000030057	0.027384795	NS	-0.189229965	0.00644789	0.027998325
RSL1D1<4589>	ENSG00000171490	ribosomal L1 domain containing 1	ENSMUSG00000005846	0.005603209	NS	0.189086486	0.006489077	0.027998325
EIF1B<36924>	ENSG00000114784	eukaryotic translation initiation factor 1B	ENSMUSG00000006941	0.003744713	NS	0.18877962	0.006577956	0.028184714
LARP6<13088>	ENSG00000166173	La ribonucleoprotein domain family, member 6	ENSMUSG00000034839	0.002482603	NS	-0.188551015	0.006644873	0.02827508
R3HDM2<22847>	ENSG00000179912	R3H domain containing 2	ENSMUSG00000025404	0.000689967	NS	-0.188279847	0.006725035	0.028420182
IGF2BP1<7133>	ENSG00000159217	insulin-like growth factor 2 mRNA binding protein 1	ENSMUSG00000013415	0.016591742	NS	-0.188010052	0.006805647	0.028565199
ZNF579<26226>	ENSG00000218891	zinc finger protein 579	ENSMUSG00000051550	0.004087478	0.04755316	-0.187736138	0.00688837	0.028621143
GOLGB1<8237>	ENSG00000173230	golgin B1	ENSMUSG00000034243	0.004387038	NS	-0.18765925	0.006911751	0.028621143
MRPL54<26507>	ENSG00000183617	mitochondrial ribosomal protein L54	ENSMUSG00000034932	0.003913966	NS	0.185367709	0.007641882	0.031433608
C1orf52<40562>	ENSG00000162642	chromosome 1 open reading frame 52	ENSMUSG00000036873	0.005850244	0.008072827	0.184723771	0.007859042	0.032072232
SRSF1<29065>	ENSG00000136450	serine/arginine-rich splicing factor 1	ENSMUSG00000018379	0.005858651	NS	-0.184600887	0.007901101	0.032072232
RPL36A<33436>	ENSG00000269315	ribosomal protein L36a	NA	0.003309457	0.027743943	0.183674797	0.008224561	0.032478548
RPL36A<33436>	ENSG00000241343	ribosomal protein L36a	NA	0.003309457	0.027743943	0.183674797	0.008224561	0.032478548
RPL36A<33436>	ENSG00000241343	RPL36A-HNRNPH2 readthrough	NA	0.003309457	0.027743943	0.183674797	0.008224561	0.032478548
RPL36A<33436>	ENSG00000269315	RPL36A-HNRNPH2 readthrough	NA	0.003309457	0.027743943	0.183674797	0.008224561	0.032478548
HNRNPH2<33436>	ENSG00000268642	RPL36A-HNRNPH2 readthrough	NA	0.003309457	0.027743943	0.183674797	0.008224561	0.032478548

HNRNPH2<33436>	ENSG00000126945	RPL36A-HNRNPH2 readthrough	ENSMUSG00000045427	0.003309457	0.027743943	0.183674797	0.008224561	0.032478548
HNRNPH2<33436>	ENSG00000126945	heterogeneous nuclear ribonucleoprotein H2 (H')	ENSMUSG00000045427	0.003309457	0.027743943	0.183674797	0.008224561	0.032478548
HNRNPH2<33436>	ENSG00000268642	heterogeneous nuclear ribonucleoprotein H2 (H')	NA	0.003309457	0.027743943	0.183674797	0.008224561	0.032478548
CIRH1A<5752>	ENSG00000141076	cirrhosis, autosomal recessive 1A (cirhin)	ENSMUSG00000041438	0.000924172	NS	-0.183620563	0.008243863	0.032478548
CIRH1A<5752>	ENSG00000262788	cirrhosis, autosomal recessive 1A (cirhin)	NA	0.000924172	NS	-0.183620563	0.008243863	0.032478548
LARP4<36618>	ENSG00000161813	La ribonucleoprotein domain family, member 4	ENSMUSG00000023025	0.02114917	0.009548354	-0.183446878	0.008305949	0.032478548
PCBP2<7339>	ENSG00000197111	poly(rC) binding protein 2	ENSMUSG00000056851	0.006138672	0.013085902	0.183441386	0.008307919	0.032478548
GANAB<29228>	ENSG00000089597	glucosidase, alpha; neutral AB	ENSMUSG00000071650	0.005563424	NS	0.183415986	0.008317035	0.032478548
RNMTL1<10811>	ENSG00000171861	RNA methyltransferase like 1	ENSMUSG00000038046	0.003075634	NS	0.182520788	0.008644053	0.033543275
ZCCHC9<38143>	ENSG00000131732	zinc finger, CCHC domain containing 9	ENSMUSG00000021621	0.011787794	0.008192344	0.182036804	0.008825563	0.033834138
PARP14<38933>	ENSG00000173193	poly (ADP-ribose) polymerase family, member 14	ENSMUSG00000034422	0.005225758	NS	0.182028566	0.008828681	0.033834138
SRSF7<18707>	ENSG00000115875	serine/arginine-rich splicing factor 7	ENSMUSG00000024097	0.027728383	NS	-0.181824676	0.008906178	0.033842454
C2orf15<26989>	ENSG00000241962	mitochondrial ribosomal protein L30	ENSMUSG00000026087	0.004151081	NS	-0.181734744	0.008940551	0.033842454
C2orf15<26989>	ENSG00000241962	uncharacterized LOC101927110	ENSMUSG00000026087	0.004151081	NS	-0.181734744	0.008940551	0.033842454
HNRNPK<43327>	ENSG00000165119	heterogeneous nuclear ribonucleoprotein K	ENSMUSG00000021546	0.011928987	0.024470749	0.181139547	0.009171024	0.034503182
NOMO1<32633>	ENSG00000103512	NODAL modulator 1	ENSMUSG00000030835	0.008754379	NS	0.179872952	0.00967907	0.036030354
NOMO1<32633>	ENSG00000103512	nodal modulator 1-like	ENSMUSG00000030835	0.008754379	NS	0.179872952	0.00967907	0.036030354
HMGN5<26669>	ENSG00000198157	high mobility group nucleosome binding domain 5	ENSMUSG00000031245	0.003225765	NS	-0.179837254	0.009693742	0.036030354
EIF3E<2014>	ENSG00000104408	eukaryotic translation initiation factor 3, subunit E	ENSMUSG00000022336	0.00220526	NS	0.179650526	0.009770814	0.036099355
SMG1<33714>	ENSG00000157106	SMG1 phosphatidylinositol 3-kinase-related kinase	ENSMUSG00000030655	0.003602438	NS	0.179471349	0.009845277	0.036157952
SMG1<33714>	ENSG00000157106	bolA family member 2	ENSMUSG00000030655	0.003602438	NS	0.179471349	0.009845277	0.036157952
SMG1<33714>	ENSG00000157106	serine/threonine-protein kinase SMG1-like	ENSMUSG00000030655	0.003602438	NS	0.179471349	0.009845277	0.036157952
RBMX2<43192>	ENSG00000134597	RNA binding motif protein, X-linked 2	ENSMUSG00000031107	0.015989403	NS	0.179102698	0.010000064	0.036509095
YBX3<34396>	ENSG00000060138	Y box binding protein 3	ENSMUSG00000030189	0.012794975	NS	0.178741598	0.010153762	0.036852176
SUB1<34960>	ENSG00000113387	SUB1 homolog (S. cerevisiae)	ENSMUSG00000022205	0.007223203	NS	0.17856654	0.010229022	0.036908218
DDX56<26784>	ENSG00000136271	DEAD (Asp-Glu-Ala-Asp) box helicase 56	ENSMUSG00000004393	0.000910507	NS	-0.178421689	0.010291668	0.036918374
U2AF2<22618>	ENSG00000063244	U2 small nuclear RNA auxiliary factor 2	ENSMUSG00000030435	0.016253228	NS	0.178269972	0.010357647	0.036940289
POLR2G<20769>	ENSG00000168002	polymerase (RNA) II (DNA directed) polypeptide G	ENSMUSG00000071662	0.04892077	NS	-0.177822373	0.010554481	0.037425943
ACO1<24917>	ENSG00000122729	aconitase 1, soluble	ENSMUSG00000028405	0.00054499	NS	-0.177531983	0.01068394	0.03766852
CLASRP<34743>	ENSG00000104859	CLK4-associating serine/arginine rich protein	ENSMUSG00000061028	0.011657956	NS	-0.177323287	0.010777844	0.037783678
CCT6A<40582>	ENSG00000146731	chaperonin containing TCP1, subunit 6A (zeta 1)	ENSMUSG00000029447	0.006904525	NS	-0.177126947	0.010866851	0.037804318
FKBP4<32547>	ENSG00000004478	FK506 binding protein 4, 59kDa	ENSMUSG00000030357	0.002850416	NS	0.177040448	0.01090627	0.037804318
ZCCHC8<37704>	ENSG00000033030	zinc finger, CCHC domain containing 8	ENSMUSG00000029427	0.007349591	NS	0.176402002	0.011201137	0.038531581
SCAF8<6448>	ENSG00000213079	SR-related CTD-associated factor 8	ENSMUSG00000046201	0.011877149	NS	0.176316876	0.011240979	0.038531581
SERBP1<16360>	ENSG00000142864	SERPINE1 mRNA binding protein 1	ENSMUSG00000036371	0.006803808	NS	0.175998339	0.011391175	0.038707936
QKI<31460>	ENSG00000112531	QKI, KH domain containing, RNA binding	ENSMUSG00000062078	0.027672956	NS	-0.175942046	0.011417902	0.038707936
HLA-A<20186>	ENSG00000223980	major histocompatibility complex, class I, A	NA	0.007077285	NS	-0.175808865	0.011481353	0.038710344
HLA-A<20186>	ENSG00000235657	major histocompatibility complex, class I, A	NA	0.007077285	NS	-0.175808865	0.011481353	0.038710344
HLA-A<20186>	ENSG00000229215	major histocompatibility complex, class I, A	NA	0.007077285	NS	-0.175808865	0.011481353	0.038710344
HLA-A<20186>	ENSG00000227715	major histocompatibility complex, class I, A	NA	0.007077285	NS	-0.175808865	0.011481353	0.038710344
HLA-A<20186>	ENSG00000231834	major histocompatibility complex, class I, A	NA	0.007077285	NS	-0.175808865	0.011481353	0.038710344
HLA-A<20186>	ENSG00000224320	major histocompatibility complex, class I, A	NA	0.007077285	NS	-0.175808865	0.011481353	0.038710344
HLA-A<20186>	ENSG00000206505	major histocompatibility complex, class I, A	NA	0.007077285	NS	-0.175808865	0.011481353	0.038710344
HLA-A<20186>	ENSG00000206503	major histocompatibility complex, class I, A	NA	0.007077285	NS	-0.175808865	0.011481353	0.038710344
PPIA<12548>	ENSG00000196262	peptidylprolyl isomerase A (cyclophilin A)	ENSMUSG00000055795	NS	0.020780062	-0.175311838	0.0117209	0.039303235
PPIA<12548>	ENSG00000196262	peptidyl-prolyl cis-trans isomerase A-like	ENSMUSG00000055795	NS	0.020780062	-0.175311838	0.0117209	0.039303235
UTP11L<6618>	ENSG00000183520	UTP11-like, U3 small nucleolar ribonucleoprotein, (yeast)	ENSMUSG00000028907	0.011173278	NS	-0.174733117	0.012005357	0.040039498
CDKN2A<23493>	LRG_11	cyclin-dependent kinase inhibitor 2A	NA	0.004790298	NS	0.174121444	0.012312596	0.04084341
CDKN2A<23493>	ENSG00000147889	cyclin-dependent kinase inhibitor 2A	NA	0.004790298	NS	0.174121444	0.012312596	0.04084341
KRT18<6876>	ENSG00000111057	keratin 18	ENSMUSG00000023043	NS	0.033697937	0.173538605	0.012611765	0.041535882
RPGR<16498>	ENSG00000156313	retinitis pigmentosa GTPase regulator	ENSMUSG00000031174	0.045691825	0.038586838	0.173453479	0.01265599	0.041535882
HNRNPUL2<39978>	ENSG00000214753	heterogeneous nuclear ribonucleoprotein U-like 2	ENSMUSG00000071659	0.015083064	NS	0.171949354	0.013460207	0.043941532
FAM103A1<18722>	ENSG00000169612	family with sequence similarity 103, member A1	ENSMUSG00000074826	0.010863455	NS	0.171624639	0.013639594	0.044292774
CDKN2A<27364>	LRG_11	cyclin-dependent kinase inhibitor 2A	NA	0.001316523	NS	-0.171410451	0.013759065	0.044446806
CDKN2A<27364>	ENSG00000147889	cyclin-dependent kinase inhibitor 2A	NA	0.001316523	NS	-0.171410451	0.013759065	0.044446806
HNRNPU<40805>	ENSG00000153187	heterogeneous nuclear ribonucleoprotein U (scaffold attachment factor A)	ENSMUSG00000039630	NS	0.004221853	0.171133104	0.013915127	0.04471685
ZFR2<13700>	ENSG00000105278	zinc finger RNA binding protein 2	ENSMUSG00000034949	0.009145861	NS	-0.170728069	0.014145826	0.045222679
IGF2BP3<39392>	ENSG00000136231	insulin-like growth factor 2 mRNA binding protein 3	ENSMUSG00000029814	0.00605693	NS	-0.170126693	0.014494534	0.046098583
RPS12<28046>	ENSG00000112306	ribosomal protein S12	ENSMUSG00000061983	0.028958388	NS	0.169838363	0.014664375	0.046399602
TRMT10C<31569>	ENSG00000174173	tRNA methyltransferase 10 homolog C (S. cerevisiae)	ENSMUSG00000044763	0.010864711	NS	-0.169405181	0.014922815	0.046976428
DUSP14<4279>	ENSG00000161326	dual specificity phosphatase 14	ENSMUSG00000018648	0.008911398	NS	0.169201976	0.015045414	0.047121919
MDH2<20291>	ENSG00000262847	malate dehydrogenase 2, NAD (mitochondrial)	NA	0.044718915	NS	-0.168538129	0.015452092	0.04815121
MDH2<20291>	ENSG00000146701	malate dehydrogenase 2, NAD (mitochondrial)	ENSMUSG00000019179	0.044718915	NS	-0.168538129	0.015452092	0.04815121
RBFOX2<25570>	ENSG00000100320	RNA binding protein, fox-1 homolog (C. elegans) 2	ENSMUSG00000033565	0.002982363	NS	-0.168311584	0.015593056	0.048244366
POLR1E<15442>	ENSG00000137054	polymerase (RNA) I polypeptide E, 53kDa	ENSMUSG00000028318	0.009364599	NS	0.16817497	0.015678604	0.048244366
SRRM2<1087>	ENSG00000167978	serine/arginine repetitive matrix 2	ENSMUSG00000039218	0.010821826	NS	0.168114558	0.015716565	0.048244366
SUPV3L1<17253>	ENSG00000156502	suppressor of var1, 3-like 1 (S. cerevisiae)	ENSMUSG00000020079	0.02449602	NS	-0.167711582	0.015971839	0.04867823
DNTTIP2<8100>	ENSG00000067334	deoxynucleotidyltransferase, terminal, interacting protein 2	ENSMUSG00000039756	0.021547235	NS	-0.167642932	0.016015685	0.04867823
SPTBN1<34477>	ENSG00000115306	spectrin, beta, non-erythrocytic 1	ENSMUSG00000020315	0.013585703	NS	0.167504945	0.016104135	0.048707099
SEC61A1<39400>	ENSG00000058262	Sec61 alpha 1 subunit (S. cerevisiae)	ENSMUSG00000030082	0.024389414	NS	0.167146591	0.016335834	0.048831278

ZFC3H1<10529>	ENSG00000133858	zinc finger, C3H1-type containing	ENSMUSG00000034163	0.001794297	NS		-0.167145218	0.016336727	0.048831278
IFIT2<28786>	ENSG00000119922	interferon-induced protein with tetratricopeptide repeats 2	ENSMUSG00000045932	0.027805843	NS		-0.167044303	0.016402501	0.048831278
CDK13<27659>	ENSG00000065883	cyclin-dependent kinase 13	ENSMUSG00000041297	0.048433388	NS		-0.166953684	0.01646176	0.048831278
NAP1L1<19779>	ENSG00000187109	nucleosome assembly protein 1-like 1	ENSMUSG00000079139	0.01182282	NS		0.166672905	0.016646561	0.049143194
SUMO2<18025>	ENSG00000188612	small ubiquitin-like modifier 2	ENSMUSG00000020738	0.006972634	NS		0.165813406	0.017223543	0.050604401
SUMO2<18025>	ENSG00000188612	small ubiquitin-like modifier 3	ENSMUSG00000020738	0.006972634	NS		0.165813406	0.017223543	0.050604401
SUMO2<18025>	ENSG00000188612	small ubiquitin-related modifier 2-like	ENSMUSG00000020738	0.006972634	NS		0.165813406	0.017223543	0.050604401
RPL22<14082>	ENSG00000116251	ribosomal protein L22	ENSMUSG00000028936	0.031111293	NS		-0.165689835	0.017307911	0.050611282
EIF4E<28999>	ENSG00000151247	eukaryotic translation initiation factor 4E	ENSMUSG00000028156	0.004009908	NS		-0.165459858	0.017465887	0.050832331
HSP90AA1<20728>	ENSG00000080824	heat shock protein 90kDa alpha (cytosolic), class A member 1	ENSMUSG00000021270	0.026688378	NS		0.165175646	0.017662851	0.051164218
SNRPA<26681>	ENSG00000077312	small nuclear ribonucleoprotein polypeptide A	ENSMUSG00000071273	0.012196234	NS		-0.164311341	0.018273749	0.052686466
NBPF10<24796>	ENSG00000163386	neuroblastoma breakpoint family, member 14	NA	0.035382773		0.010344076	0.16370516	0.018713083	0.053702188
NBPF10<24796>	ENSG00000163386	neuroblastoma breakpoint family, member 10	NA	0.035382773		0.010344076	0.16370516	0.018713083	0.053702188
NBPF10<24796>	ENSG00000163386	neuroblastoma breakpoint family member 21-like	NA	0.035382773		0.010344076	0.16370516	0.018713083	0.053702188
GRWD1<9470>	ENSG00000105447	glutamate-rich WD repeat containing 1	ENSMUSG00000053801	0.028002468	NS		-0.163409278	0.018930836	0.054075594
RPL14<5712>	ENSG00000188846	ribosomal protein L14	ENSMUSG00000025794	0.025544761	NS		0.16322255	0.019069388	0.054220339
RBMS3<31942>	ENSG00000144642	RNA binding motif, single stranded interacting protein 3	ENSMUSG00000039607	0.048642783	NS		-0.163021405	0.01921962	0.054396814
NAP1L1<16615>	ENSG00000187109	nucleosome assembly protein 1-like 1	ENSMUSG00000079139	0.015984474		0.025122805	0.162886164	0.019321205	0.054434642
CELF2<10176>	ENSG00000048740	CUGBP, Elav-like family member 2	ENSMUSG00000002107	0.005627019	NS		0.162393256	0.019695404	0.055236645
JUN<41956>	ENSG00000177606	jun proto-oncogene	ENSMUSG00000052684	0.001004755	NS		-0.161680668	0.020247475	0.056507167
GTPBP1<23842>	ENSG00000100226	GTP binding protein 1	ENSMUSG00000042535	0.018137671	NS		0.161573574	0.020331593	0.056507167
RPL35<28394>	ENSG00000136942	ribosomal protein L35	ENSMUSG00000078193	0.032039991	NS		-0.161385472	0.020480072	0.056664588
HDGF<19445>	ENSG00000143321	hepatoma-derived growth factor	ENSMUSG00000004897	0.039103962		0.033708738	-0.161266708	0.020574302	0.056671175
DDX49<12828>	ENSG00000105671	DEAD (Asp-Glu-Ala-Asp) box polypeptide 49	ENSMUSG00000057788	0.030204116	NS		-0.161102634	0.020705098	0.056777985
EIF5<28132>	ENSG00000100664	eukaryotic translation initiation factor 5	ENSMUSG00000021282	0.014017772	NS		-0.160692792	0.021034963	0.057402541
GTPBP4<34025>	ENSG00000107937	GTP binding protein 4	ENSMUSG00000021149	0.039175945	NS		-0.160482723	0.021205795	0.057402541
NUFIP2<32609>	ENSG00000108256	nuclear fragile X mental retardation protein interacting protein 2	ENSMUSG00000037857	0.030492537	NS		-0.160475172	0.021211958	0.057402541
PNO1<23926>	ENSG00000115946	partner of NOB1 homolog (S. cerevisiae)	ENSMUSG00000020116	0.017046868		0.000184486	0.160011097	0.021593703	0.058180406
MRPS28<26279>	ENSG00000147586	mitochondrial ribosomal protein S28	ENSMUSG00000040269	0.013465575	NS		0.159628029	0.021913272	0.058784729
GSPT1<28757>	ENSG00000103342	G1 to S phase transition 1	ENSMUSG00000062203	0.011404799		3.12E-15	0.159203771	0.022271961	0.059488309
MAP4<5267>	ENSG00000047849	microtubule-associated protein 4	ENSMUSG00000032479	0.015695595	NS		0.157974247	0.023340227	0.062072939
PA2G4<27059>	ENSG00000170515	proliferation-associated 2G4, 38kDa	ENSMUSG00000025364	0.018989637	NS		-0.156873786	0.024333513	0.064223003
C14orf93<2183>	ENSG00000100802	chromosome 14 open reading frame 93	ENSMUSG00000022179	0.006465529	NS		0.156848385	0.024356863	0.064223003
ARF1<29466>	ENSG00000143761	ADP-ribosylation factor 1	ENSMUSG00000048076	0.004697471	NS		-0.156664403	0.024526567	0.064395292
ARF1<29466>	ENSG00000143761	microRNA 3620	ENSMUSG00000048076	0.004697471	NS		-0.156664403	0.024526567	0.064395292
SRSF8<8572>	ENSG00000180771		NA	0.033872131	NS		0.156540146	0.024641754	0.064423558
RSL1D1<38788>	ENSG00000171490	ribosomal L1 domain containing 1	ENSMUSG00000005846	0.00907489	NS		0.155850899	0.025289179	0.065837232
QKI<31983>	ENSG00000112531	QKI, KH domain containing, RNA binding	ENSMUSG00000062078	0.007444377	NS		0.155684079	0.025448055	0.065972492
GIGYF2<11689>	ENSG00000204120	GRB10 interacting GYF protein 2	ENSMUSG00000048000	0.039481642	NS		-0.154719545	0.026383565	0.068111559
SCG3<1622>	ENSG00000104112	secretogranin III	ENSMUSG00000032181	0.027869881	NS		-0.154050206	0.027049967	0.069540965
ANKHD1<37879>	ENSG00000131503	ankyrin repeat and KH domain containing 1	ENSMUSG00000024483	0.023385945	NS		-0.153751578	0.027351906	0.070025429
ANKHD1<37879>	ENSG00000131503	ANKHD1-EIF4EBP3 readthrough	ENSMUSG00000024483	0.023385945	NS		-0.153751578	0.027351906	0.070025429
ASS1<6749>	ENSG00000130707	argininosuccinate synthase 1	ENSMUSG00000046687	0.029498307		0.035193841	0.153407641	0.027703228	0.070090852
ZC3HAV1<33281>	ENSG00000105939	zinc finger CCCH-type, antiviral 1	ENSMUSG00000029826	0.022591013	NS		-0.153126175	0.027993605	0.070090852
LSM5<6797>	ENSG00000106355	LSM5 homolog, U6 small nuclear RNA associated (S. cerevisiae)	ENSMUSG00000091625	0.014514661		0.005223574	-0.153060324	0.028061916	0.070090852
UBAP2L<27246>	ENSG00000143569	ubiquitin associated protein 2-like	ENSMUSG00000042520	0.040937006	NS		-0.152969653	0.028156207	0.070090852
SUMO1<29622>	ENSG00000116030	small ubiquitin-like modifier 1	ENSMUSG00000026021	0.036714486	NS		-0.152964161	0.028161927	0.070090852
FAM208A<25782>	ENSG00000163946	family with sequence similarity 208, member A	ENSMUSG00000040651	0.025087518	NS		-0.152959355	0.028166933	0.070090852
UCHL5<39455>	ENSG00000116750	ubiquitin carboxyl-terminal hydrolase L5	ENSMUSG00000018189	0.042948536	NS		0.152953863	0.028172655	0.070090852
GSPT2<21570>	ENSG00000189369	G1 to S phase transition 2	ENSMUSG00000071723	0.033237697	NS		-0.152841277	0.028290171	0.070100542
GSPT2<21570>	ENSG00000268273	G1 to S phase transition 2	NA	0.033237697	NS		-0.152841277	0.028290171	0.070100542
RBM19<26593>	ENSG00000122965	RNA binding motif protein 19	ENSMUSG00000029594	0.043735754	NS		-0.152226172	0.028939643	0.071394348
ZC3H7A<43176>	ENSG00000122299	zinc finger CCCH-type containing 7A	ENSMUSG00000037965	0.033440762	NS		-0.152128689	0.029043734	0.071394348
RBM7<6727>	ENSG00000076053	RNA binding motif protein 7	ENSMUSG00000042396	0.038932737	NS		0.151680403	0.02952653	0.072293131
C7orf50<39667>	ENSG00000146540	uncharacterized LOC101930223	ENSMUSG00000053553	0.011742075	NS		-0.151417473	0.029812878	0.072705719
C7orf50<39667>	ENSG00000146540	chromosome 7 open reading frame 50	ENSMUSG00000053553	0.011742075	NS		-0.151417473	0.029812878	0.072705719
MSI1<20126>	ENSG00000135097	musashi RNA-binding protein 1	ENSMUSG00000054256	0.00458779	NS		-0.151076282	0.030187986	0.073330669
PWP2<12561>	ENSG00000241945	PWP2 periodic tryptophan protein homolog (yeast)	ENSMUSG00000032834	0.001699947		0.000959522	0.150750194	0.030550246	0.073919625
RBMXL2<30445>	ENSG00000170748	RNA binding motif protein, X-linked-like 2	ENSMUSG00000073894	0.032428721	NS		-0.150492756	0.030838852	0.074326447
COL14A1<6076>	ENSG00000187955	collagen, type XIV, alpha 1	ENSMUSG00000022371 NS			0.045115041	-0.150084288	0.03130154	0.075148055
ASS1<20878>	ENSG00000130707	argininosuccinate synthase 1	ENSMUSG00000046687	0.009724957		0.018839627	0.149541952	0.031924992	0.076347747
MRPL4<39288>	ENSG00000105364	mitochondrial ribosomal protein L4	ENSMUSG00000003299 NS			0.000108504	0.148808768	0.03278461	0.078100789
SERBP1<24992>	ENSG00000142864	SERPINE1 mRNA binding protein 1	ENSMUSG00000036371	0.042299249	NS		-0.14857879	0.033058268	0.078222905
EIF4A2<24898>	ENSG00000156976	small nucleolar RNA, H/ACA box 81	ENSMUSG00000022884	0.015426922	NS		0.148552703	0.033089432	0.078222905
EIF4A2<24898>	ENSG00000156976	small nucleolar RNA, H/ACA box 63	ENSMUSG00000022884	0.015426922	NS		0.148552703	0.033089432	0.078222905
EIF4A2<24898>	ENSG00000156976	microRNA 1248	ENSMUSG00000022884	0.015426922	NS		0.148552703	0.033089432	0.078222905
EIF4A2<24898>	ENSG00000156976	small nucleolar RNA, C/D box 2	ENSMUSG00000022884	0.015426922	NS		0.148552703	0.033089432	0.078222905
EIF4A2<24898>	ENSG00000156976	eukaryotic translation initiation factor 4A2	ENSMUSG00000022884	0.015426922	NS		0.148552703	0.033089432	0.078222905
EIF4A2<24898>	ENSG00000156976	small nucleolar RNA, H/ACA box 4	ENSMUSG00000022884	0.015426922	NS		0.148552703	0.033089432	0.078222905
MTDH<31325>	ENSG00000147649	metadherin	ENSMUSG00000022255	0.047789726	NS		0.147885424	0.033895099	0.079821667

BICC1<16734>	ENSG00000122870	bicaudal C homolog 1 (Drosophila)	ENSMUSG00000014329	0.037846319	0.014676774	-0.147695263	0.034127724	0.080063891
TLR2<9956>	ENSG00000137462	toll-like receptor 2	ENSMUSG00000027995	NS	0.043457201	0.147342402	0.03456297	0.08077785
RPL12<26243>	ENSG00000197958	ribosomal protein L12	ENSMUSG00000069682	0.027619325	NS	-0.147032103	0.034949596	0.08137322
PQBP1<23085>	ENSG00000268142	polyglutamine binding protein 1	NA	NS	0.008922809	0.14660304	0.035490235	0.082073386
PQBP1<23085>	ENSG00000102103	polyglutamine binding protein 1	ENSMUSG00000031157	NS	0.008922809	0.14660304	0.035490235	0.082073386
SUMO1<39462>	ENSG00000116030	small ubiquitin-like modifier 1	ENSMUSG00000026021	NS	0.0042614	-0.146582445	0.035516363	0.082073386
NOL8<1508>	ENSG00000198000	nucleolar protein 8	ENSMUSG00000021392	0.004896623	0.000773044	-0.146237135	0.035956871	0.082781301
OASL<36193>	ENSG00000135114	2'-5'-oligoadenylate synthetase-like	ENSMUSG00000041827	0.025963856	NS	0.145912419	0.036375317	0.083433355
RBPMS<40572>	ENSG00000157110	RNA binding protein with multiple splicing	ENSMUSG00000031586	NS	0.045604064	0.145410587	0.037030105	0.084322772
YWHAZ<958>	ENSG00000164924	tyrosine 3-monooxygenase/tryptophan 5-monooxygenase activation protein, ze	ENSMUSG00000022285	0.034232794	NS	-0.145405781	0.037036423	0.084322772
HNRNPK<17917>	ENSG00000165119	heterogeneous nuclear ribonucleoprotein K	ENSMUSG00000021546	0.025481908	NS	0.144862759	0.037756254	0.085645611
KPNB1<26671>	ENSG00000108424	karyopherin (importin) beta 1	ENSMUSG00000001440	0.023111493	NS	-0.144483124	0.038266486	0.086399523
UBE2N<10112>	ENSG00000177889	ubiquitin-conjugating enzyme E2N	ENSMUSG00000074781	0.019728374	NS	0.144407609	0.03836867	0.086399523
RBM39<32363>	ENSG00000131051	RNA binding motif protein 39	ENSMUSG00000027620	0.046234704	NS	-0.144200972	0.038649459	0.086715334
SYNCRIP<29997>	ENSG00000135316	synaptotagmin binding, cytoplasmic RNA interacting protein	ENSMUSG00000032423	0.018166893	NS	-0.143990902	0.038936687	0.087043253
YTHDF2<12433>	ENSG00000198492	YTH domain family, member 2	ENSMUSG00000040025	0.019543361	NS	-0.143572136	0.039514638	0.088016364
PABPC1<32256>	ENSG00000070756	poly(A) binding protein, cytoplasmic 1	ENSMUSG00000022283	0.012188263	0.004824593	0.143329115	0.039853342	0.088451478
GRB2<12737>	ENSG00000177885	growth factor receptor-bound protein 2	ENSMUSG00000059923	0.012653952	NS	0.142987237	0.04033396	0.089154847
HOXB6<35349>	ENSG00000108511	homeobox B6	ENSMUSG00000000690	0.031813806	NS	0.142898679	0.04045925	0.089154847
UBE2D1<42966>	ENSG00000072401	ubiquitin-conjugating enzyme E2D 1	ENSMUSG00000019927	0.040743058	NS	0.142589753	0.040898876	0.089794067
HNRNPA1L2<12396>	ENSG00000139675	heterogeneous nuclear ribonucleoprotein A1-like 2	NA	0.010589841	NS	-0.142490897	0.041040402	0.089794067
RBM27<36607>	ENSG00000091009	RNA binding motif protein 27	ENSMUSG00000024491	0.00205677	NS	-0.142278082	0.041346474	0.090144071
SETD1B<39229>	ENSG00000139718	SET domain containing 1B	ENSMUSG00000038384	NS	0.043021576	-0.142107143	0.041593707	0.0903638
ANP32A<41200>	ENSG00000140350	acidic (leucine-rich) nuclear phosphoprotein 32 family, member A	ENSMUSG00000032249	0.021635331	NS	0.141923847	0.041860191	0.090623639
JUN<330>	ENSG00000177606	jun proto-oncogene	ENSMUSG00000052684	0.018139682	NS	-0.141030022	0.043180314	0.093154725
RBM4B<43064>	ENSG00000173914	RNA binding motif protein 4B	ENSMUSG00000033760	0.04917562	NS	-0.140910571	0.043359353	0.093215049
MRPL43<43763>	ENSG00000055950	mitochondrial ribosomal protein L43	ENSMUSG00000025208	NS	0.038351666	-0.140554277	0.043897086	0.094043419
EIF5B<17050>	ENSG00000158417	eukaryotic translation initiation factor 5B	ENSMUSG00000026083	0.024771819	NS	0.140437572	0.044074432	0.09409662
PPIA<27259>	ENSG00000196262	peptidylprolyl isomerase A (cyclophilin A)	ENSMUSG00000055795	0.015149618	NS	-0.140202788	0.044433031	0.094535102
PPIA<27259>	ENSG00000196262	peptidyl-prolyl cis-trans isomerase A-like	ENSMUSG00000055795	0.015149618	NS	-0.140202788	0.044433031	0.094535102
PRR3<41020>	ENSG00000204576	proline rich 3	ENSMUSG00000038500	0.020379852	0.045700965	0.139763427	0.04511066	0.095647001
PRR3<41020>	ENSG00000223887	proline rich 3	NA	0.020379852	0.045700965	0.139763427	0.04511066	0.095647001
PRR3<41020>	ENSG00000229202	proline rich 3	NA	0.020379852	0.045700965	0.139763427	0.04511066	0.095647001
PRR3<41020>	ENSG00000223766	proline rich 3	NA	0.020379852	0.045700965	0.139763427	0.04511066	0.095647001
PRR3<41020>	ENSG00000233564	proline rich 3	NA	0.020379852	0.045700965	0.139763427	0.04511066	0.095647001
PRR3<41020>	ENSG00000228186	proline rich 3	NA	0.020379852	0.045700965	0.139763427	0.04511066	0.095647001
PRR3<41020>	ENSG00000206491	proline rich 3	NA	0.020379852	0.045700965	0.139763427	0.04511066	0.095647001
POP1<2404>	ENSG00000104356	processing of precursor 1, ribonuclease P/MRP subunit (S. cerevisiae)	ENSMUSG00000022325	0.03566184	NS	-0.138793401	0.046637402	0.098315068
LSM14B<26211>	ENSG00000149657	LSM14B, SCD6 homolog B (S. cerevisiae)	ENSMUSG00000039108	0.013684208	NS	0.138724751	0.046747068	0.098315068
MRPL3<25858>	ENSG00000114686	mitochondrial ribosomal protein L3	ENSMUSG00000032563	0.039651418	NS	-0.138662279	0.046847051	0.098315068
RNPS1<3331>	ENSG00000205937	RNA binding protein S1, serine-rich domain	ENSMUSG00000096403	0.01698503	NS	-0.138386992	0.047289766	0.098693798
TEP1<16110>	ENSG00000129566	telomerase-associated protein 1	ENSMUSG00000006281	0.027894587	NS	0.138351294	0.04734743	0.098693798
ANXA7<36316>	ENSG00000138279	annexin A7	ENSMUSG00000021814	0.049693648	NS	0.138103467	0.047749375	0.098907102
NAT10<36045>	ENSG00000135372	N-acetyltransferase 10 (GCN5-related)	ENSMUSG00000027185	0.040071392	NS	0.138076694	0.047792968	0.098907102
RPL31<29385>	ENSG00000071082	ribosomal protein L31	ENSMUSG00000073702	NS	0.017164092	-0.137992254	0.047930672	0.098907102
ZCCHC7<13520>	ENSG00000147905	zinc finger, CCHC domain containing 7	ENSMUSG00000035649	0.017002782	NS	0.137813077	0.048223969	0.099180632
HSPA8<21681>	ENSG00000109971	small nucleolar RNA, C/D box 14D	ENSMUSG00000015656	NS	0.04266121	0.137637333	0.0485131	0.099443796
HSPA8<21681>	ENSG00000109971	heat shock 70kDa protein 8	ENSMUSG00000015656	NS	0.04266121	0.137637333	0.0485131	0.099443796
HSPA8<21681>	ENSG00000109971	small nucleolar RNA, C/D box 14C	ENSMUSG00000015656	NS	0.04266121	0.137637333	0.0485131	0.099443796
LARP1<26387>	ENSG00000155506	La ribonucleoprotein domain family, member 1	ENSMUSG00000037331	0.029363064	NS	0.13692955	0.049692199	0.101523468
NLRP11<35794>	ENSG00000179873	NLR family, pyrin domain containing 11	NA	0.01011827	0.000978547	0.136524514	0.050377617	0.10258413
OTUD4<34900>	ENSG00000164164	OTU domain containing 4	ENSMUSG00000036990	0.039522645	NS	-0.136289731	0.050778515	0.103060351
SNIP1<38133>	ENSG00000163877	Smad nuclear interacting protein 1	ENSMUSG00000050213	0.032580044	NS	-0.13605632	0.051179694	0.103533996
UCHL5<36386>	ENSG00000116750	ubiquitin carboxyl-terminal hydrolase L5	ENSMUSG00000018189	0.049379661	NS	0.135864786	0.051510861	0.103838829
RPL21<17668>	ENSG00000122026	small nucleolar RNA, C/D box 102	ENSMUSG00000059912	NS	0.048680368	0.135774855	0.051666969	0.103838829
RPL21<17668>	ENSG00000122026	small nucleolar RNA, H/ACA box 27	ENSMUSG00000059912	NS	0.048680368	0.135774855	0.051666969	0.103838829
RPL21<17668>	ENSG00000122026	ribosomal protein L21	ENSMUSG00000059912	NS	0.048680368	0.135774855	0.051666969	0.103838829
DIMT1<25500>	ENSG00000086189	DIM1 dimethyladenosine transferase 1 homolog (S. cerevisiae)	ENSMUSG00000021692	0.024921282	0.028149516	0.135282633	0.052528366	0.105227287
HNRNPA1<7486>	ENSG00000135486	heterogeneous nuclear ribonucleoprotein A1	ENSMUSG00000046636	0.026126265	NS	0.135109635	0.052833933	0.105496876
KRT18<10754>	ENSG00000111057	keratin 18	ENSMUSG00000023043	0.042026495	NS	-0.134710778	0.053544056	0.106569952
UCHL5<11285>	ENSG00000116750	ubiquitin carboxyl-terminal hydrolase L5	ENSMUSG00000018189	NS	0.009013052	0.132534569	0.057559244	0.11419309
NPM1<25633>	LRG_458	nucleophosmin (nucleolar phosphoprotein B23, numatrin)	NA	0.020643999	0.008362429	0.131732735	0.059099946	0.11687394
NPM1<25633>	ENSG00000181163	nucleophosmin (nucleolar phosphoprotein B23, numatrin)	ENSMUSG00000090602	0.020643999	0.008362429	0.131732735	0.059099946	0.11687394
RPL35<13304>	ENSG00000136942	ribosomal protein L35	ENSMUSG00000078193	0.029158648	NS	-0.13120001	0.060142229	0.118555131
UBE2N<27016>	ENSG00000177889	ubiquitin-conjugating enzyme E2N	ENSMUSG00000074781	NS	0.036157482	-0.131031131	0.060475785	0.118832982
RPL13<14760>	ENSG00000167526	ribosomal protein L13	ENSMUSG00000059835	0.040996609	0.000279456	-0.130908933	0.060718086	0.118930354
RPL13<14760>	ENSG00000167526	small nucleolar RNA, C/D box 68	ENSMUSG00000059835	0.040996609	0.000279456	-0.130908933	0.060718086	0.118930354
RPL3<8953>	ENSG00000100316	RNA, U86 small nucleolar	ENSMUSG00000060036	0.034951373	NS	0.13065836	0.061217437	0.119528989
RPL3<8953>	ENSG00000100316	small nucleolar RNA, C/D box 83B	ENSMUSG00000060036	0.034951373	NS	0.13065836	0.061217437	0.119528989
RPL3<8953>	ENSG00000100316	ribosomal protein L3	ENSMUSG00000060036	0.034951373	NS	0.13065836	0.061217437	0.119528989

SMG1<8822>	ENSG00000157106	SMG1 phosphatidylinositol 3-kinase-related kinase	ENSMUSG00000030655		0.019737027	NS		-0.13041328	0.061709104	0.120108879
SMG1<8822>	ENSG00000157106	bolA family member 2	ENSMUSG00000030655		0.019737027	NS		-0.13041328	0.061709104	0.120108879
SMG1<8822>	ENSG00000157106	serine/threonine-protein kinase SMG1-like	ENSMUSG00000030655		0.019737027	NS		-0.13041328	0.061709104	0.120108879
GCN1L1<27279>	ENSG00000089154	GCN1 general control of amino-acid synthesis 1-like 1 (yeast)	ENSMUSG00000041638	NS			0.012676895	-0.130280785	0.061976255	0.120249536
SAMD14<20550>	ENSG00000167100	sterile alpha motif domain containing 14	ENSMUSG00000047181		0.040035254	NS		0.130127008	0.062287508	0.120474588
EIF2AK2<39258>	ENSG00000055332	eukaryotic translation initiation factor 2-alpha kinase 2	ENSMUSG00000024079		0.029054589	NS		0.129454237	0.063664364	0.122752844
RPS15<6027>	ENSG00000115268	ribosomal protein S15	ENSMUSG00000063457		0.041270592	NS		0.12909863	0.064402153	0.123788556
SUPT16H<17678>	ENSG00000092201	suppressor of Ty 16 homolog (S. cerevisiae)	ENSMUSG00000035726	NS			0.008972563	0.127485351	0.067837745	0.129987241
ATP5C1<18266>	ENSG00000165629	ATP synthase, H+ transporting, mitochondrial F1 complex, gamma polypeptide 1	ENSMUSG00000025781	NS			0.014549792	0.126986952	0.068928885	0.131300171
NOC3L<16225>	ENSG00000173145	nucleolar complex associated 3 homolog (S. cerevisiae)	ENSMUSG00000024999		0.015264775	NS		-0.126978027	0.068948553	0.131300171
PPIA<27567>	ENSG00000196262	peptidylprolyl isomerase A (cyclophilin A)	ENSMUSG00000055795		0.020604613	NS		0.126598392	0.069789446	0.132492587
PPIA<27567>	ENSG00000196262	peptidyl-prolyl cis-trans isomerase A-like	ENSMUSG00000055795		0.020604613	NS		0.126598392	0.069789446	0.132492587
HSP90AA1<39893>	ENSG00000080824	heat shock protein 90kDa alpha (cytosolic), class A member 1	ENSMUSG00000021270		0.020949207	NS		0.126457659	0.070103285	0.13260464
RNPS1<19170>	ENSG00000205937	RNA binding protein S1, serine-rich domain	ENSMUSG00000096403		0.008778282	NS		-0.126379398	0.070278307	0.13260464
SLC25A11<42168>	ENSG00000108528	solute carrier family 25 (mitochondrial carrier; oxoglutarate carrier), member 11	ENSMUSG00000014606	NS			0.037630941	0.126130198	0.070837986	0.13325317
RSL1D1<40057>	ENSG00000171490	ribosomal L1 domain containing 1	ENSMUSG00000005846		0.041547105	NS		0.125517839	0.072228705	0.135456274
SYNJ2<18187>	ENSG00000078269	synaptojanin 2	ENSMUSG00000023805		0.023771829	NS		0.125309142	0.072707713	0.135930103
EIF5<22340>	ENSG00000100664	eukaryotic translation initiation factor 5	ENSMUSG00000021282	NS			0.020825404	0.125211659	0.072932342	0.135930103
DDX46<38751>	ENSG00000145833	DEAD (Asp-Glu-Ala-Asp) box polypeptide 46	ENSMUSG00000021500		0.042998508	NS		0.125048958	0.073308507	0.135930103
GLTSCR2<34298>	ENSG00000105373	small nucleolar RNA, C/D box 23	ENSMUSG00000041560	NS			1.44E-05	0.125025617	0.073362601	0.135930103
GLTSCR2<34298>	ENSG00000105373	glioma tumor suppressor candidate region gene 2	ENSMUSG00000041560	NS			1.44E-05	0.125025617	0.073362601	0.135930103
YTHDF3<34740>	ENSG00000185728	YTH domain family, member 3	ENSMUSG00000047213		0.028267075	NS		0.124894496	0.073667082	0.136085594
SRRT<16739>	ENSG00000087087	serrate RNA effector molecule homolog (Arabidopsis)	ENSMUSG00000037364		0.048697432	NS		0.124148269	0.0754195	0.13890696
DIS3<17655>	ENSG00000083520	DIS3 mitotic control homolog (S. cerevisiae)	ENSMUSG00000033166	NS			0.0441495	-0.12393614	0.075923777	0.13941956
EXOSC4<21235>	ENSG00000178896	exosome component 4	ENSMUSG00000034259	NS			0.042382545	-0.123567489	0.076806635	0.140622228
EXOSC4<21235>	ENSG00000260189	exosome component 4	NA	NS			0.042382545	-0.123567489	0.076806635	0.140622228
CAPRIN1<31471>	ENSG00000135387	cell cycle associated protein 1	ENSMUSG00000027184	NS			0.02533798	0.123056732	0.078043521	0.142464059
PAK1IP1<43933>	ENSG00000111845	PAK1 interacting protein 1	ENSMUSG00000038683		0.024104774		0.000636759	0.122953756	0.078294832	0.14250121
LLPH<410>	ENSG00000139233	LLP homolog, long-term synaptic facilitation (Aplysia)	ENSMUSG00000020224	NS			0.016387747	-0.122519201	0.079362578	0.144019741
TRA2B<41618>	ENSG00000136527	transformer 2 beta homolog (Drosophila)	ENSMUSG00000022858		0.043778839	NS		-0.121698832	0.081410328	0.147086306
PAK1IP1<36080>	ENSG00000111845	PAK1 interacting protein 1	ENSMUSG00000038683		0.026416144		0.017147894	-0.121601349	0.081656463	0.147086306
TFB2M<29176>	ENSG00000162851	transcription factor B2, mitochondrial	ENSMUSG00000091277		0.0467731	NS		0.121557413	0.081767593	0.147086306
GRB2<11181>	ENSG00000177885	growth factor receptor-bound protein 2	ENSMUSG00000059923		0.034559587	NS		-0.120674572	0.084026529	0.150710375
CIRBP<7062>	ENSG00000099622	cold inducible RNA binding protein	ENSMUSG00000045193		0.046450899	NS		0.120547569	0.08435558	0.150862008
DDX3X<22376>	ENSG00000215301	DEAD (Asp-Glu-Ala-Asp) box helicase 3, X-linked	ENSMUSG00000039224		0.012268977	NS		-0.119605003	0.08683013	0.1548387
HNRNPA3<15461>	ENSG00000170144	heterogeneous nuclear ribonucleoprotein A3	ENSMUSG00000099115		0.017190035		0.007647065	0.11943887	0.087272256	0.155178629
TEX10<21003>	ENSG00000136891	testis expressed 10	ENSMUSG00000028345		0.024324927	NS		0.11856564	0.089625886	0.158905673
S100A4<18201>	ENSG00000196154	S100 calcium binding protein A4	ENSMUSG00000001020		0.041587857	NS		-0.117858544	0.091568675	0.16188503
CGGBP1<44698>	ENSG00000163320	CGG triplet repeat binding protein 1	ENSMUSG00000054604		0.048505477	NS		0.117621014	0.092228789	0.162586181
QKI<4437>	ENSG00000112531	QKI, KH domain containing, RNA binding	ENSMUSG00000062078		0.032414039	NS		-0.117229022	0.093326458	0.164052495
RBM41<28727>	ENSG00000089682	RNA binding motif protein 41	ENSMUSG00000031433	NS			0.039877987	-0.117062889	0.093794799	0.164407363
PATL1<6883>	ENSG00000166889	protein associated with topoisomerase II homolog 1 (yeast)	ENSMUSG00000046139		0.040244531	NS		0.116653047	0.09495817	0.165975045
HLA-A<28178>	ENSG00000223980	major histocompatibility complex, class I, A	NA	NS			0.020766932	0.116210254	0.096227945	0.167719319
HLA-A<28178>	ENSG00000235657	major histocompatibility complex, class I, A	NA	NS			0.020766932	0.116210254	0.096227945	0.167719319
HLA-A<28178>	ENSG00000229215	major histocompatibility complex, class I, A	NA	NS			0.020766932	0.116210254	0.096227945	0.167719319
HLA-A<28178>	ENSG00000227715	major histocompatibility complex, class I, A	NA	NS			0.020766932	0.116210254	0.096227945	0.167719319
HLA-A<28178>	ENSG00000231834	major histocompatibility complex, class I, A	NA	NS			0.020766932	0.116210254	0.096227945	0.167719319
HLA-A<28178>	ENSG00000224320	major histocompatibility complex, class I, A	NA	NS			0.020766932	0.116210254	0.096227945	0.167719319
HLA-A<28178>	ENSG00000206505	major histocompatibility complex, class I, A	NA	NS			0.020766932	0.116210254	0.096227945	0.167719319
HLA-A<28178>	ENSG00000206503	major histocompatibility complex, class I, A	NA	NS			0.020766932	0.116210254	0.096227945	0.167719319
C14orf93<31609>	ENSG00000100802	chromosome 14 open reading frame 93	ENSMUSG00000022179		0.02539019	NS		-0.115901328	0.097121794	0.168800407
DAZ4<31208>	ENSG00000205916	deleted in azoospermia 1	ENSMUSG00000010592	NS			0.001325577	-0.115286223	0.098921161	0.170748666
DAZ4<31208>	ENSG00000205916	deleted in azoospermia 4	ENSMUSG00000010592	NS			0.001325577	-0.115286223	0.098921161	0.170748666
DAZ4<31208>	ENSG00000205916	deleted in azoospermia 2	ENSMUSG00000010592	NS			0.001325577	-0.115286223	0.098921161	0.170748666
DAZ2<31208>	ENSG00000205944	deleted in azoospermia 2	ENSMUSG00000010592	NS			0.001325577	-0.115286223	0.098921161	0.170748666
DAZ2<31208>	ENSG00000205944	deleted in azoospermia 1	ENSMUSG00000010592	NS			0.001325577	-0.115286223	0.098921161	0.170748666
DAZ2<31208>	ENSG00000205944	deleted in azoospermia 4	ENSMUSG00000010592	NS			0.001325577	-0.115286223	0.098921161	0.170748666
PEG10<36613>	ENSG00000242265	paternally expressed 10	ENSMUSG00000092035	NS			0.026172766	-0.115234736	0.099072968	0.170748666
TOP2A<21075>	ENSG00000131747	topoisomerase (DNA) II alpha 170kDa	ENSMUSG00000020914	NS			0.01162091	0.114480271	0.101318677	0.174132696
MCAT<9818>	ENSG00000100294	malonyl CoA:ACP acyltransferase (mitochondrial)	ENSMUSG00000048755		0.034536409		0.025727326	0.113561046	0.104108976	0.178431258
HNRNPF<25648>	ENSG00000169813	heterogeneous nuclear ribonucleoprotein F	ENSMUSG00000091223		0.038759829	NS		-0.11334823	0.104763535	0.179055622
RPL35A<1293>	ENSG00000182899	ribosomal protein L35a	ENSMUSG00000060636		0.039570276	NS		-0.113036559	0.105727996	0.180204906
UBE2I<138>	ENSG00000103275	ubiquitin-conjugating enzyme E2I	ENSMUSG00000015120	NS			0.038490045	-0.111802916	0.109614236	0.185014595
POLR2A<29099>	ENSG00000181222	polymerase (RNA) II (DNA directed) polypeptide A, 220kDa	ENSMUSG00000005198		0.047593349		0.029306265	-0.111767218	0.109728339	0.185014595
UBE2D2<29816>	ENSG00000131508	ubiquitin-conjugating enzyme E2D 2	ENSMUSG00000091896	NS			0.039112477	-0.111677972	0.110014004	0.185014595
UBE2D3<29816>	ENSG00000109332	ubiquitin-conjugating enzyme E2D 3	ENSMUSG00000078578	NS			0.039112477	-0.111677972	0.110014004	0.185014595
CELF4<6445>	ENSG00000101489	CUGBP, Elav-like family member 4	ENSMUSG00000024268	NS			0.043705141	0.111666988	0.110049203	0.185014595
DARS<19669>	ENSG00000115866	aspartyl-tRNA synthetase	ENSMUSG00000026356		0.021768814		0.015801798	0.111056689	0.112018869	0.187814297
UTP6<29540>	ENSG00000108651	UTP6, small subunit (SSU) processome component, homolog (yeast)	ENSMUSG00000035575	NS			0.004517536	-0.110952341	0.112358386	0.187872989
CCDC137<17423>	ENSG00000185298	coiled-coil domain containing 137	ENSMUSG00000049957	NS			0.003764772	-0.110678427	0.11325345	0.188734479

TNPO1<35162>	ENSG00000083312	transportin 1	ENSMUSG00000009470	NS	0.045285347	-0.110607717	0.11348541	0.188734479
SYNE1<25646>	ENSG00000131018	spectrin repeat containing, nuclear envelope 1	ENSMUSG00000019769	NS	0.002070345	0.11001252	0.115452676	0.191490097
RBM8A<26903>	ENSG00000131795	RNA binding motif protein 8A	ENSMUSG00000078184	0.046122799	NS	-0.109084371	0.118573424	0.196138841
HNRNPU<23354>	ENSG00000153187	heterogeneous nuclear ribonucleoprotein U (scaffold attachment factor A)	ENSMUSG00000039630	0.03775669	NS	-0.108814576	0.119492773	0.19713117
DDX54<43404>	ENSG00000123064	DEAD (Asp-Glu-Ala-Asp) box polypeptide 54	ENSMUSG00000029599	0.045080846	0.019290782	-0.107699011	0.123353111	0.202956967
RPL4<3503>	ENSG00000174444	small nucleolar RNA, C/D box 18B	ENSMUSG00000032399	NS	0.000974827	0.107603587	0.123687754	0.202966416
RPL4<3503>	ENSG00000174444	ribosomal protein L4	ENSMUSG00000032399	NS	0.000974827	0.107603587	0.123687754	0.202966416
RPL4<3503>	ENSG00000174444	small nucleolar RNA, C/D box 18C	ENSMUSG00000032399	NS	0.000974827	0.107603587	0.123687754	0.202966416
RPL4<3503>	ENSG00000174444	small nucleolar RNA, C/D box 18A	ENSMUSG00000032399	NS	0.000974827	0.107603587	0.123687754	0.202966416
RPL4<3503>	ENSG00000174444	small nucleolar RNA, C/D box 16	ENSMUSG00000032399	NS	0.000974827	0.107603587	0.123687754	0.202966416
RPS10<2348>	ENSG00000124614	ribosomal protein S10	ENSMUSG00000052146	0.046705674	NS	0.107346149	0.124594078	0.203911299
SMG1<26967>	ENSG00000157106	SMG1 phosphatidylinositol 3-kinase-related kinase	ENSMUSG00000030655	NS	0.031212281	-0.107089398	0.125503098	0.204855589
SMG1<26967>	ENSG00000157106	bolA family member 2	ENSMUSG00000030655	NS	0.031212281	-0.107089398	0.125503098	0.204855589
SMG1<26967>	ENSG00000157106	serine/threonine-protein kinase SMG1-like	ENSMUSG00000030655	NS	0.031212281	-0.107089398	0.125503098	0.204855589
RPS4X<41024>	ENSG00000198034	ribosomal protein S4, X-linked	ENSMUSG00000031320	NS	0.001339499	0.10691228	0.12613316	0.205340856
TRMT10C<5642>	ENSG00000174173	tRNA methyltransferase 10 homolog C (S. cerevisiae)	ENSMUSG00000044763	NS	0.01771912	0.106246374	0.128523896	0.208682227
RC3H2<22959>	ENSG00000056586	ring finger and CCCH-type domains 2	ENSMUSG00000075376	NS	0.030976587	0.105965595	0.129542366	0.209783887
DROSHA<12827>	ENSG00000113360	drosha, ribonuclease type III	ENSMUSG00000022191	0.040569229	NS	0.105768569	0.130260743	0.2103949
ZC3H13<40371>	ENSG00000123200	zinc finger CCCH-type containing 13	ENSMUSG00000022000	NS	0.00753152	0.10565667	0.130670106	0.210505096
CAPRIN1<26963>	ENSG00000135387	cell cycle associated protein 1	ENSMUSG00000027184	0.032057847	NS	0.104885042	0.133519968	0.213792418
SYF2<21929>	ENSG00000117614	SYF2 pre-mRNA-splicing factor	ENSMUSG00000028821	NS	0.000588531	-0.104862388	0.133604354	0.213792418
CDK11B<8250>	ENSG00000248333	cyclin-dependent kinase 11B	ENSMUSG00000029062	NS	0.013364906	0.104823257	0.133750208	0.213792418
CDK11B<8250>	ENSG00000248333	cyclin-dependent kinase 11A	ENSMUSG00000029062	NS	0.013364906	0.104823257	0.133750208	0.213792418
JUN<33732>	ENSG00000177606	jun proto-oncogene	ENSMUSG00000052684	NS	0.010824178	0.104515018	0.134903408	0.215078547
CSNK1E<8594>	ENSG00000213923	casein kinase 1, epsilon	ENSMUSG00000022433	0.03727112	NS	-0.104374972	0.135429869	0.215361465
TLR2<8451>	ENSG00000137462	toll-like receptor 2	ENSMUSG00000027995	0.034199387	NS	0.104243163	0.1359268	0.215595978
SDAD1<34383>	ENSG00000198301	SDA1 domain containing 1	ENSMUSG00000029415	NS	0.03191425	-0.103409751	0.139101266	0.219923259
CLASRP<37003>	ENSG00000104859	CLK4-associating serine/arginine rich protein	ENSMUSG00000061028	0.035737623	NS	-0.103340414	0.139367901	0.219923259
ATP5J2-PTCD1<30345>	ENSG00000248919	ATP5J2-PTCD1 readthrough	ENSMUSG00000029624	0.029636636	NS	-0.102510434	0.142589993	0.223862672
ATP5J2-PTCD1<30345>	ENSG00000248919	pentatricopeptide repeat domain 1	ENSMUSG00000029624	0.029636636	NS	-0.102510434	0.142589993	0.223862672
PTCD1<30345>	ENSG00000106246	ATP5J2-PTCD1 readthrough	ENSMUSG00000029624	0.029636636	NS	-0.102510434	0.142589993	0.223862672
PTCD1<30345>	ENSG00000106246	pentatricopeptide repeat domain 1	ENSMUSG00000029624	0.029636636	NS	-0.102510434	0.142589993	0.223862672
ESRP2<27803>	ENSG00000103067	epithelial splicing regulatory protein 2	ENSMUSG00000084128	0.041578008	0.010272431	-0.102408832	0.142988294	0.223918226
HSP90AB1<1935>	ENSG00000096384	heat shock protein 90kDa alpha (cytosolic), class B member 1	ENSMUSG00000023944	0.025155212	NS	0.102071073	0.144318468	0.225429151
DDX53<24609>	ENSG00000184735	DEAD (Asp-Glu-Ala-Asp) box polypeptide 53	NA	0.042570247	NS	-0.101401734	0.146982271	0.228889017
HSP90AA1<34517>	ENSG00000080824	heat shock protein 90kDa alpha (cytosolic), class A member 1	ENSMUSG00000021270	NS	0.040316509	-0.101263061	0.147538794	0.228889017
LSM4<8944>	ENSG00000130520	LSM4 homolog, U6 small nuclear RNA associated (S. cerevisiae)	ENSMUSG00000031848	NS	0.002030201	0.101236288	0.147646426	0.228889017
RBMY1B<16568>	ENSG00000242875	RNA binding motif protein, Y-linked, family 1, member D	ENSMUSG00000096520	0.02017569	NS	-0.100663746	0.149962364	0.231896744
RBMY1B<16568>	ENSG00000242875	RNA binding motif protein, Y-linked, family 1, member B	ENSMUSG00000096520	0.02017569	NS	-0.100663746	0.149962364	0.231896744
RPL7A<25320>	ENSG00000148303	small nucleolar RNA, C/D box 24	ENSMUSG00000078177	0.030975654	NS	-0.100386399	0.151094075	0.232926886
RPL7A<25320>	ENSG00000148303	small nucleolar RNA, C/D box 36A	ENSMUSG00000078177	0.030975654	NS	-0.100386399	0.151094075	0.232926886
RPL7A<25320>	ENSG00000148303	small nucleolar RNA, C/D box 36B	ENSMUSG00000078177	0.030975654	NS	-0.100386399	0.151094075	0.232926886
RPL7A<25320>	ENSG00000260501	small nucleolar RNA, C/D box 36B	NA	0.030975654	NS	-0.100386399	0.151094075	0.232926886
RPL7A<25320>	ENSG00000148303	ribosomal protein L7a	ENSMUSG00000078177	0.030975654	NS	-0.100386399	0.151094075	0.232926886
RPL7A<25320>	ENSG00000260501	small nucleolar RNA, C/D box 24	NA	0.030975654	NS	-0.100386399	0.151094075	0.232926886
RPL7A<25320>	ENSG00000260501	ribosomal protein L7a	NA	0.030975654	NS	-0.100386399	0.151094075	0.232926886
RPL7A<25320>	ENSG00000260501	small nucleolar RNA, C/D box 36A	NA	0.030975654	NS	-0.100386399	0.151094075	0.232926886
IMP4<44009>	ENSG00000136718	IMP4, U3 small nucleolar ribonucleoprotein, homolog (yeast)	ENSMUSG00000026127	0.045699258	0.026035834	-0.100315689	0.151383636	0.232926886
TLR2<10722>	ENSG00000137462	toll-like receptor 2	ENSMUSG00000027995	0.019241275	NS	-0.099843376	0.153328562	0.235332702
GNL3<3011>	ENSG00000163938	small nucleolar RNA, C/D box 19B	ENSMUSG00000042354	0.046325052	NS	-0.099743147	0.153743709	0.235384275
GNL3<3011>	ENSG00000163938	guanine nucleotide binding protein-like 3 (nucleolar)	ENSMUSG00000042354	0.046325052	NS	-0.099743147	0.153743709	0.235384275
DDX46<156>	ENSG00000145833	DEAD (Asp-Glu-Ala-Asp) box polypeptide 46	ENSMUSG00000021500	NS	0.041766315	0.099448638	0.154968466	0.236672189
RPLP0<24488>	ENSG00000089157	ribosomal protein, large, P0	ENSMUSG00000067274	0.028739762	NS	-0.099034678	0.156702394	0.238086353
IGF2BP1<8488>	ENSG00000159217	insulin-like growth factor 2 mRNA binding protein 1	ENSMUSG00000013415	0.028144783	NS	0.098959163	0.157020267	0.238086353
YWHAE<23491>	ENSG00000108953	tyrosine 3-monooxygenase/tryptophan 5-monooxygenase activation protein, ep	ENSMUSG00000020849	NS	0.008428358	0.098951611	0.157052081	0.238086353
RPL10<2621>	ENSG00000268963	small nucleolar RNA, H/ACA box 70	NA	NS	0.009248963	0.098578188	0.158631345	0.239890961
RPL10<2621>	ENSG00000147403	ribosomal protein L10	ENSMUSG00000058443	NS	0.009248963	0.098578188	0.158631345	0.239890961
RPL10<2621>	ENSG00000147403	small nucleolar RNA, H/ACA box 70	ENSMUSG00000058443	NS	0.009248963	0.098578188	0.158631345	0.239890961
RPL10<2621>	ENSG00000268963	ribosomal protein L10	NA	NS	0.009248963	0.098578188	0.158631345	0.239890961
ELAVL3<33850>	ENSG00000196361	ELAV (embryonic lethal, abnormal vision, Drosophila)-like 3 (Hu antigen C)	ENSMUSG00000003410	NS	0.008701279	0.098375637	0.159492945	0.240604204
ELAVL3<33850>	ENSG00000196361	formin-like protein 5-like	ENSMUSG00000003410	NS	0.008701279	0.098375637	0.159492945	0.240604204
YWHAG<32928>	ENSG00000170027	tyrosine 3-monooxygenase/tryptophan 5-monooxygenase activation protein, ga	ENSMUSG00000051391	0.012998811	NS	0.097831928	0.161823105	0.243524031
DDX18<32106>	ENSG00000088205	DEAD (Asp-Glu-Ala-Asp) box polypeptide 18	ENSMUSG00000001674	NS	0.017183535	0.097703552	0.162376986	0.243763039
ZC3H13<13640>	ENSG00000123200	zinc finger CCCH-type containing 13	ENSMUSG00000022000	NS	0.016073486	-0.097080209	0.165086618	0.247229204
GNL3L<40671>	ENSG00000130119	guanine nucleotide binding protein-like 3 (nucleolar)-like	ENSMUSG00000025266	NS	0.021270774	0.096882496	0.165953079	0.247925091
IGF2BP1<4765>	ENSG00000159217	insulin-like growth factor 2 mRNA binding protein 1	ENSMUSG00000013415	NS	0.043048348	-0.096339474	0.168350304	0.250898877
RBM42<39232>	ENSG00000126254	RNA binding motif protein 42	ENSMUSG00000036733	NS	0.026450913	0.095405146	0.172535226	0.256516189
GOT2<26693>	ENSG00000125166	glutamic-oxaloacetic transaminase 2, mitochondrial	ENSMUSG00000031672	NS	0.024164372	0.094437865	0.176948597	0.2624454
MRPS30<31993>	ENSG00000112996	mitochondrial ribosomal protein S30	ENSMUSG00000021731	NS	0.038691111	-0.093722531	0.180265735	0.266724069
RC3H2<32056>	ENSG00000056586	ring finger and CCCH-type domains 2	ENSMUSG00000075376	NS	0.000721128	-0.092552733	0.185788753	0.274238492

RBFOX1<26019>	ENSG00000078328	RNA binding protein, fox-1 homolog (C. elegans) 1	ENSMUSG00000008658	0.028593195	NS	-0.091994607	0.188467192	0.277528073
PPARGC1A<25859>	ENSG00000109819	peroxisome proliferator-activated receptor gamma, coactivator 1 alpha	ENSMUSG00000029167	NS	0.031520776	0.091794835	0.189432734	0.278285657
PEBP1<28049>	ENSG00000089220	phosphatidylethanolamine binding protein 1	ENSMUSG00000032959	NS	0.018953027	-0.091677443	0.190001799	0.278458695
FLNB<31085>	ENSG00000136068	filamin B, beta	ENSMUSG00000025278	NS	0.029663787	0.091507191	0.19082933	0.279008716
NCBP1<27498>	ENSG00000136937	nuclear cap binding protein subunit 1, 80kDa	ENSMUSG00000028330	NS	0.033189508	-0.090121832	0.197661109	0.287913737
NOC3L<36657>	ENSG00000173145	nucleolar complex associated 3 homolog (S. cerevisiae)	ENSMUSG00000024999	NS	0.01619688	0.090083387	0.197853193	0.287913737
PAN3<21811>	ENSG00000152520	PAN3 poly(A) specific ribonuclease subunit homolog (S. cerevisiae)	ENSMUSG00000029647	NS	0.001488038	-0.089442195	0.201076893	0.291916347
RSL1D1<16309>	ENSG00000171490	ribosomal L1 domain containing 1	ENSMUSG00000005846	0.047037695	0.004575223	-0.088489331	0.205937574	0.298271125
SEC61B<24607>	ENSG00000106803	Sec61 beta subunit	ENSMUSG00000053317	0.012409608	0.005609256	0.088352031	0.206644885	0.298594621
POLDIP3<31388>	ENSG00000100227	polymerase (DNA-directed), delta interacting protein 3	ENSMUSG00000041815	NS	0.002155067	0.088017705	0.208374505	0.300390342
EEF1G<40658>	ENSG00000254772	eukaryotic translation elongation factor 1 gamma	ENSMUSG00000071644	NS	0.026634508	-0.087305117	0.212095756	0.305042211
EEF1G<40658>	ENSG00000254772	microRNA 3654	ENSMUSG00000071644	NS	0.026634508	-0.087305117	0.212095756	0.305042211
PRRC2C<44763>	ENSG00000117523	proline-rich coiled-coil 2C	ENSMUSG00000040225	NS	0.047572195	-0.087184979	0.2127278	0.305239657
SMG1<27289>	ENSG00000157106	SMG1 phosphatidylinositol 3-kinase-related kinase	ENSMUSG00000030655	NS	0.013375389	-0.086856145	0.214464698	0.307017912
SMG1<27289>	ENSG00000157106	bolA family member 2	ENSMUSG00000030655	NS	0.013375389	-0.086856145	0.214464698	0.307017912
SMG1<27289>	ENSG00000157106	serine/threonine-protein kinase SMG1-like	ENSMUSG00000030655	NS	0.013375389	-0.086856145	0.214464698	0.307017912
DIDO1<39468>	ENSG00000101191	death inducer-obliterator 1	ENSMUSG00000038914	NS	0.015665573	-0.086736694	0.215098145	0.30721187
SRSF10<29069>	ENSG00000188529	serine/arginine-rich splicing factor 10-like	ENSMUSG00000028676	NS	0.001637497	-0.08503417	0.224272372	0.319575221
SRSF10<29069>	ENSG00000188529	serine/arginine-rich splicing factor 10	ENSMUSG00000028676	NS	0.001637497	-0.08503417	0.224272372	0.319575221
HEXIM1<36604>	ENSG00000186834	hexamethylene bis-acetamide inducible 1	ENSMUSG00000048878	NS	0.036180164	0.084765062	0.225747528	0.320299877
MRPL39<37069>	ENSG00000154719	mitochondrial ribosomal protein L39	ENSMUSG00000022889	NS	0.003354059	-0.084752018	0.225819203	0.320299877
FAM32A<10265>	ENSG00000105058	family with sequence similarity 32, member A	ENSMUSG00000003039	NS	0.029473277	-0.084033938	0.229789964	0.325184472
UCHL5<20779>	ENSG00000116750	ubiquitin carboxyl-terminal hydrolase L5	ENSMUSG00000018189	NS	0.027702817	0.083765516	0.231286838	0.325970397
METAP2<10260>	ENSG00000111142	methionyl aminopeptidase 2	ENSMUSG00000036112	NS	0.032579342	-0.083744921	0.231401971	0.325970397
SUPT16H<34287>	ENSG00000092201	suppressor of Ty 16 homolog (S. cerevisiae)	ENSMUSG00000035726	NS	0.048560115	0.083111967	0.234960113	0.33022866
POLR2A<10579>	ENSG00000181222	polymerase (RNA) II (DNA directed) polypeptide A, 220kDa	ENSMUSG00000005198	NS	0.000157833	-0.082419287	0.238897871	0.33500001
SRSF6<19477>	ENSG00000124193	serine/arginine-rich splicing factor 6	ENSMUSG00000016921	NS	0.007051708	0.082210591	0.240093281	0.335218892
NAA15<24708>	ENSG00000164134	N(alpha)-acetyltransferase 15, NatA auxiliary subunit	ENSMUSG00000063273	NS	0.001365538	0.082202353	0.240140554	0.335218892
DDX5<31689>	ENSG00000263077	microRNA 3064	NA	NS	0.018803141	-0.081409444	0.244721079	0.340841803
DDX5<31689>	ENSG00000108654	microRNA 3064	ENSMUSG00000020719	NS	0.018803141	-0.081409444	0.244721079	0.340841803
DDX5<31689>	ENSG00000108654	DEAD (Asp-Glu-Ala-Asp) box helicase 5	ENSMUSG00000020719	NS	0.018803141	-0.081409444	0.244721079	0.340841803
DDX5<31689>	ENSG00000263077	DEAD (Asp-Glu-Ala-Asp) box helicase 5	NA	NS	0.018803141	-0.081409444	0.244721079	0.340841803
DDX5<31689>	ENSG00000108654	microRNA 5047	ENSMUSG00000020719	NS	0.018803141	-0.081409444	0.244721079	0.340841803
DDX5<31689>	ENSG00000263077	microRNA 5047	NA	NS	0.018803141	-0.081409444	0.244721079	0.340841803
DZIP3<43033>	ENSG00000198919	DAZ interacting zinc finger protein 3	ENSMUSG00000064061	NS	0.012836944	-0.08129823	0.245368378	0.340973655
POP1<43294>	ENSG00000104356	processing of precursor 1, ribonuclease P/MRP subunit (S. cerevisiae)	ENSMUSG00000022325	0.048113434	NS	0.079671909	0.25497046	0.353000331
NELFE<42137>	ENSG00000206357	microRNA 1236	NA	0.040859904	NS	-0.079564815	0.25561174	0.353000331
NELFE<42137>	ENSG00000206268	negative elongation factor complex member E	NA	0.040859904	NS	-0.079564815	0.25561174	0.353000331
NELFE<42137>	ENSG00000229363	microRNA 1236	NA	0.040859904	NS	-0.079564815	0.25561174	0.353000331
NELFE<42137>	ENSG00000233801	negative elongation factor complex member E	NA	0.040859904	NS	-0.079564815	0.25561174	0.353000331
NELFE<42137>	ENSG00000204356	negative elongation factor complex member E	ENSMUSG00000024369	0.040859904	NS	-0.079564815	0.25561174	0.353000331
NELFE<42137>	ENSG00000229363	negative elongation factor complex member E	NA	0.040859904	NS	-0.079564815	0.25561174	0.353000331
NELFE<42137>	ENSG00000206268	microRNA 1236	NA	0.040859904	NS	-0.079564815	0.25561174	0.353000331
NELFE<42137>	ENSG00000233801	microRNA 1236	NA	0.040859904	NS	-0.079564815	0.25561174	0.353000331
NELFE<42137>	ENSG00000206357	negative elongation factor complex member E	NA	0.040859904	NS	-0.079564815	0.25561174	0.353000331
NELFE<42137>	ENSG00000231044	negative elongation factor complex member E	NA	0.040859904	NS	-0.079564815	0.25561174	0.353000331
NELFE<42137>	ENSG00000231044	microRNA 1236	NA	0.040859904	NS	-0.079564815	0.25561174	0.353000331
NELFE<42137>	ENSG00000204356	microRNA 1236	ENSMUSG00000024369	0.040859904	NS	-0.079564815	0.25561174	0.353000331
ZC3H14<21750>	ENSG00000100722	zinc finger CCCH-type containing 14	ENSMUSG00000021012	NS	0.011319192	-0.079543533	0.255739306	0.353000331
DDX24<19453>	ENSG00000089737	DEAD (Asp-Glu-Ala-Asp) box helicase 24	ENSMUSG00000041645	NS	7.83E-05	-0.079072593	0.258573467	0.356115735
RPS9<33775>	ENSG00000271572	ribosomal protein S9	NA	NS	0.037800203	-0.078114237	0.264407478	0.363339482
RPS9<33775>	ENSG00000263332	ribosomal protein S9	NA	NS	0.037800203	-0.078114237	0.264407478	0.363339482
RPS9<33775>	ENSG00000263076	ribosomal protein S9	NA	NS	0.037800203	-0.078114237	0.264407478	0.363339482
RPS9<33775>	ENSG00000170889	ribosomal protein S9	ENSMUSG00000006333	NS	0.037800203	-0.078114237	0.264407478	0.363339482
RRP12<2016>	ENSG00000052749	ribosomal RNA processing 12 homolog (S. cerevisiae)	ENSMUSG00000035049	NS	0.002479081	0.077779911	0.266463726	0.36369962
TUBB4A<17674>	ENSG00000104833	tubulin, beta 4A class IVa	ENSMUSG00000062591	NS	0.021633832	-0.077744213	0.266683928	0.36369962
HSP90AA1<5909>	ENSG00000080824	heat shock protein 90kDa alpha (cytosolic), class A member 1	ENSMUSG00000021270	NS	0.017538523	0.077670071	0.267141667	0.36369962
RPS3A<15848>	ENSG00000145425	small nucleolar RNA, C/D box 73A	ENSMUSG00000059751	NS	0.03090072	0.077665265	0.267171354	0.36369962
RPS3A<15848>	ENSG00000145425	ribosomal protein S3A	ENSMUSG00000059751	NS	0.03090072	0.077665265	0.267171354	0.36369962
PRPF38B<26849>	ENSG00000134186	PRP38 pre-mRNA processing factor 38 (yeast) domain containing B	ENSMUSG00000027881	NS	0.005924509	0.077593183	0.267616926	0.36369962
PKM<33586>	ENSG00000067225	pyruvate kinase, muscle	ENSMUSG00000032294	NS	0.001158026	-0.076703477	0.273158317	0.370414662
MTHFSD<11903>	ENSG00000103248	methenyltetrahydrofolate synthetase domain containing	ENSMUSG00000031816	NS	0.030428993	-0.075465029	0.281000643	0.380213531
LEMD3<6217>	ENSG00000174106	LEM domain containing 3	ENSMUSG00000048661	NS	0.024462915	-0.07377349	0.291955053	0.394171328
MRPL20<19030>	ENSG00000242485	mitochondrial ribosomal protein L20	ENSMUSG00000029066	NS	0.001346963	0.072769825	0.298587666	0.402245875
H1FX<34414>	ENSG00000184897	H1 histone family, member X	ENSMUSG00000044927	NS	0.041568622	-0.071867075	0.304638046	0.407660447
CSTF2T<2266>	ENSG00000177613	cleavage stimulation factor, 3' pre-RNA, subunit 2, 64kDa, tau variant	ENSMUSG00000053536	NS	0.004602377	-0.071840988	0.304814079	0.407660447
MOV10L1<31093>	ENSG00000073146	Mov10l1, Moloney leukemia virus 10-like 1, homolog (mouse)	ENSMUSG00000015365	NS	0.005432174	0.071776457	0.305249815	0.407660447
TAGLN2<22564>	ENSG00000158710	transgelin 2	ENSMUSG00000026547	NS	0.004878928	-0.071776457	0.305249815	0.407660447
HNRNPH3<6603>	ENSG00000096746	heterogeneous nuclear ribonucleoprotein H3 (2H9)	ENSMUSG00000020069	NS	0.005447479	-0.071545106	0.306815347	0.408866177
ZCCHC6<5439>	ENSG00000083223	zinc finger, CCHC domain containing 6	ENSMUSG00000035248	NS	0.018178184	-0.06971558	0.319381236	0.424694397

RPL27A<2541>	ENSG00000166441	ribosomal protein L27a	ENSMUSG00000046364	0.030395731	NS	0.068338459	0.329057252	0.436515451
RPL27A<2541>	ENSG00000166441	small nucleolar RNA, H/ACA box 3	ENSMUSG00000046364	0.030395731	NS	0.068338459	0.329057252	0.436515451
NHP2<10523>	ENSG00000145912	NHP2 ribonucleoprotein	ENSMUSG00000001056	NS	0.006109002	0.0682499	0.329685876	0.436515451
NHP2<10523>	LRG_346	NHP2 ribonucleoprotein	NA	NS	0.006109002	0.0682499	0.329685876	0.436515451
NCOA5<8843>	ENSG00000124160	nuclear receptor coactivator 5	ENSMUSG00000039804	NS	0.046553574	-0.067386281	0.335856614	0.443733452
SBDS<40535>	ENSG00000126524	Shwachman-Bodian-Diamond syndrome	ENSMUSG00000025337	NS	0.00245771	0.067042344	0.338334532	0.446052108
TWISTNB<33945>	ENSG00000105849	TWIST neighbor	ENSMUSG00000020561	NS	0.000429568	-0.066342799	0.343410298	0.449600972
NDUFV3<26212>	ENSG00000160194	NADH dehydrogenase (ubiquinone) flavoprotein 3, 10kDa	ENSMUSG00000024038	NS	0.023325976	-0.066302982	0.343700648	0.449600972
HSPA1A<1927>	ENSG00000237724	heat shock 70kDa protein 1A	NA	NS	0.028485534	0.06627003	0.343941056	0.449600972
HSPA1A<1927>	ENSG00000204389	heat shock 70kDa protein 1A	ENSMUSG00000090877	NS	0.028485534	0.06627003	0.343941056	0.449600972
HSPA1A<1927>	ENSG00000237724	heat shock 70kDa protein 1B	NA	NS	0.028485534	0.06627003	0.343941056	0.449600972
HSPA1A<1927>	ENSG00000234475	heat shock 70kDa protein 1B	NA	NS	0.028485534	0.06627003	0.343941056	0.449600972
HSPA1A<1927>	ENSG00000215328	heat shock 70kDa protein 1B	NA	NS	0.028485534	0.06627003	0.343941056	0.449600972
HSPA1A<1927>	ENSG00000234475	heat shock 70kDa protein 1A	NA	NS	0.028485534	0.06627003	0.343941056	0.449600972
HSPA1A<1927>	ENSG00000235941	heat shock 70kDa protein 1A	NA	NS	0.028485534	0.06627003	0.343941056	0.449600972
HSPA1A<1927>	ENSG00000204389	heat shock 70kDa protein 1B	ENSMUSG00000090877	NS	0.028485534	0.06627003	0.343941056	0.449600972
HSPA1A<1927>	ENSG00000215328	heat shock 70kDa protein 1A	NA	NS	0.028485534	0.06627003	0.343941056	0.449600972
HSPA1A<1927>	ENSG00000235941	heat shock 70kDa protein 1B	NA	NS	0.028485534	0.06627003	0.343941056	0.449600972
HSPA1B<1927>	ENSG00000231555	heat shock 70kDa protein 1B	NA	NS	0.028485534	0.06627003	0.343941056	0.449600972
HSPA1B<1927>	ENSG00000212866	heat shock 70kDa protein 1A	NA	NS	0.028485534	0.06627003	0.343941056	0.449600972
HSPA1B<1927>	ENSG00000231555	heat shock 70kDa protein 1A	NA	NS	0.028485534	0.06627003	0.343941056	0.449600972
HSPA1B<1927>	ENSG00000232804	heat shock 70kDa protein 1A	NA	NS	0.028485534	0.06627003	0.343941056	0.449600972
HSPA1B<1927>	ENSG00000224501	heat shock 70kDa protein 1B	NA	NS	0.028485534	0.06627003	0.343941056	0.449600972
HSPA1B<1927>	ENSG00000212866	heat shock 70kDa protein 1B	NA	NS	0.028485534	0.06627003	0.343941056	0.449600972
HSPA1B<1927>	ENSG00000204388	heat shock 70kDa protein 1A	ENSMUSG00000090877	NS	0.028485534	0.06627003	0.343941056	0.449600972
HSPA1B<1927>	ENSG00000204388	heat shock 70kDa protein 1B	ENSMUSG00000090877	NS	0.028485534	0.06627003	0.343941056	0.449600972
HSPA1B<1927>	ENSG00000224501	heat shock 70kDa protein 1A	NA	NS	0.028485534	0.06627003	0.343941056	0.449600972
HSPA1B<1927>	ENSG00000232804	heat shock 70kDa protein 1B	NA	NS	0.028485534	0.06627003	0.343941056	0.449600972
HNRNPUL1<44130>	ENSG00000105323	heterogeneous nuclear ribonucleoprotein U-like 1	ENSMUSG00000040725	NS	0.022079228	-0.066090167	0.345255158	0.450364605
DIEXF<19401>	ENSG00000117597	digestive organ expansion factor homolog (zebrafish)	ENSMUSG00000016181	NS	0.047739104	-0.065709159	0.348049325	0.453051515
IGF2BP1<41254>	ENSG00000159217	insulin-like growth factor 2 mRNA binding protein 1	ENSMUSG00000013415	NS	0.019133269	-0.065085129	0.352656448	0.458082103
CSDC2<39875>	ENSG00000172346	cold shock domain containing C2, RNA binding	ENSMUSG00000042109	NS	1.60E-05	-0.064979408	0.35344075	0.458136499
ZFC3H1<17209>	ENSG00000133858	zinc finger, C3H1-type containing	ENSMUSG00000034163	NS	0.046053308	0.064298399	0.358519078	0.463744832
WDR33<30031>	ENSG00000136709	SFT2 domain containing 3	ENSMUSG00000024400	NS	0.0069658	-0.063881006	0.361654001	0.466821167
WDR33<30031>	ENSG00000136709	WD repeat domain 33	ENSMUSG00000024400	NS	0.0069658	-0.063881006	0.361654001	0.466821167
GNL3L<33842>	ENSG00000130119	guanine nucleotide binding protein-like 3 (nucleolar)-like	ENSMUSG00000025266	NS	0.026450765	-0.062209375	0.374379322	0.482238054
RPL15<30794>	ENSG00000174748	ribosomal protein L15	ENSMUSG00000012405	NS	0.033280321	0.062103654	0.375193261	0.482279721
UTP3<20717>	ENSG00000132467	UTP3, small subunit (SSU) processome component, homolog (S. cerevisiae)	ENSMUSG00000070697	NS	0.025815156	0.061855827	0.377105513	0.483729924
UTP23<36066>	ENSG00000147679	UTP23, small subunit (SSU) processome component, homolog (yeast)	ENSMUSG00000022313	NS	0.020012439	0.061120584	0.38281371	0.490033305
TAF15<44831>	ENSG00000172660	TAF15 RNA polymerase II, TATA box binding protein (TBP)-associated factor, 68k	ENSMUSG00000020680	NS	0.041257524	0.060346897	0.388876789	0.496763945
TAF15<44831>	ENSG00000270647	TAF15 RNA polymerase II, TATA box binding protein (TBP)-associated factor, 68k	NA	NS	0.041257524	0.060346897	0.388876789	0.496763945
RPL27A<14935>	ENSG00000166441	ribosomal protein L27a	ENSMUSG00000046364	NS	0.02026055	0.058509133	0.403509398	0.514391115
RPL27A<14935>	ENSG00000166441	small nucleolar RNA, H/ACA box 3	ENSMUSG00000046364	NS	0.02026055	0.058509133	0.403509398	0.514391115
CS<10773>	ENSG00000062485	citrate synthase	ENSMUSG00000046934	NS	0.030868727	-0.057320113	0.413148568	0.524639797
HNRNPA0<13374>	ENSG00000177733	heterogeneous nuclear ribonucleoprotein A0	ENSMUSG00000007836	NS	0.021532788	-0.057307756	0.41324945	0.524639797
EIF4ENIF1<5431>	ENSG00000184708	eukaryotic translation initiation factor 4E nuclear import factor 1	ENSMUSG00000020454	NS	0.011686225	0.056063815	0.423478778	0.536522422
RPL6<38697>	ENSG00000089009	ribosomal protein L6	ENSMUSG00000029614	NS	0.044434863	-0.055814615	0.425545573	0.538036138
THRAP3<40173>	ENSG00000054118	thyroid hormone receptor associated protein 3	ENSMUSG00000043962	NS	0.010704693	-0.055016901	0.432200769	0.545333116
LRP1<25462>	ENSG00000123384	low density lipoprotein receptor-related protein 1	ENSMUSG00000040249	NS	0.00244284	-0.0543558	0.437761279	0.551221882
IFIT5<14532>	ENSG00000152778	interferon-induced protein with tetratricopeptide repeats 5	NA	NS	0.017958396	0.054002252	0.440751655	0.553857024
USP36<5574>	ENSG00000055483	ubiquitin specific peptidase 36	ENSMUSG00000033909	NS	0.025890445	0.053048015	0.448880528	0.562924518
RRP15<3689>	ENSG00000067533	ribosomal RNA processing 15 homolog (S. cerevisiae)	ENSMUSG00000001305	0.038382808	0.017254352	0.052941607	0.449792189	0.562924518
RPL23A<26198>	ENSG00000198242	ribosomal protein L23a	ENSMUSG00000063556	NS	0.038984056	0.052472041	0.453827682	0.566825285
RPL23A<26198>	ENSG00000198242	small nucleolar RNA, C/D box 42A	ENSMUSG00000063556	NS	0.038984056	0.052472041	0.453827682	0.566825285
RPS9<19930>	ENSG00000271572	ribosomal protein S9	NA	NS	0.002018157	0.051904304	0.458733808	0.571795464
RPS9<19930>	ENSG00000263332	ribosomal protein S9	NA	NS	0.002018157	0.051904304	0.458733808	0.571795464
RPS9<19930>	ENSG00000263076	ribosomal protein S9	NA	NS	0.002018157	0.051904304	0.458733808	0.571795464
RPS9<19930>	ENSG00000170889	ribosomal protein S9	ENSMUSG00000006333	NS	0.002018157	0.051904304	0.458733808	0.571795464
DDX17<43710>	ENSG00000100201	DEAD (Asp-Glu-Ala-Asp) box helicase 17	ENSMUSG00000055065	NS	0.00162487	-0.05160087	0.461367999	0.573919468
CCDC59<11908>	ENSG00000133773	coiled-coil domain containing 59	ENSMUSG00000019897	NS	0.020971714	0.05141071	0.463023104	0.574819422
RBM11<25227>	ENSG00000185272	RNA binding motif protein 11	ENSMUSG00000032940	0.036906477	NS	-0.049158299	0.482875369	0.598261289
EIF4B<32368>	ENSG00000063046	eukaryotic translation initiation factor 4B	ENSMUSG00000058655	NS	0.036540429	0.048939991	0.484823579	0.599471265
TRIM28<6987>	ENSG00000130726	tripartite motif containing 28	ENSMUSG00000005566	NS	0.011698484	-0.04718804	0.500610226	0.617752987
SF3B1<26422>	ENSG00000115524	splicing factor 3b, subunit 1, 155kDa	ENSMUSG00000025982	NS	0.001636494	0.04670337	0.505024818	0.62195669
NOSIP<4384>	ENSG00000142546	nitric oxide synthase interacting protein	ENSMUSG00000003421	NS	0.019723545	-0.046011377	0.511362916	0.628507787
AKAP17A<6211>	ENSG00000197976	A kinase (PRKA) anchor protein 17A	ENSMUSG00000059708	0.041173274	NS	-0.045517782	0.515908903	0.632834575
EIF2S1<2144>	ENSG00000134001	eukaryotic translation initiation factor 2, subunit 1 alpha, 35kDa	ENSMUSG00000021116	NS	0.041480876	0.04526309	0.518262721	0.634135739
CDKN2A<1687>	LRG_11	cyclin-dependent kinase inhibitor 2A	NA	NS	0.040324541	0.04518071	0.519025241	0.634135739
CDKN2A<1687>	ENSG00000147889	cyclin-dependent kinase inhibitor 2A	NA	NS	0.040324541	0.04518071	0.519025241	0.634135739
LSM14A<41585>	ENSG00000257103	LSM14A, SCD6 homolog A (S. cerevisiae)	ENSMUSG00000066568	NS	0.034070264	0.044636315	0.524078647	0.639044459

LSM14A<41585>	ENSG00000262860	LSM14A, SCD6 homolog A (S. cerevisiae)	NA	NS	0.034070264	0.044636315	0.524078647	0.639044459
CPNE3<30795>	ENSG00000085719	copine III	ENSMUSG00000028228	NS	0.0286814	-0.044335627	0.526880503	0.640414057
ARF1<24823>	ENSG00000143761	ADP-ribosylation factor 1	ENSMUSG00000048076	NS	0.019287453	-0.044293064	0.527277725	0.640414057
ARF1<24823>	ENSG00000143761	microRNA 3620	ENSMUSG00000048076	NS	0.019287453	-0.044293064	0.527277725	0.640414057
PAK1IP1<18110>	ENSG00000111845	PAK1 interacting protein 1	ENSMUSG00000038683	NS	8.85E-05	0.044113887	0.528951563	0.641184945
RPL7L1<37100>	ENSG00000146223	ribosomal protein L7-like 1	ENSMUSG00000063888	NS	0.018226055	-0.043956678	0.530422387	0.6417071
NKRF<16516>	ENSG00000186416	NFKB repressing factor	ENSMUSG00000044149	NS	0.039928241	-0.042250036	0.546521002	0.659733034
UCHL5<6664>	ENSG00000116750	ubiquitin carboxyl-terminal hydrolase L5	ENSMUSG00000018189	NS	0.01659885	-0.04215118	0.547460813	0.659733034
RBM4<23108>	ENSG00000173933	RNA binding motif protein 4	ENSMUSG00000096370	NS	0.000123126	-0.041674062	0.552007832	0.662624149
RBM4<23108>	ENSG00000173933	RBM14-RBM4 readthrough	ENSMUSG00000096370	NS	0.000123126	-0.041674062	0.552007832	0.662624149
RBM14<23108>	ENSG00000239306	RNA binding motif protein 14	ENSMUSG00000006456	NS	0.000123126	-0.041674062	0.552007832	0.662624149
YWHAE<23696>	ENSG00000108953	tyrosine 3-monooxygenase/tryptophan 5-monooxygenase activation protein, ep	ENSMUSG00000020849	NS	0.011423076	0.041247744	0.556086219	0.666223661
RPL21<39079>	ENSG00000122026	small nucleolar RNA, C/D box 102	ENSMUSG00000059912	NS	0.013153337	-0.041028064	0.558193486	0.667452305
RPL21<39079>	ENSG00000122026	small nucleolar RNA, H/ACA box 27	ENSMUSG00000059912	NS	0.013153337	-0.041028064	0.558193486	0.667452305
RPL21<39079>	ENSG00000122026	ribosomal protein L21	ENSMUSG00000059912	NS	0.013153337	-0.041028064	0.558193486	0.667452305
CSTB<2695>	ENSG00000160213	cystatin B (stefin B)	ENSMUSG00000005054	NS	0.042867724	0.040521426	0.563068012	0.671978638
SSR1<22477>	ENSG00000124783	signal sequence receptor, alpha	ENSMUSG00000021427	NS	0.013770811	-0.040037442	0.567743522	0.675634972
PRPF8<9759>	ENSG00000174231	pre-mRNA processing factor 8	ENSMUSG00000020850	NS	0.008605517	0.039977717	0.568321776	0.675634972
ZFP36<2800>	ENSG00000128016	ZFP36 ring finger protein	ENSMUSG00000044786	NS	0.045662848	0.039657807	0.571423841	0.678016317
NOL6<3901>	ENSG00000165271	nucleolar protein 6 (RNA-associated)	ENSMUSG00000028430	NS	0.006513229	-0.03883126	0.579475418	0.686250138
CAST<19869>	ENSG00000153113	calpastatin	ENSMUSG00000021585	NS	0.036541318	0.036606309	0.601407542	0.710859057
RBM22<17636>	ENSG00000086589	RNA binding motif protein 22	ENSMUSG00000024604	NS	0.02025002	-0.035998755	0.607460415	0.71549098
YWHAE<43014>	ENSG00000108953	tyrosine 3-monooxygenase/tryptophan 5-monooxygenase activation protein, ep	ENSMUSG00000020849	NS	0.020780734	0.03598022	0.607645502	0.71549098
RBMX<43325>	ENSG00000147274	uncharacterized LOC101928747	ENSMUSG00000091987	NS	0.001611536	0.035766718	0.609779233	0.715716364
RBMX<43325>	ENSG00000147274	small nucleolar RNA, C/D box 61	ENSMUSG00000091987	NS	0.001611536	0.035766718	0.609779233	0.715716364
RBMX<43325>	ENSG00000147274	RNA binding motif protein, X-linked	ENSMUSG00000091987	NS	0.001611536	0.035766718	0.609779233	0.715716364
RPS26<11047>	ENSG00000197728	40S ribosomal protein S26-like	ENSMUSG00000059775	NS	0.007729863	-0.03572896	0.610156925	0.715716364
RPS26<11047>	ENSG00000197728	ribosomal protein S26	ENSMUSG00000059775	NS	0.007729863	-0.03572896	0.610156925	0.715716364
EIF4G3<13409>	ENSG00000075151	eukaryotic translation initiation factor 4 gamma, 3	ENSMUSG00000028760	NS	0.03788329	-0.035480447	0.61264539	0.717271749
CHD2<26222>	ENSG00000173575	chromodomain helicase DNA binding protein 2	ENSMUSG00000078671	NS	0.025561128	-0.035276556	0.614690349	0.71830287
RNF113A<38319>	ENSG00000125352	ring finger protein 113A	ENSMUSG00000036537	NS	0.003047756	-0.034912024	0.6183539	0.721218065
RIMS1<8407>	ENSG00000079841	regulating synaptic membrane exocytosis 1	ENSMUSG00000041670	NS	0.021253383	0.03437518	0.623766354	0.725910958
RPS3A<8966>	ENSG00000145425	small nucleolar RNA, C/D box 73A	ENSMUSG00000059751	NS	0.039427884	0.034279756	0.62473054	0.725910958
RPS3A<8966>	ENSG00000145425	ribosomal protein S3A	ENSMUSG00000059751	NS	0.039427884	0.034279756	0.62473054	0.725910958
PARP1<31452>	ENSG00000143799	poly (ADP-ribose) polymerase 1	ENSMUSG00000026496	NS	0.001338813	-0.033885018	0.628725842	0.728891735
FAM120C<34009>	ENSG00000184083	family with sequence similarity 120C	ENSMUSG00000025262	NS	0.013510317	-0.033793027	0.629658479	0.728891735
FAM120C<34009>	ENSG00000268158	family with sequence similarity 120C	NA	NS	0.013510317	-0.033793027	0.629658479	0.728891735
YWHAZ<27973>	ENSG00000164924	tyrosine 3-monooxygenase/tryptophan 5-monooxygenase activation protein, ze	ENSMUSG00000022285	NS	0.045411848	0.033033756	0.637378501	0.736063124
KRT18<1543>	ENSG00000111057	keratin 18	ENSMUSG00000023043	NS	0.024053972	0.032949317	0.638239495	0.736063124
SRRM1<21373>	ENSG00000133226	serine/arginine repetitive matrix 1	ENSMUSG00000028809	NS	0.006571943	-0.032182495	0.646080503	0.743715799
GAPDH<10338>	ENSG00000111640	glyceraldehyde-3-phosphate dehydrogenase	ENSMUSG00000081607	NS	0.037767747	0.03126121	0.655552752	0.752934963
SSR1<16684>	ENSG00000124783	signal sequence receptor, alpha	ENSMUSG00000021427	NS	0.011126903	-0.031166473	0.656529957	0.752934963
WDR46<31417>	ENSG00000236222	WD repeat domain 46	NA	NS	0.015288228	-0.03085892	0.65970635	0.755174002
WDR46<31417>	ENSG00000226916	WD repeat domain 46	NA	NS	0.015288228	-0.03085892	0.65970635	0.755174002
WDR46<31417>	ENSG00000204221	WD repeat domain 46	NA	NS	0.015288228	-0.03085892	0.65970635	0.755174002
WDR46<31417>	ENSG00000227057	WD repeat domain 46	ENSMUSG00000024312	NS	0.015288228	-0.03085892	0.65970635	0.755174002
WDR46<31417>	ENSG00000206284	WD repeat domain 46	NA	NS	0.015288228	-0.03085892	0.65970635	0.755174002
SBDS<37978>	ENSG00000126524	Shwachman-Bodian-Diamond syndrome	ENSMUSG00000025337	NS	0.01480565	0.030285005	0.665650002	0.760566759
SUMO1<16945>	ENSG00000116030	small ubiquitin-like modifier 1	ENSMUSG00000026021	NS	0.043072038	-0.030047476	0.668116075	0.761973445
DDX51<31478>	ENSG00000185163	DEAD (Asp-Glu-Ala-Asp) box polypeptide 51	ENSMUSG00000029504	NS	0.025165908	0.029536032	0.673438031	0.766625915
GDI2<24852>	ENSG00000057608	GDP dissociation inhibitor 2	ENSMUSG00000021218	NS	0.000630945	-0.028780194	0.681332833	0.774184784
PUM1<42886>	ENSG00000134644	pumilio RNA-binding family member 1	ENSMUSG00000028580	NS	0.000597502	-0.028462344	0.684663246	0.77653896
TNS1<22299>	ENSG00000079308	tensin 1	ENSMUSG00000055322	NS	0.028817901	-0.028336714	0.685981271	0.776606352
TNS1<6713>	ENSG00000079308	tensin 1	ENSMUSG00000055322	NS	0.000925026	0.026828471	0.701877828	0.791540213
ADAR<28758>	ENSG00000160710	adenosine deaminase, RNA-specific	ENSMUSG00000027951	NS	0.029237811	-0.026697349	0.703266081	0.791540213
CDK11B<6950>	ENSG00000248333	cyclin-dependent kinase 11B	ENSMUSG00000029062	NS	0.012554736	-0.026684992	0.703396962	0.791540213
CDK11B<6950>	ENSG00000248333	cyclin-dependent kinase 11A	ENSMUSG00000029062	NS	0.012554736	-0.026684992	0.703396962	0.791540213
PARP12<38858>	ENSG00000059378	poly (ADP-ribose) polymerase family, member 12	ENSMUSG00000038507	NS	0.034686061	0.026515426	0.705193819	0.791540213
RPL8<11639>	ENSG00000161016	ribosomal protein L8	ENSMUSG00000003970	NS	0.001981172	-0.026478355	0.705586872	0.791540213
ZFP36L2<25306>	ENSG00000152518	ZFP36 ring finger protein-like 2	ENSMUSG00000045817	NS	0.004069563	-0.025434873	0.716682234	0.802527981
RPL12<21270>	ENSG00000197958	ribosomal protein L12	ENSMUSG00000069682	NS	0.004725099	-0.024917251	0.722208396	0.805905875
SAFB2<3768>	ENSG00000130254	scaffold attachment factor B2	ENSMUSG00000042625	NS	0.00975859	-0.02490764	0.722311141	0.805905875
ADARB1<41196>	ENSG00000197381	adenosine deaminase, RNA-specific, B1	ENSMUSG00000020262	NS	0.0100428	-0.023970566	0.732352391	0.815634352
NOP14<10035>	ENSG00000087269	NOP14 nucleolar protein	ENSMUSG00000036693	NS	0.000103982	0.023825714	0.733908672	0.815894897
UBFD1<11313>	ENSG00000103353	ubiquitin family domain containing 1	ENSMUSG00000030870	NS	0.002235676	0.02300054	0.742794849	0.822931781
LSM7<23784>	ENSG00000130332	LSM7 homolog, U6 small nuclear RNA associated (S. cerevisiae)	ENSMUSG00000064317	NS	0.029507916	-0.022990242	0.74290596	0.822931781
WDR33<2051>	ENSG00000136709	SFT2 domain containing 3	ENSMUSG00000024400	NS	0.007007511	-0.0221685	0.75178953	0.831279788
WDR33<2051>	ENSG00000136709	WD repeat domain 33	ENSMUSG00000024400	NS	0.007007511	-0.0221685	0.75178953	0.831279788
GTF2E2<39097>	ENSG00000197265	general transcription factor IIE, polypeptide 2, beta 34kDa	ENSMUSG00000031585	NS	0.038716794	-0.021439436	0.75969866	0.838522536
ZFP36L1<15973>	ENSG00000185650	ZFP36 ring finger protein-like 1	ENSMUSG00000021127	NS	0.025345148	-0.021261632	0.761631359	0.839154596

NGRN<25730>	ENSG00000182768	neugrin, neurite outgrowth associated	ENSMUSG00000047084	NS	0.040816593	-0.019942176	0.776019028	0.853482572
KHDRBS1<40216>	ENSG00000121774	KH domain containing, RNA binding, signal transduction associated 1	ENSMUSG00000028790	NS	0.028632484	0.019725242	0.778392002	0.854569153
ANXA2<16188>	ENSG00000182718	annexin A2	ENSMUSG00000032231	NS	0.044791244	-0.01942936	0.781631863	0.855823743
NSUN6<9088>	ENSG00000241058	NOP2/Sun domain family, member 6	ENSMUSG00000026707	NS	0.036972415	-0.019367575	0.782308877	0.855823743
ALG13<40663>	ENSG00000101901	ALG13, UDP-N-acetylglucosaminyltransferase subunit	NA	NS	0.005401814	0.019124553	0.784973383	0.856697632
ZC3H11A<627>	ENSG00000058673	zinc finger CCCH-type containing 11A	ENSMUSG00000026464	NS	0.004233876	-0.019041487	0.785884708	0.856697632
ZC3H11A<627>	ENSG00000058673	zinc finger CCCH-type domain-containing-like	ENSMUSG00000026464	NS	0.004233876	-0.019041487	0.785884708	0.856697632
TBL3<1003>	ENSG00000183751	transducin (beta)-like 3	ENSMUSG00000040688	NS	0.036380135	-0.018854758	0.787934368	0.856807585
MTDH<22813>	ENSG00000147649	metadherin	ENSMUSG00000022255	NS	0.010585089	-0.018768946	0.788876797	0.856807585
EIF3D<15955>	ENSG00000100353	eukaryotic translation initiation factor 3, subunit D	ENSMUSG00000016554	NS	2.36E-15	-0.018633018	0.790370232	0.856807585
RPL21<28289>	ENSG00000122026	small nucleolar RNA, C/D box 102	ENSMUSG00000059912	NS	0.034708106	0.018512194	0.791698372	0.856807585
RPL21<28289>	ENSG00000122026	small nucleolar RNA, H/ACA box 27	ENSMUSG00000059912	NS	0.034708106	0.018512194	0.791698372	0.856807585
RPL21<28289>	ENSG00000122026	ribosomal protein L21	ENSMUSG00000059912	NS	0.034708106	0.018512194	0.791698372	0.856807585
MAP4<25721>	ENSG00000047849	microtubule-associated protein 4	ENSMUSG00000032479	NS	0.029587332	-0.018400294	0.792928947	0.856807585
RPL11<29904>	ENSG00000142676	ribosomal protein L11	ENSMUSG00000059291	NS	0.005803906	-0.017812649	0.799399688	0.862289537
ANXA2<22723>	ENSG00000182718	annexin A2	ENSMUSG00000032231	NS	0.042844432	0.016830266	0.810247398	0.871916715
SRBD1<7441>	ENSG00000068784	S1 RNA binding domain 1	ENSMUSG00000024135	NS	0.00995069	0.016667565	0.812047536	0.871916715
PRRC2C<14256>	ENSG00000117523	proline-rich coiled-coil 2C	ENSMUSG00000040225	NS	0.043302811	0.016620883	0.812564214	0.871916715
DSP<1342>	ENSG00000096696	desmoplakin	ENSMUSG00000054889	NS	0.000960085	-0.015484723	0.825163705	0.883899311
LGALS3<8599>	ENSG00000131981	lectin, galactoside-binding, soluble, 3	ENSMUSG00000050335	NS	0.003351991	-0.015021335	0.830315529	0.887876367
CPEB4<19508>	ENSG00000113742	cytoplasmic polyadenylation element binding protein 4	ENSMUSG00000020300	NS	0.005827799	0.014465269	0.83650727	0.892949834
RBM45<25656>	ENSG00000155636	RNA binding motif protein 45	ENSMUSG00000042369	NS	0.032894516	-0.013942841	0.84233363	0.897616289
GNL2<30022>	ENSG00000134697	guanine nucleotide binding protein-like 2 (nucleolar)	ENSMUSG00000028869	NS	0.00105964	-0.01274421	0.855733261	0.910323183
LSG1<17127>	ENSG00000041802	large 60S subunit nuclear export GTPase 1	ENSMUSG00000022538	NS	0.018364822	0.012235513	0.861432783	0.913743076
P4HB<22323>	ENSG00000185624	prolyl 4-hydroxylase, beta polypeptide	ENSMUSG00000025130	NS	0.017698985	0.01219295	0.861909996	0.913743076
CNOT1<19481>	ENSG00000125107	CCR4-NOT transcription complex, subunit 1	ENSMUSG00000036550	NS	0.047631325	0.011952674	0.864604869	0.915027827
EIF3B<6314>	ENSG00000106263	eukaryotic translation initiation factor 3, subunit B	ENSMUSG00000056076	NS	0.003344181	-0.011772124	0.866630905	0.915601482
LRP1<12213>	ENSG00000123384	low density lipoprotein receptor-related protein 1	ENSMUSG00000040249	NS	0.004048495	-0.011521551	0.869444143	0.916437154
RPS27L<20512>	ENSG00000185088	ribosomal protein S27-like	ENSMUSG00000036781	NS	0.017763777	0.011437112	0.870392537	0.916437154
DDX52<9124>	ENSG00000141141	DEAD (Asp-Glu-Ala-Asp) box polypeptide 52	ENSMUSG00000020677	NS	0.002167003	0.010647635	0.879268411	0.921922132
SRSF3<1288>	ENSG00000112081	serine/arginine-rich splicing factor 3	ENSMUSG00000078134	NS	0.016308462	-0.010561823	0.880234108	0.921922132
DDX28<26854>	ENSG00000182810	DEAD (Asp-Glu-Ala-Asp) box polypeptide 28	ENSMUSG00000045538	NS	0.043828475	0.010532303	0.880566349	0.921922132
NOC4L<32489>	ENSG00000184967	nucleolar complex associated 4 homolog (S. cerevisiae)	ENSMUSG00000033294	NS	0.01881688	-0.010442371	0.881578653	0.921922132
PTBP1<12038>	ENSG00000011304	microRNA 4745	ENSMUSG00000006498	NS	0.006178463	0.009376235	0.893593667	0.932774868
PTBP1<12038>	ENSG00000011304	polypyrimidine tract binding protein 1	ENSMUSG00000006498	NS	0.006178463	0.009376235	0.893593667	0.932774868
PPIE<749>	ENSG00000084072	peptidylprolyl isomerase E (cyclophilin E)	ENSMUSG00000028651	NS	0.021994711	-0.009253351	0.89498014	0.932774868
BOLL<44634>	ENSG00000152430	bol, boule-like (Drosophila)	ENSMUSG00000025977	NS	0.021463117	0.009025433	0.897552538	0.933878402
EIF5<44319>	ENSG00000100664	eukaryotic translation initiation factor 5	ENSMUSG00000021282	NS	0.015172897	-0.008819482	0.899877914	0.934721657
CD3EAP<12111>	ENSG00000117877	CD3e molecule, epsilon associated protein	ENSMUSG00000047649	NS	0.016411741	-0.008480351	0.903708884	0.937123347
AKAP8L<43530>	ENSG00000011243	A kinase (PRKA) anchor protein 8-like	ENSMUSG00000002625	NS	0.021503145	0.008279552	0.905978245	0.937900251
BAG4<37382>	ENSG00000156735	BCL2-associated athanogene 4	ENSMUSG00000037316	NS	0.004525987	-0.008071196	0.90833382	0.938763743
TNRC6A<12122>	ENSG00000090905	trinucleotide repeat containing 6A	ENSMUSG00000052707	NS	0.018645327	-0.007818563	0.911191036	0.940141885
RPL13<28596>	ENSG00000167526	ribosomal protein L13	ENSMUSG00000059835	NS	4.03E-26	-0.007662041	0.912961842	0.940396378
RPL13<28596>	ENSG00000167526	small nucleolar RNA, C/D box 68	ENSMUSG00000059835	NS	4.03E-26	-0.007662041	0.912961842	0.940396378
DUT<12931>	ENSG00000128951	deoxyuridine triphosphatase	ENSMUSG00000027203	NS	0.026315996	-0.00700094	0.920445798	0.946525098
SSR1<20290>	ENSG00000124783	signal sequence receptor, alpha	ENSMUSG00000021427	NS	0.034384448	-0.006692015	0.923945413	0.946996873
HNRNPR<34010>	ENSG00000125944	heterogeneous nuclear ribonucleoprotein R	ENSMUSG00000066037	NS	0.043307907	-0.006639154	0.924544385	0.946996873
HMGN2<27122>	ENSG00000198830	high mobility group nucleosomal binding domain 2	ENSMUSG00000091775	NS	0.023324976	0.006554028	0.925509051	0.946996873
RPLP0<19931>	ENSG00000089157	ribosomal protein, large, P0	ENSMUSG00000067274	NS	0.012212579	0.006070731	0.930987887	0.951025719
ZC3H6<10590>	ENSG00000188177	zinc finger CCCH-type containing 6	ENSMUSG00000042851	NS	0.023048983	0.005588807	0.936454392	0.955028702
IMP3<283>	ENSG00000177971	IMP3, U3 small nucleolar ribonucleoprotein, homolog (yeast)	ENSMUSG00000032288	NS	0.022964629	0.005036174	0.942726609	0.959838799
NCL<27662>	ENSG00000115053	nucleolin	ENSMUSG00000026234	NS	0.015332958	0.004774617	0.945696452	0.961079792
HMGN5<22653>	ENSG00000198157	high mobility group nucleosome binding domain 5	ENSMUSG00000031245	NS	0.031747837	-0.004654479	0.947060807	0.961079792
NOL7<36168>	ENSG00000225921	nucleolar protein 7, 27kDa	ENSMUSG00000063200	NS	0.012435901	-0.003523811	0.959908341	0.972517933
ELAVL1<3793>	ENSG00000066044	ELAV (embryonic lethal, abnormal vision, Drosophila)-like 1 (Hu antigen R)	ENSMUSG00000040028	NS	0.004857921	-0.003258822	0.962920978	0.973970913
UBE2N<20429>	ENSG00000177889	ubiquitin-conjugating enzyme E2N	ENSMUSG00000074781	NS	0.002610138	0.003007562	0.965778007	0.975261908
RBMX<40610>	ENSG00000147274	uncharacterized LOC101928747	ENSMUSG00000091987	NS	0.01186432	0.002220145	0.974734247	0.982697715
RBMX<40610>	ENSG00000147274	small nucleolar RNA, C/D box 61	ENSMUSG00000091987	NS	0.01186432	0.002220145	0.974734247	0.982697715
RBMX<40610>	ENSG00000147274	RNA binding motif protein, X-linked	ENSMUSG00000091987	NS	0.01186432	0.002220145	0.974734247	0.982697715
WDR6<41692>	ENSG00000178252	WD repeat domain 6	ENSMUSG00000066357	NS	9.53E-65	-0.001606413	0.981717212	0.988123185
TACO1<38531>	ENSG00000136463	translational activator of mitochondrially encoded cytochrome c oxidase I	ENSMUSG00000001983	NS	0.00306215	0.000867051	0.990131375	0.994969176
IMMT<33959>	ENSG00000132305	inner membrane protein, mitochondrial	ENSMUSG00000052337	NS	0.040576687	-0.000363846	0.995858685	0.9983044
CNN3<35894>	ENSG00000117519	calponin 3, acidic	ENSMUSG00000053931	NS	0.017708301	-0.000248513	0.997171397	0.9983044
EEF1A1<9950>	ENSG00000156508	eukaryotic translation elongation factor 1 alpha 1	ENSMUSG00000037742	NS	0.046810845	0.000148971	0.998304399	0.9983044

Supplementary Table 2

RBP<Probe_ID>	ENSEMBL_GENE_ID	GENE_DESCRIPTION	ENSEMBL_MOUSE_GENE_ID	AGE(ANOVA_P-VALUE)	GENDER(ANOVA_P-VALUE	CORRELATION_WITH_AGE	P-VALUE_OF_CORRELATION	Benjamini & Hochberg_Corrected_correlation_p-valu
RPS27L<ILMN_1712678>	ENSG00000185088	ribosomal protein S27-like	ENSMUSG00000036781	4.54E-07	NS	0.464503792	2.41E-09	3.34E-07
ZMAT3<ILMN_1865764>	ENSG00000172667	zinc finger, matrin-type 3	ENSMUSG00000027663	1.55E-08	0.04800056	0.465432389	2.22E-09	3.34E-07
ZMAT3<ILMN_1654262>	ENSG00000172667	zinc finger, matrin-type 3	ENSMUSG00000027663	5.67E-08	NS	0.45810446	4.24E-09	3.91E-07
APOBEC3H<ILMN_166482	ENSG00000100298	apolipoprotein B mRNA editing enzyme, catalytic polypeptide	ENSMUSG00000009585	4.46E-05	NS	0.393452289	6.91E-07	4.79E-05
PCDH20<ILMN_1703572>	ENSG00000197991	uncharacterized LOC101926951	ENSMUSG00000050505	7.91E-05	0.00020877	-0.382467684	1.48E-06	8.20E-05
PCDH20<ILMN_1703572>	ENSG00000197991	protocadherin 20	ENSMUSG00000050505	7.91E-05	0.00020877	-0.382467684	1.48E-06	8.20E-05
DNM1<ILMN_1805200>	ENSG00000106976	dynamin 1	ENSMUSG00000026825	0.001951229	NS	-0.338845936	2.37E-05	0.00109415
RBPMS2<ILMN_1808238>	ENSG00000166831	RNA binding protein with multiple splicing 2	ENSMUSG00000032387	5.29E-05	0.012717105	0.332154695	3.50E-05	0.001211875
TTC14<ILMN_1696757>	ENSG00000163728	tetratricopeptide repeat domain 14	ENSMUSG00000027677	2.45E-05	NS	-0.33282766	3.36E-05	0.001211875
CALR<ILMN_1736256>	ENSG00000179218	calreticulin	ENSMUSG00000003814	0.0005132	NS	0.329036039	4.18E-05	0.00128251
TRIP6<ILMN_1713990>	ENSG00000087077	thyroid hormone receptor interactor 6	ENSMUSG00000023348	0.000793515	NS	0.327235823	4.63E-05	0.00128251
ZC3H12B<ILMN_1814629:	ENSG00000102053	zinc finger CCCH-type containing 12B	ENSMUSG00000035045	0.0006172	0.000237769	0.306673644	0.00014239	0.003585639
PDIA4<ILMN_1772118>	ENSG00000155660	protein disulfide isomerase family A, member 4	ENSMUSG00000025823	0.001045033	NS	0.294729518	0.000263547	0.006041263
SLTM<ILMN_1742224>	ENSG00000137776	SAFB-like, transcription modulator	ENSMUSG00000032212	0.001043924	NS	-0.293277069	0.000283525	0.006041263
C15orf52<ILMN_1775330:	ENSG00000188549	chromosome 15 open reading frame 52	ENSMUSG00000045838	0.014024276	NS	0.291591348	0.000308468	0.00610326
MAP4<ILMN_1734695>	ENSG00000047849	microtubule-associated protein 4	ENSMUSG00000032479	8.45E-05	NS	-0.288965804	0.000351396	0.006489113
CXorf57<ILMN_1703279>	ENSG00000147231	chromosome X open reading frame 57	ENSMUSG00000042498	0.014401348	NS	-0.287016391	0.000386777	0.006696077
EXOSC2<ILMN_1697736>	ENSG00000130713	exosome component 2	ENSMUSG00000039356	0.002076567	NS	-0.28354401	0.000458075	0.007049265
RPN1<ILMN_1660533>	ENSG00000163902	ribophorin I	ENSMUSG00000030062	0.007508032	NS	0.284311618	0.000441342	0.007049265
RPL15<ILMN_1762747>	ENSG00000174748	ribosomal protein L15	ENSMUSG00000012405	0.012511741	NS	-0.275094214	0.000685216	0.009076907
RBM14<ILMN_1700604>	ENSG00000239306	RNA binding motif protein 14	ENSMUSG00000006456	0.002285491	NS	-0.275003409	0.000688141	0.009076907
RBM12<ILMN_1797698>	ENSG00000244462	RNA binding motif protein 12	ENSMUSG00000089824	0.000159874	NS	-0.275074751	0.000685842	0.009076907
RPL28<ILMN_1673509>	ENSG00000108107	ribosomal protein L28	ENSMUSG00000090549	0.015563099	NS	-0.273252752	0.000746816	0.009403092
ZNF622<ILMN_1776109>	ENSG00000173545	zinc finger protein 622	ENSMUSG00000052253	0.000274071	0.044004521	-0.270631017	0.000843325	0.010156566
WDR33<ILMN_1716086>	ENSG00000136709	SFT2 domain containing 3	ENSMUSG00000024400	0.000520504	NS	-0.269551485	0.000886291	0.010229275
WDR33<ILMN_1716086>	ENSG00000136709	WD repeat domain 33	ENSMUSG00000024400	0.000520504	NS	-0.269551485	0.000886291	0.010229275
C1orf52<ILMN_1742611>	ENSG00000162642	chromosome 1 open reading frame 52	ENSMUSG00000036873	0.000596636	NS	-0.268382054	0.000935096	0.010360864
IPO5<ILMN_1690887>	ENSG00000065150	importin 5	ENSMUSG00000030662	0.003008832	NS	-0.263243789	0.00118011	0.01257271
S100A16<ILMN_1728049>	ENSG00000188643	S100 calcium binding protein A16	ENSMUSG00000074457	0.004578825	NS	0.262309434	0.001230518	0.012624203
SURF6<ILMN_1652642>	ENSG00000148296	surfeit 6	ENSMUSG00000036160	0.00538773	NS	-0.260870067	0.001312033	0.012761189
SURF6<ILMN_1652642>	ENSG00000261306	surfeit 6	NA	0.00538773	NS	-0.260870067	0.001312033	0.012761189
WDR33<ILMN_1670172>	ENSG00000136709	SFT2 domain containing 3	ENSMUSG00000024400	0.001144197	NS	-0.260462261	0.001336009	0.012761189
WDR33<ILMN_1670172>	ENSG00000136709	WD repeat domain 33	ENSMUSG00000024400	0.001144197	NS	-0.260462261	0.001336009	0.012761189
FXR1<ILMN_1679640>	ENSG00000114416	fragile X mental retardation, autosomal homolog 1	ENSMUSG00000027680	0.004000811	NS	-0.259543966	0.001391468	0.012847888
DAP3<ILMN_1666706>	ENSG00000132676	death associated protein 3	ENSMUSG00000068921	0.004988889	NS	-0.258654509	0.001447181	0.012931262
EIF2AK2<ILMN_1706502>	ENSG00000055332	eukaryotic translation initiation factor 2-alpha kinase 2	ENSMUSG00000024079	0.003883755	NS	0.254518524	0.001733949	0.015009496
NCOA5<ILMN_1770035>	ENSG00000124160	nuclear receptor coactivator 5	ENSMUSG00000039804	0.008752755	NS	-0.251499456	0.001974939	0.015630232
PCBP4<ILMN_1728498>	ENSG00000090097	poly(rC) binding protein 4	ENSMUSG00000023495	0.005798535	0.018421789	0.251908452	0.001940601	0.015630232
RPL37A<ILMN_1711222>	ENSG00000197756	ribosomal protein L37a	ENSMUSG00000046330	0.007538277	NS	-0.252578777	0.001885494	0.015630232
ANKRD17<ILMN_1712019	ENSG00000132466	ankyrin repeat domain 17	ENSMUSG00000055204	0.012029138	NS	-0.25018944	0.002088673	0.016071178
LSM4<ILMN_1788099>	ENSG00000130520	LSM4 homolog, U6 small nuclear RNA associated (S. cerevisia	ENSMUSG00000031848	0.001316649	NS	0.249339683	0.002165593	0.016212683
RPL22<ILMN_1653444>	ENSG00000116251	ribosomal protein L22	ENSMUSG00000028936	0.01747009	NS	-0.247576417	0.002333463	0.017009717
PURB<ILMN_1750079>	ENSG00000146676	purine-rich element binding protein B	ENSMUSG00000094483	0.006134984	0.040321488	-0.246652259	0.002426079	0.017231382
SON<ILMN_1668463>	ENSG00000159140	SON DNA binding protein	ENSMUSG00000022961	0.025843984	NS	-0.245267414	0.002571105	0.017804902
ZNF326<ILMN_1662021>	ENSG00000162664	zinc finger protein 326	ENSMUSG00000029290	0.003763663	NS	-0.244529723	0.002651521	0.017818473
RBMX2<ILMN_1678203>	ENSG00000134597	RNA binding motif protein, X-linked 2	ENSMUSG00000031107	0.01236272	NS	-0.244079446	0.002701718	0.017818473
PABPC5<ILMN_1771808>	ENSG00000174740	poly(A) binding protein, cytoplasmic 5	ENSMUSG00000034732	0.006815521	NS	-0.242828185	0.002845751	0.018331931
RPL5<ILMN_1725656>	ENSG00000122406	ribosomal protein L5	ENSMUSG00000058558	0.039592542	0.009856048	-0.242211693	0.002919237	0.018377924
RPL5<ILMN_1725656>	ENSG00000122406	small nucleolar RNA, C/D box 21	ENSMUSG00000058558	0.039592542	0.009856048	-0.242211693	0.002919237	0.018377924
NOMO2<ILMN_1652478>	ENSG00000185164	nodal modulator 1-like	ENSMUSG00000030835	0.00317089	NS	0.24070469	0.00310614	0.019120017
NOMO2<ILMN_1652478>	ENSG00000185164	NODAL modulator 2	ENSMUSG00000030835	0.00317089	NS	0.24070469	0.00310614	0.019120017
ANKHD1<ILMN_1765091>	ENSG00000131503	ankyrin repeat and KH domain containing 1	ENSMUSG00000024483	0.018125499	NS	-0.238877204	0.003347239	0.0201562
ANKHD1<ILMN_1765091>	ENSG00000131503	ANKHD1-EIF4EBP3 readthrough	ENSMUSG00000024483	0.018125499	NS	-0.238877204	0.003347239	0.0201562
OLA1<ILMN_1659820>	ENSG00000138430	Obg-like ATPase 1	ENSMUSG00000027108	0.005753395	NS	0.237694077	0.003512192	0.020699515
GAPDH<ILMN_1802252>	ENSG00000111640	glyceraldehyde-3-phosphate dehydrogenase	ENSMUSG00000081607	0.024786517	NS	0.236161973	0.003736667	0.021133975
SARS2<ILMN_1655684>	ENSG00000104835	seryl-tRNA synthetase 2, mitochondrial	ENSMUSG00000070699	0.001348125	NS	0.2361498	0.003738501	0.021133975
KIAA1429<ILMN_1813635	ENSG00000164944	KIAA1429	ENSMUSG00000040720	0.047600545	NS	-0.235506361	0.003836605	0.021254792
PAPD5<ILMN_1710571>	ENSG00000121274	PAP associated domain containing 5	ENSMUSG00000036779	0.031323078	NS	-0.233047323	0.004233216	0.022992173
DDX55<ILMN_1797425>	ENSG00000111364	DEAD (Asp-Glu-Ala-Asp) box polypeptide 55	ENSMUSG00000029389	0.012021183	NS	-0.230877581	0.004613323	0.024157593
UBE2D3<ILMN_1682443>	ENSG00000109332	ubiquitin-conjugating enzyme E2D 3	ENSMUSG00000078578	0.0048664	NS	-0.229887957	0.004796634	0.024157593
RBM17<ILMN_1781906>	ENSG00000134453	RNA binding motif protein 17	ENSMUSG00000037197	0.026063383	NS	-0.230616605	0.004661044	0.024157593
PDIA6<ILMN_1680626>	ENSG00000143870	protein disulfide isomerase family A, member 6	ENSMUSG00000020571	0.005194952	NS	0.229978656	0.004779566	0.024157593
WDR33<ILMN_1745852>	ENSG00000136709	SFT2 domain containing 3	ENSMUSG00000024400	0.00821367	NS	-0.228946483	0.004977053	0.024618637

WDR33<ILMN_1745852>	ENSG00000136709	WD repeat domain 33	ENSMUSG00000024400		0.00821367	NS		-0.228946483	0.004977053	0.024618637
RTN4<ILMN_1693598>	ENSG00000115310	reticulon 4	ENSMUSG00000020458		0.011339441	NS		-0.227123242	0.005343807	0.025969027
LYAR<ILMN_1764362>	ENSG00000145220	Ly1 antibody reactive	ENSMUSG00000067367		0.00908322	NS		-0.222757392	0.006321963	0.027682452
NAP1L4<ILMN_1804327>	ENSG00000205531	nucleosome assembly protein 1-like 4	ENSMUSG00000059119		0.002146452	NS		0.222725638	0.006329627	0.027682452
RNPC3<ILMN_1783026>	ENSG00000185946	RNA-binding region (RNP1, RRM) containing 3	ENSMUSG00000027981		0.030478352	NS		-0.224482802	0.005917788	0.027682452
LRP1<ILMN_1669772>	ENSG00000123384	low density lipoprotein receptor-related protein 1	ENSMUSG00000040249		0.007342596	NS		0.224656517	0.005878401	0.027682452
RBMY1A1<ILMN_1690753	ENSG00000234414	RNA binding motif protein, Y-linked, family 1, member A1	ENSMUSG00000094511		0.003541864	NS		-0.221643344	0.006595819	0.027682452
NUSAP1<ILMN_1726720>	ENSG00000137804	nucleolar and spindle associated protein 1	ENSMUSG00000027306	NS			0.015569372	0.222357948	0.006418968	0.027682452
LEMD3<ILMN_1727361>	ENSG00000174106	LEM domain containing 3	ENSMUSG00000048661		0.018173589	NS		-0.22323961	0.006206599	0.027682452
SAFB<ILMN_1722059>	ENSG00000160633	scaffold attachment factor B	ENSMUSG00000071054		0.015091904		0.030887551	-0.22189418	0.006533254	0.027682452
CRKL<ILMN_1690122>	ENSG00000099942	v-crk avian sarcoma virus CT10 oncogene homolog-like	ENSMUSG00000006134		0.004232218	NS		-0.221709974	0.006579148	0.027682452
EIF1B<ILMN_1679324>	ENSG00000114784	eukaryotic translation initiation factor 1B	ENSMUSG00000006941		0.006349545	NS		-0.220557007	0.006872979	0.028415152
PRPF4B<ILMN_1697168>	ENSG00000112739	PRP4 pre-mRNA processing factor 4 homolog B (yeast)	ENSMUSG00000021413		0.041231153	NS		-0.21845062	0.007440048	0.030307254
ISY1<ILMN_1662128>	ENSG00000240682	ISY1 splicing factor homolog (S. cerevisiae)	ENSMUSG00000030056		0.022261936	NS		-0.216448256	0.00801723	0.031278489
DHX36<ILMN_1738272>	ENSG00000174953	DEAH (Asp-Glu-Ala-His) box polypeptide 36	ENSMUSG00000027770		0.017167871	NS		-0.216586765	0.007976063	0.031278489
DNAJC21<ILMN_1711089	ENSG00000168724	DnaJ (Hsp40) homolog, subfamily C, member 21	ENSMUSG00000044224		0.034864037	NS		-0.216998078	0.007854918	0.031278489
HSPA1A<ILMN_1789074>	ENSG00000237724	heat shock 70kDa protein 1A	NA		0.021030363	NS		-0.215276395	0.008373124	0.032213269
HSPA1A<ILMN_1789074>	ENSG00000204389	heat shock 70kDa protein 1A	ENSMUSG00000090877		0.021030363	NS		-0.215276395	0.008373124	0.032213269
HSPA1A<ILMN_1789074>	ENSG00000237724	heat shock 70kDa protein 1B	NA		0.021030363	NS		-0.215276395	0.008373124	0.032213269
HSPA1A<ILMN_1789074>	ENSG00000234475	heat shock 70kDa protein 1B	NA		0.021030363	NS		-0.215276395	0.008373124	0.032213269
HSPA1A<ILMN_1789074>	ENSG00000215328	heat shock 70kDa protein 1B	NA		0.021030363	NS		-0.215276395	0.008373124	0.032213269
HSPA1A<ILMN_1789074>	ENSG00000234475	heat shock 70kDa protein 1A	NA		0.021030363	NS		-0.215276395	0.008373124	0.032213269
HSPA1A<ILMN_1789074>	ENSG00000235941	heat shock 70kDa protein 1A	NA		0.021030363	NS		-0.215276395	0.008373124	0.032213269
HSPA1A<ILMN_1789074>	ENSG00000204389	heat shock 70kDa protein 1B	ENSMUSG00000090877		0.021030363	NS		-0.215276395	0.008373124	0.032213269
HSPA1A<ILMN_1789074>	ENSG00000215328	heat shock 70kDa protein 1A	NA		0.021030363	NS		-0.215276395	0.008373124	0.032213269
HSPA1A<ILMN_1789074>	ENSG00000235941	heat shock 70kDa protein 1B	NA		0.021030363	NS		-0.215276395	0.008373124	0.032213269
ZCCHC9<ILMN_1723007>	ENSG00000131732	zinc finger, CCHC domain containing 9	ENSMUSG00000021621		0.013218555	NS		-0.213700673	0.00887369	0.033510142
SF3A3<ILMN_1705151>	ENSG00000183431	splicing factor 3a, subunit 3, 60kDa	ENSMUSG00000028902		0.03401645	NS		-0.213460799	0.008952168	0.033510142
RBM22<ILMN_1760256>	ENSG00000086589	RNA binding motif protein 22	ENSMUSG00000024604		0.04622209	NS		-0.212412118	0.009302508	0.034119665
R3HDM2<ILMN_1764091	ENSG00000179912	R3H domain containing 2	ENSMUSG00000025404		0.006909547	NS		-0.212239413	0.009361352	0.034119665
THUMPD2<ILMN_178751	ENSG00000138050	THUMP domain containing 2	ENSMUSG00000024246		0.048317142	NS		-0.210410481	0.010004958	0.035080683
CCDC59<ILMN_1662318>	ENSG00000133773	coiled-coil domain containing 59	ENSMUSG00000019897		0.008538995	NS		-0.211063838	0.009770695	0.035080683
SPEN<ILMN_1802611>	ENSG00000065526	spen homolog, transcriptional regulator (Drosophila)	ENSMUSG00000040761		0.004044591	NS		-0.210529871	0.009961785	0.035080683
TNRC6A<ILMN_1739573>	ENSG00000090905	trinucleotide repeat containing 6A	ENSMUSG00000052707	NS			0.028564128	-0.209565337	0.010315323	0.035275847
CEBPZ<ILMN_1744147>	ENSG00000115816	CCAAT/enhancer binding protein (C/EBP), zeta	ENSMUSG00000024081		0.016585165	NS		-0.209887352	0.010196082	0.035275847
MRPL40<ILMN_1687403>	ENSG00000185608	mitochondrial ribosomal protein L40	ENSMUSG00000022706		0.037792828	NS		-0.209201713	0.010451447	0.035305508
SAFB2<ILMN_1705679>	ENSG00000130254	scaffold attachment factor B2	ENSMUSG00000042625		0.008333817		0.001790623	-0.208203752	0.010833196	0.036154173
LBR<ILMN_1810418>	ENSG00000143815	lamin B receptor	ENSMUSG00000004880		0.023313487	NS		-0.206378999	0.011563033	0.038130468
GAPDH<ILMN_2038778>	ENSG00000111640	glyceraldehyde-3-phosphate dehydrogenase	ENSMUSG00000081607		0.043868168	NS		0.205044441	0.012123813	0.039509357
SON<ILMN_1772410>	ENSG00000159140	SON DNA binding protein	ENSMUSG00000022961		0.035983593	NS		-0.204477361	0.012369231	0.039840427
SORBS2<ILMN_1716407>	ENSG00000154556	sorbin and SH3 domain containing 2	ENSMUSG00000031626		0.010617832	NS		-0.203528819	0.012789479	0.040720528
MRPL39<ILMN_1777756>	ENSG00000154719	mitochondrial ribosomal protein L39	ENSMUSG00000022889		0.012101042	NS		-0.201533113	0.013714698	0.043170135
PHF5A<ILMN_1775901>	ENSG00000100410	PHD finger protein 5A	ENSMUSG00000061360		0.00797348	NS		-0.199555047	0.014688947	0.045717294
CCDC47<ILMN_1804522>	ENSG00000108588	coiled-coil domain containing 47	ENSMUSG00000078622		0.006572638	NS		-0.199113876	0.014914293	0.04590287
HSP90B1<ILMN_1686328	ENSG00000166598	heat shock protein 90kDa beta (Grp94), member 1	ENSMUSG00000020048		0.02205863	NS		0.198706986	0.015124795	0.046039196
HSP90B1<ILMN_1686328	ENSG00000166598	microRNA 3652	ENSMUSG00000020048		0.02205863	NS		0.198706986	0.015124795	0.046039196
TDRKH<ILMN_1792726>	ENSG00000182134	tudor and KH domain containing	ENSMUSG00000041912		0.001488625	NS		-0.198380911	0.015295352	0.046052304
NOL11<ILMN_1688971>	ENSG00000130935	nucleolar protein 11	ENSMUSG00000018433		0.015958922	NS		-0.197970761	0.015512264	0.046203183
NME1<ILMN_1741133>	ENSG00000239672	NME/NM23 nucleoside diphosphate kinase 1	ENSMUSG00000090801		0.007475552	NS		0.195342464	0.016966902	0.048497951
CSTF2T<ILMN_1807515>	ENSG00000177613	cleavage stimulation factor, 3' pre-RNA, subunit 2, 64kDa, tau	ENSMUSG00000053536		0.006109479	NS		-0.195011671	0.017158121	0.048497951
FAM120C<ILMN_1758476	ENSG00000184083	family with sequence similarity 120C	ENSMUSG00000025262		0.012094371	NS		-0.195570433	0.016836201	0.048497951
FAM120C<ILMN_1758476	ENSG00000268158	family with sequence similarity 120C	NA		0.012094371	NS		-0.195570433	0.016836201	0.048497951
CDKN2A<ILMN_1717714>	LRG_11	cyclin-dependent kinase inhibitor 2A	NA		0.008566347	NS		0.195897086	0.016650448	0.048497951
CDKN2A<ILMN_1717714>	ENSG00000147889	cyclin-dependent kinase inhibitor 2A	NA		0.008566347	NS		0.195897086	0.016650448	0.048497951
EIF4G3<ILMN_1775692>	ENSG00000075151	eukaryotic translation initiation factor 4 gamma, 3	ENSMUSG00000028760		0.022488963	NS		0.19526589	0.017011001	0.048497951
PTCD2<ILMN_1677221>	ENSG00000049883	pentatricopeptide repeat domain 2	ENSMUSG00000021650		0.01827982	NS		-0.194031158	0.017735952	0.04962483
ACTN4<ILMN_1725534>	ENSG00000130402	actinin, alpha 4	ENSMUSG00000054808		0.045932338	NS		0.193497419	0.018057531	0.050019358
HARS2<ILMN_1774432>	ENSG00000112855	histidyl-tRNA synthetase 2, mitochondrial	ENSMUSG00000019143		0.021499297	NS		0.192385921	0.018743467	0.050407196
WDR43<ILMN_1671442>	ENSG00000163811	WD repeat domain 43	ENSMUSG00000041057		0.02548454	NS		-0.19278876	0.018492301	0.050407196
HIST1H1C<ILMN_1757406	ENSG00000187837	histone cluster 1, H1c	ENSMUSG00000036181		0.029188245	NS		0.192590919	0.018615287	0.050407196
HSPA1B<ILMN_1660436>	ENSG00000231555	heat shock 70kDa protein 1B	NA		0.033304615	NS		-0.19182007	0.019101254	0.050875445
HSPA1B<ILMN_1660436>	ENSG00000212866	heat shock 70kDa protein 1A	NA		0.033304615	NS		-0.19182007	0.019101254	0.050875445
HSPA1B<ILMN_1660436>	ENSG00000231555	heat shock 70kDa protein 1A	NA		0.033304615	NS		-0.19182007	0.019101254	0.050875445
HSPA1B<ILMN_1660436>	ENSG00000232804	heat shock 70kDa protein 1A	NA		0.033304615	NS		-0.19182007	0.019101254	0.050875445
HSPA1B<ILMN_1660436>	ENSG00000224501	heat shock 70kDa protein 1B	NA		0.033304615	NS		-0.19182007	0.019101254	0.050875445
HSPA1B<ILMN_1660436>	ENSG00000212866	heat shock 70kDa protein 1B	NA		0.033304615	NS		-0.19182007	0.019101254	0.050875445
HSPA1B<ILMN_1660436>	ENSG00000204388	heat shock 70kDa protein 1A	ENSMUSG00000090877		0.033304615	NS		-0.19182007	0.019101254	0.050875445

HSPA1B<ILMN_1660436>	ENSG00000204388	heat shock 70kDa protein 1B	ENSMUSG00000090877		0.033304615	NS		-0.19182007	0.019101254	0.050875445
HSPA1B<ILMN_1660436>	ENSG00000224501	heat shock 70kDa protein 1A	NA		0.033304615	NS		-0.19182007	0.019101254	0.050875445
HSPA1B<ILMN_1660436>	ENSG00000232804	heat shock 70kDa protein 1B	NA		0.033304615	NS		-0.19182007	0.019101254	0.050875445
NOMO1<ILMN_1678730>	ENSG00000103512	NODAL modulator 1	ENSMUSG00000030835		0.010040705	NS		0.191100179	0.019564971	0.051614254
NOMO1<ILMN_1678730>	ENSG00000103512	nodal modulator 1-like	ENSMUSG00000030835		0.010040705	NS		0.191100179	0.019564971	0.051614254
NASP<ILMN_1725840>	ENSG00000132780	nuclear autoantigenic sperm protein (histone-binding)	ENSMUSG00000028693	NS			0.049234537	-0.1897469	0.02046307	0.053474249
C11orf68<ILMN_1757847:	ENSG00000175573	chromosome 11 open reading frame 68	ENSMUSG00000047423		0.035028499	NS		0.189430619	0.020678027	0.053530975
CDC40<ILMN_1737651>	ENSG00000168438	cell division cycle 40	ENSMUSG00000038446		0.021533955	NS		-0.189059969	0.020932413	0.053657314
RDX<ILMN_1708611>	ENSG00000137710	radixin	ENSMUSG00000032050		0.037559664	NS		-0.188797395	0.021114253	0.053657314
HSPA8<ILMN_1686367>	ENSG00000109971	small nucleolar RNA, C/D box 14D	ENSMUSG00000015656		0.03599468	NS		-0.188244247	0.021501784	0.054012111
HSPA8<ILMN_1686367>	ENSG00000109971	heat shock 70kDa protein 8	ENSMUSG00000015656		0.03599468	NS		-0.188244247	0.021501784	0.054012111
HSPA8<ILMN_1686367>	ENSG00000109971	small nucleolar RNA, C/D box 14C	ENSMUSG00000015656		0.03599468	NS		-0.188244247	0.021501784	0.054012111
ZC3H7A<ILMN_1693227>	ENSG00000122299	zinc finger CCCH-type containing 7A	ENSMUSG00000037965		0.015490016	NS		-0.186877391	0.022485777	0.054012111
KHDRBS3<ILMN_1691747:	ENSG00000131773	KH domain containing, RNA binding, signal transduction asso	ENSMUSG00000022332		0.013191679	NS		-0.18710582	0.022318684	0.054012111
MFAP1<ILMN_1676799>	ENSG00000140259	microfibrillar-associated protein 1	ENSMUSG00000068479		0.014981018	NS		-0.186696593	0.022618789	0.054012111
DAP3<ILMN_1781680>	ENSG00000132676	death associated protein 3	ENSMUSG00000068921		0.012326862	NS		0.187794635	0.021821281	0.054012111
MRPS31<ILMN_1654552>	ENSG00000102738	mitochondrial ribosomal protein S31	ENSMUSG00000031533		0.004020531	NS		-0.186709804	0.022609047	0.054012111
CSTF1<ILMN_1682198>	ENSG00000101138	cleavage stimulation factor, 3' pre-RNA, subunit 1, 50kDa	ENSMUSG00000027498		0.004026488	NS		-0.187216637	0.022238009	0.054012111
SF3A1<ILMN_1697286>	ENSG00000099995	splicing factor 3a, subunit 1, 120kDa	ENSMUSG00000002129		0.010802275	NS		-0.183961496	0.024715073	0.058513456
RPL8<ILMN_1764721>	ENSG00000161016	ribosomal protein L8	ENSMUSG00000003970	NS			0.049057731	-0.183342632	0.025211851	0.058686407
RBMX<ILMN_1723580>	ENSG00000147274	uncharacterized LOC101928747	ENSMUSG00000091987		0.020767258	NS		-0.183362555	0.025195727	0.058686407
RBMX<ILMN_1723580>	ENSG00000147274	small nucleolar RNA, C/D box 61	ENSMUSG00000091987		0.020767258	NS		-0.183362555	0.025195727	0.058686407
RBMX<ILMN_1723580>	ENSG00000147274	RNA binding motif protein, X-linked	ENSMUSG00000091987		0.020767258	NS		-0.183362555	0.025195727	0.058686407
SLC25A11<ILMN_1664168	ENSG00000108528	solute carrier family 25 (mitochondrial carrier; oxoglutarate c	ENSMUSG00000014606		0.018024285	NS		0.181611648	0.02664702	0.061510205
DDX17<ILMN_1764285>	ENSG00000100201	DEAD (Asp-Glu-Ala-Asp) box helicase 17	ENSMUSG00000055065		0.039514977	NS		-0.181226644	0.026975556	0.061753968
GNB2L1<ILMN_1736500>	ENSG00000204628	small nucleolar RNA, C/D box 96A	ENSMUSG00000020372	NS			0.020456468	-0.180572949	0.0275413	0.062532296
GNB2L1<ILMN_1736500>	ENSG00000204628	guanine nucleotide binding protein (G protein), beta polypep	ENSMUSG00000020372	NS			0.020456468	-0.180572949	0.0275413	0.062532296
GNB2L1<ILMN_1736500>	ENSG00000204628	small nucleolar RNA, C/D box 95	ENSMUSG00000020372	NS			0.020456468	-0.180572949	0.0275413	0.062532296
ZAK<ILMN_1768110>	NA	NA	NA		0.000601189		8.25E-07	-0.179932836	0.028105075	0.062990671
CNN3<ILMN_1782439>	ENSG00000117519	calponin 3, acidic	ENSMUSG00000053931		0.020619695	NS		-0.179828396	0.028197988	0.062990671
GLTSCR2<ILMN_1703565>	ENSG00000105373	small nucleolar RNA, C/D box 23	ENSMUSG00000041560	NS			0.016215983	-0.177591852	0.030251468	0.066505216
GLTSCR2<ILMN_1703565>	ENSG00000105373	glioma tumor suppressor candidate region gene 2	ENSMUSG00000041560	NS			0.016215983	-0.177591852	0.030251468	0.066505216
MRPL27<ILMN_1811327>	ENSG00000108826	mitochondrial ribosomal protein L27	ENSMUSG00000024414		0.020403023	NS		0.177774187	0.030079426	0.066505216
PRPF38A<ILMN_1656274>	ENSG00000134748	PRP38 pre-mRNA processing factor 38 (yeast) domain contair	ENSMUSG00000063800	NS			0.048210065	-0.17590163	0.031886497	0.06954772
G3BP2<ILMN_1720422>	ENSG00000138757	GTPase activating protein (SH3 domain) binding protein 2	ENSMUSG00000029405		0.020049384	NS		-0.17491409	0.032876048	0.071145827
CAPRIN1<ILMN_1770676>	ENSG00000135387	cell cycle associated protein 1	ENSMUSG00000027184		0.048243419	NS		-0.174534334	0.033263445	0.071426168
TCF25<ILMN_1814971>	ENSG00000141002	transcription factor 25 (basic helix-loop-helix)	ENSMUSG00000001472	NS			4.61E-05	0.172925826	0.034947458	0.074464972
NAF1<ILMN_1679891>	ENSG00000145414	nuclear assembly factor 1 ribonucleoprotein	ENSMUSG00000014907		0.014996533	NS		-0.171990538	0.035959371	0.076036225
MPHOSPH10<ILMN_1652(	ENSG00000124383	M-phase phosphoprotein 10 (U3 small nucleolar ribonucleop	ENSMUSG00000030521		0.034687924	NS		-0.16884574	0.039544969	0.08298452
IGF2BP2<ILMN_1702447>	ENSG00000073792	insulin-like growth factor 2 mRNA binding protein 2	ENSMUSG00000033581		0.017599893		0.035949664	-0.167081353	0.041685023	0.086817673
ALDH6A1<ILMN_1785284	ENSG00000119711	aldehyde dehydrogenase 6 family, member A1	ENSMUSG00000021238		0.027104333	NS		-0.166794929	0.042041424	0.086906517
SND1<ILMN_1767969>	ENSG00000197157	staphylococcal nuclease and tudor domain containing 1	ENSMUSG00000001424		0.006545007	NS		0.165103391	0.044198649	0.090689082
NOL10<ILMN_1775011>	ENSG00000115761	nucleolar protein 10	ENSMUSG00000061458		0.037058202	NS		-0.161171504	0.049572151	0.100966806
ZCCHC8<ILMN_1775793>	ENSG00000033030	zinc finger, CCHC domain containing 8	ENSMUSG00000029427	NS			0.034822729	-0.160509706	0.050527858	0.102162169
FASTKD3<ILMN_1750160>	ENSG00000124279	FAST kinase domains 3	ENSMUSG00000021532		0.043444744		0.012621584	-0.159713506	0.051697857	0.103770342
NFX1<ILMN_1804610>	ENSG00000086102	nuclear transcription factor, X-box binding 1	ENSMUSG00000028423		0.031634298	NS		-0.158825622	0.053028952	0.105598751
MATR3<ILMN_1661673>	ENSG00000015479	small nucleolar RNA host gene 4 (non-protein coding)	ENSMUSG00000037236	NS			1.00E-05	-0.158600306	0.053371213	0.105598751
MATR3<ILMN_1661673>	ENSG00000015479	matrin 3	ENSMUSG00000037236	NS			1.00E-05	-0.158600306	0.053371213	0.105598751
RPL6<ILMN_1690494>	ENSG00000089009	ribosomal protein L6	ENSMUSG00000029614	NS			0.036929715	-0.158196486	0.053989195	0.10606387
TBCA<ILMN_1726239>	ENSG00000171530	tubulin folding cofactor A	ENSMUSG00000042043	NS			0.010617081	-0.157343075	0.055314659	0.10788102
SMG1<ILMN_1801383>	ENSG00000157106	SMG1 phosphatidylinositol 3-kinase-related kinase	ENSMUSG00000030655	NS			0.004300756	-0.157102521	0.055693085	0.10788102
SMG1<ILMN_1801383>	ENSG00000157106	bolA family member 2	ENSMUSG00000030655	NS			0.004300756	-0.157102521	0.055693085	0.10788102
SMG1<ILMN_1801383>	ENSG00000157106	serine/threonine-protein kinase SMG1-like	ENSMUSG00000030655	NS			0.004300756	-0.157102521	0.055693085	0.10788102
TDRD10<ILMN_1751630>	ENSG00000163239	tudor domain containing 10	NA		0.035169434	NS		0.15635944	0.056875562	0.109406459
EED<ILMN_1796813>	ENSG00000074266	embryonic ectoderm development	ENSMUSG00000030619		0.03171655	NS		-0.156040188	0.057389903	0.109634499
RBMY1D<ILMN_1676960>	ENSG00000244395	RNA binding motif protein, Y-linked, family 1, member D	ENSMUSG00000093918	NS			0.045458786	-0.155562912	0.058165969	0.110355984
RBMY1D<ILMN_1676960>	ENSG00000244395	RNA binding motif protein, Y-linked, family 1, member B	ENSMUSG00000093918	NS			0.045458786	-0.155562912	0.058165969	0.110355984
RBMY1D<ILMN_1676960>	ENSG00000244395	RNA binding motif protein, Y-linked, family 1, member A1	ENSMUSG00000093918	NS			0.045458786	-0.155562912	0.058165969	0.110355984
TARDBP<ILMN_1677532>	ENSG00000120948	TAR DNA binding protein	ENSMUSG00000041459		0.044702414	NS		-0.155003001	0.05908738	0.111341526
HMGB3<ILMN_1733519>	ENSG00000269411	high mobility group box 3	NA	NS			0.045385	0.154751865	0.059504539	0.111369984
HMGB3<ILMN_1733519>	ENSG00000029993	high mobility group box 3	ENSMUSG00000015217	NS			0.045385	0.154751865	0.059504539	0.111369984
TUFM<ILMN_1738369>	ENSG00000178952	Tu translation elongation factor, mitochondrial	ENSMUSG00000030735		0.043100157	NS		0.150084401	0.067707727	0.12587276
LSM11<ILMN_1702698>	ENSG00000155858	LSM11, U7 small nuclear RNA associated	ENSMUSG00000044847		0.00789442	NS		-0.149396299	0.068991501	0.126336839
SLC16A3<ILMN_1808982>	ENSG00000141526	solute carrier family 16 (monocarboxylate transporter), mem	ENSMUSG00000025161	NS			0.028197305	0.149138742	0.06947708	0.126336839
RPL3<ILMN_1678090>	ENSG00000100316	RNA, U86 small nucleolar	ENSMUSG00000060036	NS			0.043834981	-0.148977903	0.069781719	0.126336839
RPL3<ILMN_1678090>	ENSG00000100316	small nucleolar RNA, C/D box 83B	ENSMUSG00000060036	NS			0.043834981	-0.148977903	0.069781719	0.126336839
RPL3<ILMN_1678090>	ENSG00000100316	ribosomal protein L3	ENSMUSG00000060036	NS			0.043834981	-0.148977903	0.069781719	0.126336839

KIAA1429<ILMN_1741841	ENSG00000164944	KIAA1429	ENSMUSG00000040720	NS			0.037232456	-0.149394254	0.068995345	0.126336839
MBNL1<ILMN_1807304>	ENSG00000152601	muscleblind-like splicing regulator 1	ENSMUSG00000027763		0.030348318	NS		-0.148368824	0.070945208	0.126785956
C1orf131<ILMN_1805474	ENSG00000143633	chromosome 1 open reading frame 131	ENSMUSG00000031984	NS			0.045051222	-0.148421417	0.070844125	0.126785956
RPS10<ILMN_1686954>	ENSG00000124614	ribosomal protein S10	ENSMUSG00000052146	NS			0.012868519	-0.147205575	0.073211023	0.129168488
APEH<ILMN_1718023>	ENSG00000164062	acylaminoacyl-peptide hydrolase	ENSMUSG00000032590		0.025849091	NS		0.147291823	0.073041039	0.129168488
SF3B1<ILMN_1813603>	ENSG00000115524	splicing factor 3b, subunit 1, 155kDa	ENSMUSG00000025982		0.015293306	NS		-0.146395788	0.074822676	0.131176471
UBE2D2<ILMN_1725644>	ENSG00000131508	ubiquitin-conjugating enzyme E2D 2	ENSMUSG00000091896		0.036827251	NS		-0.142171445	0.083700829	0.145818427
CCT4<ILMN_1776073>	ENSG00000115484	chaperonin containing TCP1, subunit 4 (delta)	ENSMUSG00000007739		0.046106731	NS		-0.139754081	0.089149401	0.153562761
CPEB1<ILMN_1693345>	ENSG00000214575	cytoplasmic polyadenylation element binding protein 1	ENSMUSG00000025586		0.049825279	NS		0.139708459	0.089254885	0.153562761
RPS19<ILMN_1784717>	ENSG00000105372	ribosomal protein S19	ENSMUSG00000040952		0.023023612		6.78E-05	0.136296222	0.09743058	0.166594263
RPS19<ILMN_1784717>	ENSG00000272852	ribosomal protein S19	NA		0.023023612		6.78E-05	0.136296222	0.09743058	0.166594263
PUS7<ILMN_1779353>	ENSG00000091127	pseudouridylate synthase 7 homolog (S. cerevisiae)	ENSMUSG00000057541	NS			0.019749502	-0.133824659	0.103714103	0.174731096
CNOT4<ILMN_1794324>	ENSG00000080802	CCR4-NOT transcription complex, subunit 4	ENSMUSG00000038784		0.0352932	NS		-0.133836755	0.103682592	0.174731096
ZNF326<ILMN_1753950>	ENSG00000162664	zinc finger protein 326	ENSMUSG00000029290		0.023268977	NS		-0.133683774	0.104081663	0.174731096
TBL2<ILMN_1764489>	ENSG00000106638	transducin (beta)-like 2	ENSMUSG00000005374		0.037789803	NS		0.133317983	0.105040772	0.175278925
TBL2<ILMN_1764489>	ENSG00000263049	transducin (beta)-like 2	NA		0.037789803	NS		0.133317983	0.105040772	0.175278925
XRCC5<ILMN_1754919>	ENSG00000079246	X-ray repair complementing defective repair in Chinese hams	ENSMUSG00000026187	NS			0.014031137	-0.132505755	0.107195265	0.177802983
RBMS2<ILMN_1755411>	ENSG00000076067	RNA binding motif, single stranded interacting protein 2	ENSMUSG00000040043	NS			0.01984762	0.129819166	0.114569186	0.188902788
MRPS27<ILMN_1711414>	ENSG00000113048	mitochondrial ribosomal protein S27	ENSMUSG00000041632		0.032896964		0.047414605	0.128761991	0.117576891	0.192714801
NMD3<ILMN_1727348>	ENSG00000169251	NMD3 ribosome export adaptor	ENSMUSG00000027787	NS			0.029319563	-0.127225112	0.122058316	0.19888323
RPS7<ILMN_1750722>	ENSG00000171863	ribosomal protein S7	ENSMUSG00000061477		0.046297975	NS		0.126096229	0.1254334	0.203187437
SAP18<ILMN_1752793>	ENSG00000150459	Sin3A-associated protein, 18kDa	ENSMUSG00000061104	NS			0.00237838	-0.125191583	0.128189664	0.20644504
UBC<ILMN_1343290>	ENSG00000150991	ubiquitin C	ENSMUSG00000008348	NS			0.006093888	0.123433029	0.133680663	0.214043664
PARP10<ILMN_1710844>	ENSG00000178685	poly (ADP-ribose) polymerase family, member 10	ENSMUSG00000063268		0.030881446	NS		0.122766979	0.135806766	0.216198182
PARP10<ILMN_1710844>	ENSG00000261660	poly (ADP-ribose) polymerase family, member 10	NA		0.030881446	NS		0.122766979	0.135806766	0.216198182
PABPC4<ILMN_1754865>	ENSG00000090621	poly(A) binding protein, cytoplasmic 4 (inducible form)	ENSMUSG00000062093	NS			0.041489583	-0.121821146	0.138870251	0.219811846
PABPC4<ILMN_1754865>	ENSG00000090621	polyadenylate-binding protein 4-like	ENSMUSG00000062093	NS			0.041489583	-0.121821146	0.138870251	0.219811846
SEC61A1<ILMN_1696952>	ENSG00000058262	Sec61 alpha 1 subunit (S. cerevisiae)	ENSMUSG00000030082		0.049551037	NS		0.121376203	0.140329482	0.220859497
HERC5<ILMN_1729749>	ENSG00000138646	HECT and RLD domain containing E3 ubiquitin protein ligase 5	NA	NS			0.000393904	-0.117901082	0.152130824	0.238080405
CPSF4<ILMN_1660426>	ENSG00000160917	cleavage and polyadenylation specific factor 4, 30kDa	ENSMUSG00000029625	NS			0.009851609	-0.115246114	0.161639563	0.251540276
TWISTNB<ILMN_1781121	ENSG00000105849	TWIST neighbor	ENSMUSG00000020561		0.033748471	NS		-0.113993735	0.166276159	0.254466892
ZC3H8<ILMN_1666530>	ENSG00000144161	zinc finger CCCH-type containing 8	ENSMUSG00000027387	NS			0.032977271	-0.113996354	0.166266362	0.254466892
KIAA1324<ILMN_1771482	ENSG00000116299	KIAA1324	ENSMUSG00000040412	NS			0.046773049	-0.114034463	0.166123833	0.254466892
MRPL2<ILMN_1763264>	ENSG00000112651	mitochondrial ribosomal protein L2	ENSMUSG00000002767	NS			0.004178157	-0.113530191	0.168017171	0.255718486
SRP54<ILMN_1753862>	ENSG00000100883	signal recognition particle 54kDa	ENSMUSG00000073079	NS			0.005781477	0.113082034	0.169713241	0.256888286
RPS5<ILMN_1707810>	ENSG00000083845	ribosomal protein S5	ENSMUSG00000012848	NS			0.035725473	-0.112160271	0.173241571	0.26080393
ZCCHC7<ILMN_1744980>	ENSG00000147905	zinc finger, CCHC domain containing 7	ENSMUSG00000035649	NS			0.049285283	-0.111080575	0.177443104	0.265685074
MRPL9<ILMN_1795346>	ENSG00000143436	mitochondrial ribosomal protein L9	ENSMUSG00000028140		0.029434678	NS		-0.11035992	0.180288956	0.268494909
NOVA1<ILMN_1811363>	ENSG00000139910	neuro-oncological ventral antigen 1	ENSMUSG00000021047	NS			0.022678476	-0.106701013	0.195256884	0.289230809
NFX1<ILMN_1698402>	ENSG00000086102	nuclear transcription factor, X-box binding 1	ENSMUSG00000028423	NS			0.033017781	-0.105555295	0.20012422	0.294863848
CSTF3<ILMN_1808779>	ENSG00000176102	cleavage stimulation factor, 3' pre-RNA, subunit 3, 77kDa	ENSMUSG00000027176	NS			0.003834312	-0.104121403	0.206338557	0.30127351
LGALS1<ILMN_1723978>	ENSG00000100097	lectin, galactoside-binding, soluble, 1	ENSMUSG00000068220	NS			3.20E-06	-0.104050439	0.206649669	0.30127351
YBX2<ILMN_1755354>	ENSG00000006047	Y box binding protein 2	ENSMUSG00000018554	NS			0.01056652	-0.103124262	0.210741053	0.305629763
NIP7<ILMN_1704305>	ENSG00000132603	NIP7, nucleolar pre-rRNA processing protein	ENSMUSG00000031917	NS			0.016257483	-0.100992221	0.220378983	0.31794262
RPS18<ILMN_1753534>	ENSG00000223367	ribosomal protein S18	NA	NS			0.043286755	-0.098120393	0.233849848	0.335628988
RPS18<ILMN_1753534>	ENSG00000096150	ribosomal protein S18	NA	NS			0.043286755	-0.098120393	0.233849848	0.335628988
RPS18<ILMN_1753534>	ENSG00000231500	ribosomal protein S18	ENSMUSG00000008668	NS			0.043286755	-0.098120393	0.233849848	0.335628988
RPS18<ILMN_1753534>	ENSG00000227794	ribosomal protein S18	NA	NS			0.043286755	-0.098120393	0.233849848	0.335628988
RPS18<ILMN_1753534>	ENSG00000235650	ribosomal protein S18	NA	NS			0.043286755	-0.098120393	0.233849848	0.335628988
RPS18<ILMN_1753534>	ENSG00000226225	ribosomal protein S18	NA	NS			0.043286755	-0.098120393	0.233849848	0.335628988
LARP1<ILMN_1692770>	ENSG00000155506	La ribonucleoprotein domain family, member 1	ENSMUSG00000037331	NS			0.011690538	-0.096454065	0.241925765	0.345430137
DUT<ILMN_1721008>	ENSG00000128951	deoxyuridine triphosphatase	ENSMUSG00000027203	NS			0.003051734	-0.095867302	0.244815237	0.34776313
PRKRA<ILMN_1758474>	ENSG00000180228	protein kinase, interferon-inducible double stranded RNA dep	ENSMUSG00000002731	NS			0.027054605	-0.093589404	0.256259334	0.362162378
TSFM<ILMN_1685235>	ENSG00000123297	Ts translation elongation factor, mitochondrial	ENSMUSG00000040521	NS			0.002138563	0.089480226	0.277821915	0.390642976
DAZ3<ILMN_1754528>	ENSG00000187191	deleted in azoospermia 3	ENSMUSG00000010592	NS			0.001311012	-0.087755332	0.287227416	0.401828231
DAZ3<ILMN_1754528>	ENSG00000187191	deleted in azoospermia 2	ENSMUSG00000010592	NS			0.001311012	-0.087755332	0.287227416	0.401828231
RBM43<ILMN_1858599>	ENSG00000184898	RNA binding motif protein 43	ENSMUSG00000036249	NS			0.019289599	0.087277069	0.289872515	0.403490867
ELAVL3<ILMN_1678618>	ENSG00000196361	ELAV (embryonic lethal, abnormal vision, Drosophila)-like 3 (I	ENSMUSG00000003410	NS			0.030057163	0.084896134	0.303281551	0.420045016
ELAVL3<ILMN_1678618>	ENSG00000196361	formin-like protein 5-like	ENSMUSG00000003410	NS			0.030057163	0.084896134	0.303281551	0.420045016
BOLL<ILMN_1781833>	ENSG00000152430	bol, boule-like (Drosophila)	ENSMUSG00000025977	NS			2.92E-06	-0.08321304	0.313002807	0.431352117
UBE2I<ILMN_1725529>	ENSG00000103275	ubiquitin-conjugating enzyme E2I	ENSMUSG00000015120	NS			0.024360903	-0.082120567	0.319420231	0.438016809
SLC3A2<ILMN_1726456>	ENSG00000168003	solute carrier family 3 (amino acid transporter heavy chain), r	ENSMUSG00000010095	NS			0.044102023	-0.080138256	0.331280756	0.452043259
RPL29<ILMN_1737517>	ENSG00000162244	ribosomal protein L29	ENSMUSG00000074516	NS			0.045691292	0.076483219	0.35387828	0.480511221
ZAK<ILMN_1698803>	NA	NA	NA	NS			6.65E-05	-0.074015876	0.369664887	0.499498426
NBPF10<ILMN_1730957>	ENSG00000163386	neuroblastoma breakpoint family, member 14	NA	NS			7.15E-05	0.072750938	0.377923598	0.508169614
NBPF10<ILMN_1730957>	ENSG00000163386	neuroblastoma breakpoint family, member 10	NA	NS			7.15E-05	0.072750938	0.377923598	0.508169614
NBPF10<ILMN_1730957>	ENSG00000163386	neuroblastoma breakpoint family member 21-like	NA	NS			7.15E-05	0.072750938	0.377923598	0.508169614

NUFIP1<ILMN_1813800>	ENSG00000083635	nuclear fragile X mental retardation protein interacting prote	ENSMUSG00000022009	NS			0.010693428	-0.072473284	0.379751334	0.508169614
HARS2<ILMN_1718963>	ENSG00000112855	histidyl-tRNA synthetase 2, mitochondrial	ENSMUSG00000019143	NS			0.010024171	-0.071306019	0.387493838	0.508700391
RPS8<ILMN_1656203>	ENSG00000142937	small nucleolar RNA, C/D box 38B	ENSMUSG00000047675	NS			0.004873379	-0.07144329	0.386578399	0.508700391
RPS8<ILMN_1656203>	ENSG00000142937	ribosomal protein S8	ENSMUSG00000047675	NS			0.004873379	-0.07144329	0.386578399	0.508700391
RPS8<ILMN_1656203>	ENSG00000142937	small nucleolar RNA, C/D box 55	ENSMUSG00000047675	NS			0.004873379	-0.07144329	0.386578399	0.508700391
API5<ILMN_1815051>	ENSG00000166181	apoptosis inhibitor 5	ENSMUSG00000027193	NS			0.047378305	-0.071994657	0.382914622	0.508700391
RPS4X<ILMN_1810577>	ENSG00000198034	ribosomal protein S4, X-linked	ENSMUSG00000031320	NS			2.43E-20	-0.071499095	0.386206621	0.508700391
SMC1A<ILMN_1652006>	ENSG00000269384	structural maintenance of chromosomes 1A	NA	NS			0.000800554	0.069952679	0.396588836	0.518184423
SMC1A<ILMN_1652006>	ENSG00000072501	structural maintenance of chromosomes 1A	ENSMUSG00000041133	NS			0.000800554	0.069952679	0.396588836	0.518184423
NME1<ILMN_1713875>	ENSG00000239672	NME/NM23 nucleoside diphosphate kinase 1	ENSMUSG00000090801	NS			0.041993787	0.068884769	0.403854843	0.525200843
UBC<ILMN_943471>	ENSG00000150991	ubiquitin C	ENSMUSG00000008348	NS			0.046201005	0.068342386	0.407575189	0.527562292
CDC42EP4<ILMN_174522	ENSG00000179604	CDC42 effector protein (Rho GTPase binding) 4	ENSMUSG00000041598	NS			0.037804826	-0.066922493	0.417409875	0.537779267
RPGR<ILMN_1768097>	ENSG00000156313	retinitis pigmentosa GTPase regulator	ENSMUSG00000031174		0.033466355	NS		-0.064874196	0.431838297	0.553792635
SRP72<ILMN_1683888>	ENSG00000174780	signal recognition particle 72kDa	ENSMUSG00000036323	NS			0.042330259	-0.064407426	0.435165835	0.555488141
PUM1<ILMN_1783424>	ENSG00000134644	pumilio RNA-binding family member 1	ENSMUSG00000028580	NS			0.01874998	-0.062100305	0.451826543	0.571488313
LENG9<ILMN_1732720>	ENSG00000261906	leukocyte receptor cluster (LRC) member 9	NA	NS			0.029374222	0.062292616	0.450424284	0.571488313
LENG9<ILMN_1732720>	ENSG00000182909	leukocyte receptor cluster (LRC) member 9	ENSMUSG00000043432	NS			0.029374222	0.062292616	0.450424284	0.571488313
DDX3X<ILMN_1794392>	ENSG00000215301	DEAD (Asp-Glu-Ala-Asp) box helicase 3, X-linked	ENSMUSG00000039224	NS			3.71E-07	-0.059529951	0.470801346	0.592781637
BST2<ILMN_1723480>	ENSG00000130303	bone marrow stromal cell antigen 2	ENSMUSG00000046718	NS			0.000497954	0.05691537	0.490540877	0.614840857
C4BPA<ILMN_1810752>	ENSG00000123838	complement component 4 binding protein, alpha	ENSMUSG00000026405	NS			0.009512423	0.05557386	0.500837337	0.624918613
RBM24<ILMN_1673941>	ENSG00000112183	RNA binding motif protein 24	ENSMUSG00000038132	NS			0.015082344	-0.054708836	0.507536257	0.630437467
ZNF239<ILMN_1748427>	ENSG00000196793	zinc finger protein 239	ENSMUSG00000042097	NS			0.004265079	-0.054191686	0.511563301	0.632602831
RPS2<ILMN_1737074>	ENSG00000140988	ribosomal protein S2	ENSMUSG00000095427	NS			0.00610935	-0.053252455	0.518919067	0.63884707
RPS2<ILMN_1737074>	ENSG00000140988	small nucleolar RNA, H/ACA box 64	ENSMUSG00000095427	NS			0.00610935	-0.053252455	0.518919067	0.63884707
DDX43<ILMN_1813893>	ENSG00000080007	DEAD (Asp-Glu-Ala-Asp) box polypeptide 43	ENSMUSG00000070291	NS			2.73E-06	-0.052080333	0.528174021	0.640205908
DHX33<ILMN_1730059>	ENSG00000005100	DEAH (Asp-Glu-Ala-His) box polypeptide 33	ENSMUSG00000040620	NS			0.009331378	-0.051942507	0.529267725	0.640205908
SNRPE<ILMN_1695877>	ENSG00000182004	small nuclear ribonucleoprotein polypeptide E	ENSMUSG00000090553	NS			0.004756085	-0.051951392	0.529197182	0.640205908
LSM1<ILMN_1738956>	ENSG00000175324	LSM1 homolog, U6 small nuclear RNA associated (S. cerevisia	ENSMUSG00000037296	NS			0.000694772	-0.052587649	0.524158119	0.640205908
BCCIP<ILMN_1771966>	ENSG00000107949	BRCA2 and CDKN1A interacting protein	ENSMUSG00000030983	NS			0.022401791	-0.051484658	0.532909103	0.641807916
WDR75<ILMN_1801869>	ENSG00000115368	WD repeat domain 75	ENSMUSG00000025995	NS			0.028413335	-0.051088948	0.536066344	0.642815433
MEPCE<ILMN_1739616>	ENSG00000146834	methylphosphate capping enzyme	ENSMUSG00000029726	NS			0.044127924	-0.049348778	0.55006028	0.655023208
C19orf47<ILMN_1735608	ENSG00000160392	chromosome 19 open reading frame 47	ENSMUSG00000049643		0.040692458		0.019122041	0.049235638	0.55097625	0.655023208
FLYWCH2<ILMN_1805796	ENSG00000162076	FLYWCH family member 2	ENSMUSG00000023911	NS			0.028430832	-0.048825862	0.554299952	0.656158547
YTHDF1<ILMN_1753885>	ENSG00000149658	YTH domain family, member 1	ENSMUSG00000038848	NS			0.005462622	-0.048392531	0.557825229	0.657521619
DNAJC17<ILMN_1703573	ENSG00000104129	DnaJ (Hsp40) homolog, subfamily C, member 17	ENSMUSG00000034278	NS			0.012820412	0.047483768	0.565253174	0.663453968
ATP5C1<ILMN_1685774>	ENSG00000165629	ATP synthase, H+ transporting, mitochondrial F1 complex, ga	ENSMUSG00000025781	NS			0.008989339	-0.046777935	0.571054696	0.66743524
PCSK9<ILMN_1668850>	LRG_275	proprotein convertase subtilisin/kexin type 9	NA	NS			0.019939836	0.046447294	0.573781981	0.667805101
PCSK9<ILMN_1668850>	ENSG00000169174	proprotein convertase subtilisin/kexin type 9	ENSMUSG00000044254	NS			0.019939836	0.046447294	0.573781981	0.667805101
EIF4G1<ILMN_1672902>	ENSG00000114867	eukaryotic translation initiation factor 4 gamma, 1	ENSMUSG00000045983	NS			0.028059886	0.045470058	0.581878197	0.674394399
ZAK<ILMN_1714784>	NA	NA	NA		0.014685523		0.000344458	-0.044225188	0.592267551	0.680739109
PARP14<ILMN_1691731>	ENSG00000173193	poly (ADP-ribose) polymerase family, member 14	ENSMUSG00000034422	NS			0.004445444	-0.044289652	0.591727494	0.680739109
PPAN<ILMN_1803613>	ENSG00000130810	PPAN-P2RY11 readthrough	ENSMUSG00000004100	NS			0.040357776	-0.042417631	0.607501009	0.69250114
PPAN<ILMN_1803613>	ENSG00000130810	peter pan homolog (Drosophila)	ENSMUSG00000004100	NS			0.040357776	-0.042417631	0.607501009	0.69250114
PPRC1<ILMN_1796210>	ENSG00000148840	peroxisome proliferator-activated receptor gamma, coactivat	ENSMUSG00000055491	NS			0.017635786	-0.0424858	0.606923382	0.69250114
EEF2<ILMN_1738383>	ENSG00000167658	eukaryotic translation elongation factor 2	ENSMUSG00000034994	NS			0.015152925	-0.036649717	0.657224571	0.74254613
ILF3<ILMN_1698463>	ENSG00000129351	interleukin enhancer binding factor 3, 90kDa	ENSMUSG00000032178	NS			0.018887448	-0.036665119	0.657089644	0.74254613
EIF1AX<ILMN_1813240>	ENSG00000173674	eukaryotic translation initiation factor 1A, X-chromosomal-lik	ENSMUSG00000067194	NS			8.14E-08	-0.036396405	0.659445259	0.74254613
EIF1AX<ILMN_1813240>	ENSG00000173674	eukaryotic translation initiation factor 1A, X-linked	ENSMUSG00000067194	NS			8.14E-08	-0.036396405	0.659445259	0.74254613
PCBP3<ILMN_1687216>	ENSG00000183570	poly(rC) binding protein 3	ENSMUSG00000001120	NS			0.034160341	0.035773009	0.66492295	0.745682879
SRP19<ILMN_1728385>	ENSG00000153037	signal recognition particle 19kDa	ENSMUSG00000014504	NS			0.039232256	-0.034810449	0.673415639	0.752161779
RBM3<ILMN_1698213>	ENSG00000268489	RNA binding motif (RNP1, RRM) protein 3	NA	NS			0.045293037	0.034390242	0.677136176	0.75328003
RBM3<ILMN_1698213>	ENSG00000102317	RNA binding motif (RNP1, RRM) protein 3	ENSMUSG00000031167	NS			0.045293037	0.034390242	0.677136176	0.75328003
MRPL20<ILMN_1693352>	ENSG00000242485	mitochondrial ribosomal protein L20	ENSMUSG00000029066	NS			0.034486756	-0.034026633	0.680361895	0.753840985
EIF1AX<ILMN_1808344>	ENSG00000173674	eukaryotic translation initiation factor 1A, X-chromosomal-lik	ENSMUSG00000067194	NS			1.76E-19	-0.032765379	0.691595601	0.763234985
EIF1AX<ILMN_1808344>	ENSG00000173674	eukaryotic translation initiation factor 1A, X-linked	ENSMUSG00000067194	NS			1.76E-19	-0.032765379	0.691595601	0.763234985
RTF1<ILMN_1670647>	ENSG00000137815	Rtf1, Paf1/RNA polymerase II complex component, homolog (	ENSMUSG00000027304	NS			0.030727073	0.031327839	0.704481746	0.772719747
SF3B4<ILMN_1722648>	ENSG00000263977	splicing factor 3b, subunit 4, 49kDa	NA	NS			0.049814074	0.031184729	0.705769266	0.772719747
SF3B4<ILMN_1722648>	ENSG00000143368	splicing factor 3b, subunit 4, 49kDa	ENSMUSG00000068856	NS			0.049814074	0.031184729	0.705769266	0.772719747
GNL2<ILMN_1761113>	ENSG00000134697	guanine nucleotide binding protein-like 2 (nucleolar)	ENSMUSG00000028869	NS			0.038244539	-0.030340813	0.713378594	0.777975875
HMGB2<ILMN_1654268>	ENSG00000164104	high mobility group box 2	ENSMUSG00000054717	NS			0.041699356	0.026653071	0.746953086	0.811396113
RRP12<ILMN_1767253>	ENSG00000052749	ribosomal RNA processing 12 homolog (S. cerevisiae)	ENSMUSG00000035049	NS			0.027513501	-0.024414591	0.767572032	0.830536891
FAM50A<ILMN_1725130>	ENSG00000071859	family with sequence similarity 50, member A	ENSMUSG00000001962	NS			0.024140708	0.02110188	0.798382957	0.860513973
FAM50A<ILMN_1725130>	ENSG00000269645	family with sequence similarity 50, member A	NA	NS			0.024140708	0.02110188	0.798382957	0.860513973
GTPBP4<ILMN_1742577>	ENSG00000107937	GTP binding protein 4	ENSMUSG00000021149	NS			0.026756582	0.017573104	0.831547737	0.892785709
PTPN1<ILMN_1681591>	ENSG00000196396	protein tyrosine phosphatase, non-receptor type 1	ENSMUSG00000027540	NS			0.020533735	-0.016963334	0.837310559	0.89550207
BYSL<ILMN_1682792>	ENSG00000112578	bystin-like	ENSMUSG00000023988	NS			0.044860123	-0.014470768	0.860954083	0.917247253
UBC<ILMN_1891922>	ENSG00000150991	ubiquitin C	ENSMUSG00000008348	NS			0.037445816	-0.014095972	0.864520483	0.917517925

EIF1AX<ILMN_1755419>	ENSG00000173674	eukaryotic translation initiation factor 1A, X-chromosomal-lik	ENSMUSG00000067194	NS	2.66E-08	-0.01339731	0.871175901	0.921052383
EIF1AX<ILMN_1755419>	ENSG00000173674	eukaryotic translation initiation factor 1A, X-linked	ENSMUSG00000067194	NS	2.66E-08	-0.01339731	0.871175901	0.921052383
SYF2<ILMN_1660186>	ENSG00000117614	SYF2 pre-mRNA-splicing factor	ENSMUSG00000028821	NS	0.043626622	-0.012293153	0.8817123	0.921638895
RPL3<ILMN_1878285>	ENSG00000100316	RNA, U86 small nucleolar	ENSMUSG00000060036	NS	0.024132714	-0.012513521	0.879607727	0.921638895
RPL3<ILMN_1878285>	ENSG00000100316	small nucleolar RNA, C/D box 83B	ENSMUSG00000060036	NS	0.024132714	-0.012513521	0.879607727	0.921638895
RPL3<ILMN_1878285>	ENSG00000100316	ribosomal protein L3	ENSMUSG00000060036	NS	0.024132714	-0.012513521	0.879607727	0.921638895
RPUSD3<ILMN_1795383>	ENSG00000156990	RNA pseudouridylate synthase domain containing 3	ENSMUSG00000051169	NS	0.03925653	0.012579827	0.878974647	0.921638895
USP36<ILMN_1697227>	ENSG00000055483	ubiquitin specific peptidase 36	ENSMUSG00000033909	NS	0.017359924	-0.008978421	0.91346044	0.951235078
MOV10<ILMN_1725700>	ENSG00000155363	Mov10, Moloney leukemia virus 10, homolog (mouse)	ENSMUSG00000002227	NS	0.049836158	-0.008118249	0.921723528	0.956244979
MRPL21<ILMN_1744835>	ENSG00000197345	mitochondrial ribosomal protein L21	ENSMUSG00000024829	NS	0.015253216	-0.00683353	0.934080283	0.958548856
HTATSF1<ILMN_1683221>	ENSG00000102241	HIV-1 Tat specific factor 1	ENSMUSG00000067873	NS	0.046308592	0.006808043	0.934325594	0.958548856
YARS2<ILMN_1782635>	ENSG00000139131	tyrosyl-tRNA synthetase 2, mitochondrial	ENSMUSG00000022792	NS	0.044921395	0.007230867	0.930256778	0.958548856
GNL3<ILMN_1806106>	ENSG00000163938	small nucleolar RNA, C/D box 19B	ENSMUSG00000042354	NS	0.014138333	-0.006220925	0.93997819	0.960789525
GNL3<ILMN_1806106>	ENSG00000163938	guanine nucleotide binding protein-like 3 (nucleolar)	ENSMUSG00000042354	NS	0.014138333	-0.006220925	0.93997819	0.960789525
GPATCH4<ILMN_1694837	ENSG00000160818	G patch domain containing 4	ENSMUSG00000028069	NS	0.042585355	0.005821704	0.943823506	0.961173197
CLK3<ILMN_1766353>	ENSG00000179335	CDC-like kinase 3	ENSMUSG00000032316	NS	0.02977613	0.003979748	0.961580697	0.975669795
DHX29<ILMN_1697503>	ENSG00000067248	DEAH (Asp-Glu-Ala-His) box polypeptide 29	ENSMUSG00000042426	NS	0.02925341	-0.003044233	0.970607214	0.981234286
SKIV2L2<ILMN_1651513>	ENSG00000039123	superkiller viralicidic activity 2-like 2 (S. cerevisiae)	ENSMUSG00000016018	NS	0.02180905	-0.001013533	0.990212155	0.9927269
LSM10<ILMN_1751803>	ENSG00000181817	LSM10, U7 small nuclear RNA associated	ENSMUSG00000050188	NS	0.003710571	0.000819355	0.992087287	0.9927269
XPO5<ILMN_1759495>	ENSG00000124571	exportin 5	ENSMUSG00000067150	NS	0.019632489	-0.000753119	0.992726932	0.9927269

Supplementary Table 3

TFs associated with age

HSF4
FOXN4
CAMTA1
ZNF518B
MYNN
RLF
ZBTB33
ESRRG
PLAG1
SMAD4
ZNF800
VDR
HDX
ARID2
ZBTB46
MECP2
MAF
ZBTB4
POU2F2
ZMIZ2
GMEB1
RFX3
FOXP4
TFAP2B
SOHLH2
BPTF
KAT7
CDC5L
HOXA2
RELA
SPDEF
HOXA4
ID4
RORC
BDP1
MNT
EP300
SMAD3
OSR2
RBPJL
TFEB
ERI2

TRERF1
TSHZ2
ZNF614
ERG
ZNF354C
MLXIPL
TFDP2
DLX1
GLI3
ZNF43
ZNF331
ZNF93
KIAA2018
ZNF445
PBX1
MYC
ZNF208
TBX1
ST18
CERS3
ZNF19
ZNF613
ZNF317
L3MBTL1
TULP4
SP2
NR3C1
IRX6
TAL2
HHEX
MECOM
ARNTL2
TFCP2
FOXD4

NonRBPs associated with age

CDK18
DNAJC6
VDR
SMR3A
KRTCAP2
ERGIC1
MLC1
LGALSL
PYROXD2
STT3A
OTUD1
CNTN3
THOC3
ANKRD36B
COL9A3
CHGB
FOXP4
FASLG
HSD17B1
EGR4
GFM2
AHDC1
CXCR4
ARHGAP31
FAM73B
CSPG4
SLC52A1
PPIAL4A
PLAU
LDB3
DNAJC14
ADD3
MSL1
LRRC63
PELI2
SLC23A1
CYCS
HMOX1
SPATA31C2
GTF2A1
EP300
ST3GAL6-AS1
CPXM2
ZNF385D

NOS2
TREX2
C20orf195
CGNL1
ZNF783
HTR1A
NUP98
GPR107
RB1CC1
CDK14
ERG
ADCK1
PTPN18
SFRP5
TFDP2
EXOC4
ANKUB1
MYD88
BRAP
CHD9
C16orf54
HTR4
BAI3
NUP214
MAGEA2
ZNF208
C22orf46
SOX4
RNF41
C5orf24
TTTY15
BRCA2
CDK9
SMARCD1
TRAPPC12
NSF
LDLRAP1
C18orf8
LRRTM4
LYRM2
IRX6
CAPN13
HHEX
RHOT1
SLC5A7
PRIM1
PPIAP30

SDAD1P1
LHB
FAM83B
HDX
BCL2L11
KIAA1033
NAPSB
PSMA3
CRYGC
COMMD1
GINS1
MFGE8
TMEM182
YAF2
MYO1A
IL10
PKD2
MSL3
BTC
TRIQK
VPS26A
FAM203A
C22orf26
BPTF
HN1L
HOXA2
CEP63
COG6
SQSTM1
ZNF879
MESDC1
B3GNT9
ID4
FGGY
LINC00085
INA
MANEA
OLFM4
PTPN11
SMAD3
LNPEP
PCF11
USP46
DMXL2
SSTR2
FOXRED2
FMO3

PLAT
NLN
COPE
NPHP1
ANO5
KRT28
UTS2
SLC22A7
LINC00442
THAP11
ZNF43
DHRS12
TMPRSS5
ZNF271
WASIR2
DPH3
CYP3A7
KIAA2018
FBXO9
PIP4K2A
DENND1B
LINC00484
KNTC1
KCNAB2
DOK1
PLGLB2
SOD1
C12orf5
KCNA2
PLTP
GTF3C3
WHSC1L1
BCR
ISM1
SULT1A4
GPR161
TMEM191C
MT4
ZNF317
SERPINB4
ETNK2
ACSBG1
APOE
ANKRD36C
C12orf40
RAB37
ATP5G3

EMP2
TRAPPC11
FAM27E3
TYW5
SYT5
HINT2
MXRA7
SLC38A10
SLC15A4
CDKN1B
PTPN21
CALU
CCDC101
GPR75-ASB3
CLEC7A
IQGAP1
PIF1
ABCA1
SEMA3E
ATP1A4
CCL8
TNFSF10
TMEM255A
PLIN2
HS2ST1
CCDC82
RFX3
SCRN2
SOHLH2
MYH6
EXD2
RTN3
RALA
PPM1A
MUCL1
PCDH7
BCL2
PARVA
CAMTA1
DTYMK
PHC3
PPP1R8
AKAP12
FAM102B
SPDEF
EML4
AZGP1

HIST1H2BI
RHEB
SCT
ERVFRD-1
SLC35A2
CDCA4
PLEKHM1
DDN
KRTAP3-2
ARHGEF4
HIST1H4G
UBOX5
HSPA12B
FTMT
NEK8
IGLC1
ANHX
DNAH12
NLRP5
GPCPD1
PRTN3
OXGR1
ZNF331
IGF1R
TSEN15
GJB2
GHRL
BCKDHA
CCHCR1
SLC17A3
MAGEA6
C12orf52
NUCB1
MTAP
BAMBI
EBPL
ABCC5
TACR1
PAQR8
IQSEC1
MCHR2
TRIP12
ZMYND8
SLC30A1
TMBIM6
SPATA18
SP2

SLC6A8
TTC21A
MKL2
FCRL3
RASL11B
HYI
CRYZ
TAF11
TTI2
PALM2
ANK1
ABHD12
AIM1L
PMAIP1
KRT71
MLST8
ERO1LB
C1D
GTF2B
CDC6
KDELC1
GPR146
ARMC8
SPC25
CHI3L1
PPP2R1B
FAH
TFAP2B
GCA
MYH7
PKHD1
RNF144A
TMCO1
PDE6B
MUTYH
EMC3
GLA
CMTM3
RASD1
RELA
PRKCD
SYT11
ACAD10
ARAP1
COG3
HOXA4
IL17RA

AGPS
SLFN13
ANTXR2
PIN1P1
TXNDC9
SERHL2
C8orf17
OSR2
GSTP1
KANSL3
HHLA3
SMAGP
PANX2
ABCC11
CDHR5
ARHGAP5
GALNT18
PRR5
RB1
RASEF
CYP7A1
TSHZ2
FRYL
PTPRA
ARMCX6
ASAH1
ISPD
CCDC85C
TP53I11
LRRC25
NSFL1C
HBB
SRC
FAM175A
PBX1
DUSP18
C10orf107
FN1
KIAA1468
TIMELESS
ALPP
ADAM18
MLLT10
CC2D1A
BPHL
BCAP29
FGF19

PLXNB3
RIMBP3
FTL
PPIAL4C
ARIH2
NR3C1
CD3G
MRS2
MECOM
CLEC4C
MLL5
OAZ3
ARNTL2
BBS5
FAM194A
CHCHD7
DMAP1
COG2
OLFML2A
LYNX1
KIF19
TMEM140
CMBL
C14orf105
NKAPL
SLC39A3
C5orf55
FAM84A
GIPC3
GPR85
TPTE
GPR17
STK32B
NOTCH2NL
TIPIN
CA8
ZNF585B
DHRS4
SOCS2
SMC5
EXTL1
XKR6
DOK4
VPS54
PHF21B
KRTAP13-3
DCAF10

HPR
MNT
PLGLA
G6PC2
PLGLB1
TMEM115
FBLN1
ZMYND11
KIAA1161
TNF
LCK
P2RX6
INTS4
PAXBP1
MAP3K8
GDF2
SGOL1
TTLL7
SMG8
ZNF354C
SLC13A2
ADH7
KIAA1211
JPH2
NCR3
ZNF93
CDC23
OR4D5
VSIG1
MED24
HLA-DRB1
OR5P2
GPR25
TRAM2
RSPH1
SPON1
BLM
DNLZ
ITGB1
BRD2
AKR1C4
CA5B
DNAH3
CLEC2D
SDC4
DDI2
KIAA0930

HPS1
SNX1
TCF7L2
PRMT8
KIAA0319
ELN
WBSCR28
KIRREL3
P4HTM
KCNMB1
SLC39A11
CLDND1
GCAT
HLA-DMB
CSF3
P2RY8
HIST1H4L
KLHL26
OLFML3
WFIKKN1
TRAPPC2
KIAA1244
S100PBP
CUL4A
TBRG1
ADIPOR2
SCN4B
PTPMT1
AJUBA
KRTAP10-10
CACUL1
MAF
TRAK1
USP12
NPTN
ZMIZ2
TNFRSF1A
FRZB
STK32A
OR6N1
KRTAP2-4
NDUFS5
EPHB6
USP22
STARD9
SPP1
MYH10

MCFD2
HSD17B12
PCSK5
RORC
ADCK3
KCNC2
BDP1
FAM114A1
GPR174
CDCP1
RAB27A
ANKRD11
TIMM50
PER1
PTGS2
BMP7
KIF5A
PCYOX1
B2M
TFEB
DUSP1
MUC5AC
HDAC1
KIF20A
PPFIBP1
TMTC4
SPG7
ZNF614
NUDT4
ASTN1
MLXIPL
ENTHD2
MRPS22
MROH6
ATP6V1A
N6AMT1
INPP5B
SGOL2
SS18L1
PPID
RPAP2
CACNG2
SUN3
FBXW9
PNPLA1
MED26
CNOT7

CDC42
PREX1
WARS2
TBX1
TPM1
LINC00174
HIRIP3
TMEM169
NSUN7
CCND1
ZNF19
ZNF613
RAMP2-AS1
RSC1A1
PYCR1
CHTF18
TULP4
RS1
OAZ1
TAL2
AGBL5
PLEKHA1
ADSS
SLC4A2
KLHL11
TFCP2
DTWD1
FOXD4
FAM69C
PYGO1
LIG4
SMS
RNFT2
FBXO3
GPR89A
TOLLIP
ZFAND2A
FAM199X
TRPC4AP
TMEM61
LGALS8
C16orf70
ADAM22
CPA4
LMO1
IDE
PTPN14

TNFSF9
PWWP2B
IL1RAP
SNCA
CNTN5
ITK
ERLEC1
NDUFA10
NBPF14
PTX3
RSF1
AKR1A1
MON1A
ADRB2
PHPT1
TRIM9
HKDC1
PLP1
PRSS3
WSB1
CDKN2B
ZSWIM3
OSGEPL1
LINC00341
ERI2
HTR3E
SOCS4
FGFR4
NME3
PARVB
TRERF1
KIF9
ING1
SCARB1
DLX1
GLI3
CT47A1
GOLGA6L9
MACROD2
MAN2C1
TTC4
KPNA6
AK2
DHRS7B
HLA-DRB3
MYC
IGFBPL1

ASMTL-AS1
FRMD4A
ERGIC2
CERS3
PTH1R
RBP1
PLK4
APBB1IP
CBY3
NRG1
BCL2L13
TMEM53
GOSR1
ME1
SLC35F3
NUP50
FANCF
IVL
LDOC1L
PLOD2
WDR86-AS1
KDSR
CCDC78
HINT1
TADA1
HAVCR2
PYROXD1
EPN1
TMEM178A
ZNF280A
ARID2
CCDC112
ZBTB46
SYTL3
RGS18
F8
TMEM184B
CD274
SULT1C4
GSK3B
POU2F2
ZBTB4
ORAOV1
GMEB1
PPP4R4
RPL37
LRTM2

NCOA4
KCTD9
C3orf37
DAAM1
CCL2
KAT7
AMIGO1
MAPK1IP1L
PRNP
GFRA2
ALKBH7
SLC9B2
HEPACAM
ITGB5
HGF
MMP1
IL27
ZNF300P1
SRP9
GULP1
GPR75
C9orf62
RAP2B
RBPJL
OR10R2
IL6
USP1
WAPAL
GKN2
LRRC36
PTPN7
TRMT61A
UBE2Q2P1
DPY19L1
C10orf32
WDR66
TMEM206
CMTM7
CASKIN1
GALNT10
PDLIM5
VIPR2
ANP32D
INCENP
BMP8B
SLC16A10
ERVV-1

PROCA1
SLC6A3
ZNF445
SMURF2
STT3B
CEACAM3
ARHGEF5
C1orf109
PSG7
HMBS
PHF23
SERHL
ST18
TMEM89
IDH3A
MAGEA4
SNCB
GNB3
TIMM8A
RANBP3
SLC35B4
C7orf13
CCRL2
L3MBTL1
FBRS
GPR89B
IQCF5-AS1
RNLS
PGK1
EEF1A2
MTFR1
HORMAD2
RIOK2
GMDS
LOC652140
PTK2
CCDC45
GABRB3
PLAG1
TYMS
SHBG
KIAA1622
USP2
PGBD1
TIGD7
KIAA1737
GPX2

FOXN4
DNAH8
ASGR2
MYO7A
GGNBP2
FZD9
PIK3R4
CAMTA1
TSPYL5
MPZL2
RGS5
C9orf142
LOC654201
PER2
PELI2
LONRF1
CCNU
PNKP
MGC15875
SMAD4
GZMH
ACMSD
PYCR2
TRPV4
BAG5
FAT4
DCUN1D2
CLDN15
ABHD5
SIL1
PCDH24
CLDN23
PHLDA3
NAGS
MFSD7
AK2
C11orf80
GALK1
DHRS7B
PODXL2
ZFAND1
RAB11FIP5
ASTN2
BOLA2
TSPAN3
MGLL
C16orf52

LOC651278
REG1B
TRIM22
HSD17B14
LY6E
RPESP
KDELR3
ZNF800
Septin 10
ULK2
Gene
NRCAM
ZNF518B
KCNB1
CHPF
CYP2B7P1
KLHL3
RSBN1
RDH11
BLMH
NTRK2
SPAG9
AGRN
LOC645708
PPP4R4
FREM2
MYNN
FAH
MTA3
ALKBH7
PRMT7
HIST1H4K
HSF4
GSS
TMEM22
YOD1
FDXR
LGALS4
CBLN4
MGC40405
C18orf56
ITGAL
MOGAT3
CRTAP
TMEM8
PPM1D
SOX2-OT

PRAMEF10
DDB2
CYB561D2
VSIG2
C16orf45
PARP6
ESRRG
FLJ25530
EVI5L
C1orf51
SLC16A10
SLC3A1
MYEOV
TMEM154
H2AFY2
LOC440157
ZBTB33
EYA2
OTOP1
SEMA5A
USP35
MAPRE2
CCDC55
FLJ20152
SULF2
PDCD6IP
TSPO
EXOSC3
SLC22A5
ERICH1
SH3PXD2A
ASTE1
TRIM52
C14orf173
RLF
SAMD11
PTGES2

Supplementary Table 4

qPCR Data for RBP Expression During Liver Development											
Gene	Time Point	Animal Repeat	Cq Value T.R. 1	Cq Value T.R. 2	Cq Value Average	Gapdh Loading Control	ΔCq	ΔΔCq (Relative to PN2)	Fold Change Relative to PN2	Average Fold Change	SEM
		3	20.9331	20.8593	20.8962	14.4651	6.4311	2.5427	0.1716		
Srsf4	PN2	1	22.3036	22.4264	22.3650	15.1237	7.2414	0.5123	0.7011	1.0627	0.2696
		2	21.9313	21.9002	21.9157	15.0299	6.8858	0.1567	0.8971		
		3	22.3647	22.3230	22.3439	16.2837	6.0601	-0.6690	1.5899		
	PN14	1	21.4269	21.7578	21.5924	14.6908	6.9016	0.1725	0.8873	0.7839	0.0529
		2	21.8851	21.9259	21.9055	14.7654	7.1401	0.4110	0.7521		
		3	21.9438	22.1867	22.0653	14.8469	7.2184	0.4893	0.7124		
	PN28	1	21.1745	21.0224	21.0985	14.7077	6.3908	-0.3383	1.2643	0.7328	0.2801
		2	22.5661	22.4403	22.5032	15.0860	7.4172	0.6881	0.6207		
		3	22.3915	22.5339	22.4627	14.0604	8.4023	1.6732	0.3136		
	3 MON	1	21.9381	22.0741	22.0061	14.6049	7.4012	0.6721	0.6276	0.9654	0.1775
		2	21.5150	21.4261	21.4705	15.0388	6.4317	-0.2974	1.2289		
		3	21.5815	21.8617	21.7216	15.0487	6.6728	-0.0563	1.0398		
	8 MON	1	20.2633	20.5042	20.3838	13.7136	6.6702	-0.0589	1.0417	0.9434	0.0827
		2	20.0503	19.9035	19.9769	13.2612	6.7157	-0.0134	1.0093		
		3	20.3270	20.1538	20.2404	13.1512	7.0892	0.3602	0.7791		
	10 MON	1	21.4911	21.3834	21.4373	15.4402	5.9970	-0.7321	1.6610	2.1264	0.3845
		2	20.9088	20.8543	20.8816	15.6832	5.1984	-1.5307	2.8893		
		3	20.3450	20.3015	20.3233	14.4651	5.8582	-0.8709	1.8288		
Zmat3	PN2	1	24.2940	24.4811	24.3876	16.4809	7.9067	-0.0413	1.0291	1.0057	0.0743
		2	24.5382	24.2400	24.3891	16.2348	8.1543	0.2063	0.8668		
		3	25.0291	25.2536	25.1413	17.3581	7.7832	-0.1649	1.1211		
	PN14	1	24.3327	23.1675	23.7501	16.0317	7.7183	-0.2297	1.1726	0.8115	0.1811
		2	24.0840	24.0588	24.0714	15.4020	8.6694	0.7213	0.6066		
		3	24.1552	23.2701	23.7126	15.1547	8.5579	0.6098	0.6553		
	PN28	1	24.9795	25.0897	25.0346	15.3019	9.7327	1.7846	0.2903	0.2971	0.0109
		2	25.5227	25.6410	25.5819	15.8104	9.7715	1.8234	0.2826		
		3	24.5211	24.8011	24.6611	15.0617	9.5994	1.6513	0.3183		
	3 MON	1	24.4910	24.7736	24.6323	15.8019	8.8304	0.8824	0.5425	0.6444	0.0725
		2	24.5591	24.7954	24.6773	16.0064	8.6709	0.7228	0.6059		
		3	24.1642	24.2015	24.1828	15.8851	8.2978	0.3497	0.7848		
	8 MON	1	23.4887	23.7275	23.6081	15.2524	8.3557	0.4076	0.7539	0.8614	0.0624
		2	23.0527	23.0285	23.0406	14.8756	8.1650	0.2169	0.8604		
		3	22.8819	22.6892	22.7855	14.7935	7.9921	0.0440	0.9700		
	10 MON	1	24.7759	24.5906	24.6833	15.4402	9.2431	1.2950	0.4075	0.7306	0.1649
		2	23.7374	23.6738	23.7056	15.6832	8.0224	0.0743	0.9498		
		3	22.7603	22.5884	22.6743	14.4651	8.2093	0.2612	0.8344		
Sumo1	PN2	1	21.0968	21.3576	21.2272	15.1237	6.1036	0.1868	0.8786	1.0041	0.0629
		2	20.9836	20.7406	20.8621	15.0299	5.8321	-0.0846	1.0604		
		3	22.0541	22.1426	22.0984	16.2837	5.8146	-0.1021	1.0734		
	PN14	1	20.7068	20.7828	20.7448	14.6908	6.0540	0.1372	0.9093	1.0697	0.0815
		2	20.5920	20.4315	20.5118	14.7654	5.7463	-0.1705	1.1254		
		3	20.5062	20.5571	20.5317	14.8469	5.6848	-0.2320	1.1745		
	PN28	1	21.3013	21.2290	21.2652	14.7077	6.5575	0.6407	0.6414	0.7107	0.0521
		2	21.2190	21.3850	21.3020	15.0860	6.2160	0.2992	0.8127		
		3	20.5090	20.5664	20.5377	14.0604	6.4772	0.5605	0.6781		
	3 MON	1	21.0041	20.9812	20.9926	14.6049	6.3877	0.4710	0.7215	1.1039	0.2465
		2	20.9613	20.8768	20.9190	15.0388	5.8802	-0.0366	1.0257		
		3	20.2688	20.3705	20.3196	15.0487	5.2709	-0.6459	1.5647		
	8 MON	1	19.2572	19.1467	19.2019	13.7136	5.4883	-0.4285	1.3458	1.0420	0.1683
		2	19.1259	19.1851	19.1555	13.2612	5.8943	-0.0225	1.0157		
		3	19.5276	19.3832	19.4554	13.1512	6.3042	0.3875	0.7645		
	10 MON	1	20.0809	19.9165	19.9987	15.4402	4.5585	-1.3583	2.5638	2.9940	0.2209
		2	20.0744	19.6838	19.8791	15.6832	4.1959	-1.7209	3.2964		
		3	18.7904	18.6886	18.7395	14.4651	4.2744	-1.6423	3.1217		
Mrpl42	PN2	1	20.3840	20.2878	20.3359	15.1237	5.2122	0.0249	0.9829	1.0054	0.0743
		2	20.3919	20.3810	20.3864	15.0299	5.3565	0.1691	0.8894		
		3	21.3273	21.2268	21.2771	16.2837	4.9933	-0.1940	1.1439		
	PN14	1	19.4461	19.4190	19.4325	14.6908	4.7417	-0.4456	1.3619	1.4072	0.0599
		2	19.5591	19.5155	19.5373	14.7654	4.7719	-0.4155	1.3338		
		3	19.4266	19.4225	19.4246	14.8469	4.5777	-0.6097	1.5259		
	PN28	1	19.9615	19.9313	19.9464	14.7077	5.2387	0.0514	0.9650	0.8876	0.0794
		2	20.3322	20.3059	20.3190	15.0860	5.2330	0.0457	0.9688		
		3	19.7161	19.6919	19.7040	14.0604	5.6436	0.4562	0.7289		
	3 MON	1	20.2582	20.1754	20.2168	14.6049	5.6119	0.4246	0.7451	0.9578	0.1499
		2	19.9392	19.8758	19.9075	15.0388	4.8687	-0.3187	1.2472		
		3	20.4985	20.3388	20.4186	15.0487	5.3699	0.1825	0.8812		
	8 MON	1	19.6919	19.2220	19.4569	13.7136	5.7433	0.5560	0.6802	0.6218	0.0661
		2	19.0357	18.9105	18.9731	13.2612	5.7119	0.5246	0.6952		
		3	19.3986	19.3374	19.3680	13.1512	6.2168	1.0295	0.4899		
	10 MON	1	19.9675	19.7250	19.8463	15.4402	4.4061	-0.7813	1.7187	1.8988	0.3081
		2	19.5794	19.5187	19.5491	15.6832	3.8659	-1.3215	2.4993		
		3	19.0993	19.0775	19.0884	14.4651	4.6233	-0.5641	1.4784		

Supplementary Table 4

Gene	Time Point	Animal Repeat	Cq Value T.R. 1	Cq Value T.R. 2	Cq Value Average	Gapdh Loading Control	ΔCq	ΔΔCq (Relative to PN2)	Fold Change Relative to PN2	Average Fold Change	SEM
Cstf1	PN2	1	20.9084	20.7867	20.8476	16.4809	4.3667	-0.2716	1.2071	1.0102	0.1031
		2	21.2285	20.9566	21.0926	16.2348	4.8578	0.2195	0.8589		
		3	22.1021	21.9949	22.0485	17.3581	4.6903	0.0521	0.9646		
	PN14	1	21.3543	21.1751	21.2647	16.0317	5.2329	0.5947	0.6622	0.5505	0.1021
		2	21.0250	20.3310	20.6780	15.4020	5.2760	0.6377	0.6427		
		3	21.4128	21.2298	21.3213	15.1547	6.1666	1.5283	0.3467		
	PN28	1	21.9239	21.8455	21.8847	15.3019	6.5828	1.9445	0.2598	0.2718	0.0255
		2	22.1242	22.0539	22.0890	15.8104	6.2786	1.6403	0.3208		
		3	21.8453	21.7348	21.7900	15.0617	6.7283	2.0900	0.2349		
	3 MON	1	21.9395	21.5205	21.7300	15.8019	5.9282	1.2899	0.4090	0.4240	0.0459
		2	21.6259	21.6067	21.6163	16.0064	5.6099	0.9717	0.5099		
		3	22.0340	22.0154	22.0247	15.8851	6.1397	1.5014	0.3532		
	8 MON	1	21.3349	21.0372	21.1860	15.2524	5.9336	1.2953	0.4074	0.4210	0.0465
		2	20.5083	20.4763	20.4923	14.8756	5.6168	0.9785	0.5075		
		3	20.9551	20.9530	20.9540	14.7935	6.1606	1.5223	0.3481		
	10 MON	1	22.2687	22.1972	22.2329	15.4402	6.7927	2.1544	0.2246	0.2409	0.0403
		2	22.0314	21.9230	21.9772	15.6832	6.2940	1.6557	0.3174		
		3	21.6656	21.4763	21.5710	14.4651	7.1059	2.4676	0.1808		
MAP4	PN2	1	19.9888	19.9530	19.9709	16.4809	3.4901	-0.0795	1.0567	1.0351	0.1855
		2	20.3911	20.2319	20.3115	16.2348	4.0767	0.5071	0.7036		
		3	20.6777	20.3227	20.5002	17.3581	3.1420	-0.4276	1.3450		
	PN14	1	20.1463	19.8955	20.0209	16.0317	3.9892	0.4196	0.7476	0.6922	0.1166
		2	20.1272	20.0050	20.0661	15.4020	4.6641	1.0945	0.4683		
		3	19.3977	18.4838	18.9407	15.1547	3.7860	0.2164	0.8607		
	PN28	1	20.8472	20.7306	20.7889	15.3019	5.4869	1.9173	0.2647	0.2912	0.0567
		2	20.7786	20.6256	20.7021	15.8104	4.8917	1.3221	0.4000		
		3	20.9418	20.8376	20.8897	15.0617	5.8280	2.2584	0.2090		
	3 MON	1	21.0934	20.8962	20.9948	15.8019	5.1929	1.6233	0.3246	0.2993	0.0503
		2	21.1690	20.8437	21.0064	16.0064	5.0000	1.4304	0.3710		
		3	22.1947	21.3253	21.7600	15.8851	5.8749	2.3053	0.2023		
	8 MON	1	20.0183	19.8317	19.9250	15.2524	4.6726	1.1030	0.4655	0.4880	0.0610
		2	19.8165	19.7505	19.7835	14.8756	4.9079	1.3383	0.3955		
		3	19.6636	18.5217	19.0927	14.7935	4.2992	0.7296	0.6031		
	10 MON	1	21.1345	21.1161	21.1253	15.4402	5.6850	2.1154	0.2308	0.2881	0.0307
		2	21.0041	20.9963	21.0002	15.6832	5.3170	1.7474	0.2978		
		3	19.7627	19.4567	19.6097	14.4651	5.1447	1.5751	0.3356		
Mkrn3	PN2	1	29.6409	29.0795	29.3602	16.4809	12.8793	0.7775	0.5834	1.2506	0.6136
		2	27.2993	26.7576	27.0284	16.2348	10.7937	-1.3081	2.4762		
		3	30.1497	29.8315	29.9906	17.3581	12.6325	0.5306	0.6922		
	PN14	1	28.8549	28.8547	28.8548	16.0317	12.8231	0.7213	0.6066	0.5686	0.0199
		2	28.4801	28.3081	28.3941	15.4020	12.9921	0.8903	0.5395		
		3	28.2881	27.8992	28.0937	15.1547	12.9389	0.8371	0.5598		
	PN28	1	30.1020	29.9100	30.0060	15.3019	14.7040	2.6022	0.1647	0.2898	0.0629
		2	29.5015	29.4184	29.4600	15.8104	13.6496	1.5477	0.3420		
		3	28.8156	28.4372	28.6264	15.0617	13.5646	1.4628	0.3628		
	3 MON	1	30.5845	30.3601	30.4723	15.8019	14.6704	2.5686	0.1686	0.3095	0.0707
		2	29.5272	29.4086	29.4679	16.0064	13.4615	1.3597	0.3897		
		3	29.8772	28.9628	29.4200	15.8851	13.5349	1.4331	0.3703		
	8 MON	1	28.4611	28.3763	28.4187	15.2524	13.1663	1.0645	0.4781	0.5079	0.1005
		2	27.5559	27.4488	27.5023	14.8756	12.6268	0.5250	0.6950		
		3	28.5750	28.2396	28.4073	14.7935	13.6138	1.5120	0.3506		
	10 MON	1	28.2017	28.1722	28.1870	15.4402	12.7468	0.6449	0.6395	0.6153	0.2109
		2	27.8737	27.7906	27.8321	15.6832	12.1489	0.0471	0.9679		
		3	28.6759	28.5930	28.6345	14.4651	14.1694	2.0676	0.2386		
Ppia	PN2	1	17.0952	16.9761	17.0356	16.4809	0.5548	-0.1130	1.0815	1.0041	0.0629
		2	17.1136	17.0617	17.0877	16.2348	0.8529	0.1851	0.8796		
		3	18.0473	17.8605	17.9539	17.3581	0.5958	-0.0721	1.0512		
	PN14	1	16.0514	15.9777	16.0145	16.0317	-0.0172	-0.6850	1.6077	1.0685	0.2757
		2	16.3605	16.0878	16.2242	15.4020	0.8222	0.1544	0.8985		
		3	16.3864	16.2910	16.3387	15.1547	1.1840	0.5162	0.6992		
	PN28	1	16.6536	16.4789	16.5663	15.3019	1.2643	0.5965	0.6613	0.5659	0.0800
		2	17.1545	17.1374	17.1460	15.8104	1.3356	0.6677	0.6295		
		3	17.0383	17.0151	17.0267	15.0617	1.9649	1.2971	0.4069		
	3 MON	1	17.0735	16.7872	16.9304	15.8019	1.1285	0.4607	0.7266	0.7698	0.0216
		2	17.0482	16.9755	17.0118	16.0064	1.0054	0.3376	0.7913		
		3	17.1727	16.6079	16.8903	15.8851	1.0053	0.3375	0.7914		
	8 MON	1	16.1719	16.0559	16.1139	15.2524	0.8615	0.1937	0.8744	0.7755	0.0495
		2	16.0212	15.9867	16.0039	14.8756	1.1284	0.4606	0.7267		
		3	16.1460	15.7032	15.9246	14.7935	1.1311	0.4633	0.7253		
	10 MON	1	16.4131	16.3516	16.3824	15.4402	0.9421	0.2743	0.8268	0.6969	0.1099
		2	16.7105	16.6886	16.6995	15.6832	1.0163	0.3485	0.7854		
		3	16.2621	16.1307	16.1964	14.4651	1.7314	1.0635	0.4785		

Supplementary Table 5

A

Age Specific Mouse

Eartag	Strain	Sample ID	Gender	DOB	Collection Date	Age
N/A	FVB	PN2-1	?	?	?	2 days
N/A	FVB	PN2-2	?	?	?	2 days
N/A	FVB	PN2-3	?	?	?	2 days
N/A	FVB	PN14-1	?	?	?	2 weeks
N/A	FVB	PN14-2	?	?	?	2 weeks
N/A	FVB	PN14-3	?	?	?	2 weeks
N/A	FVB	PN28-1	?	?	?	4 weeks
N/A	FVB	PN28-2	?	?	?	4 weeks
N/A	FVB	PN28-3	?	?	?	4 weeks
N/A	FVB	3 MON-1	?	?	?	3 months
N/A	FVB	3 MON-2	?	?	?	3 months
N/A	FVB	3 MON-3	?	?	?	3 months
N/A	FVB	8 MON-1	Male	?	?	8 months
N/A	FVB	8 MON-2	Male	?	?	8 months
N/A	FVB	8 MON-3	Male	?	?	8 months
N/A	FVB	10 MON-1	Female			10 months
N/A	FVB	10 MON-2	Female			10 months
N/A	CD1	10 MON-3	Female			10 months

B

Table. Sequences of primers used for mRNA Expression

Age Dependent RBP Expression Primers

Gene Name	Fwd 5'-3'	Rev 5'-3'	Amplicon Size (bp)
SUMO1	AGAAGGAAGGAGAATACATTAAACTCA	AGAAACCTGAGTGAATTCATTGGA	148
SRSF4	CAGAAGCCGGAGTCACTCAA	CTCTTCGAATGGCTGCTTTT	96
ZMAT3	GCTAACAGCTGTCCTCCTCC	CTCTGTAGCCAGGATCACCC	129
MAP4	AATGGCCGACCTCAGTCTTG	CTCCACAGTTTCTCCCACGA	130
MKRN3	CTGTCGCTACTCTCACGACC	CTTGGGTCCTCTGTCTGCAG	91
PPIA	GCTCTGAGCACTGGAGAGAA	CTCTCCGTAGATGGACCTGC	132
MRPL42	ACGGAGCTTTGTCTAGTGCT	GGGATGTCCACAGAAGGGTG	133
CSTF1	AGCCACAGGATCTGCTGATG	GTTCGGATCACTGGGTGGTT	135

Supplementary Table 6

CLUSTER 1 -(pvalue=0.004)	CLUSTER 2 - (pvalue=0.026)
ILF3	SNRPD1
KPS26	CDC5L
FAV	PRPF8
RPL15	SNRNP40
RPL18A	
RPL22	
RPS27L	
SYNCRIP	
RPL10	
